# 2D Materials: From Design and Synthesis to Applications in Electrical and Electrochemical Biosensors

**DOI:** 10.1002/smll.202504955

**Published:** 2025-08-05

**Authors:** Masud, Jaeyoon Song, Falguni Ahmed, Jinsik Kim

**Affiliations:** ^1^ Department of Biomedical Engineering College of Life Science and Biotechnology Dongguk University Seoul 04620 Republic of Korea

**Keywords:** 2D materials, electrocatalysts, enzymatic and non‐enzymatic biosensors, field effect transistors‐based biosensor, top–down and bottom–up synthesis

## Abstract

This review critically analyzes various 2D materials—either synthesized or exfoliated from bulk 3D counterparts—for electrochemical and electrical biosensing applications. Each material exhibits unique electrochemical properties and benefits from its inherent 2D features, enabling abundant active sites for biomolecule interaction. Key challenges include synthesizing or exfoliating these materials and processing them for the cost‐effective and scalable production of biosensors. Additionally, the functionalization of 2D materials is crucial for effective bioreceptor immobilization, which directly affects selectivity, sensitivity, and overall performance. Certain 2D materials are better suited for specific sensing applications. For instance, 2D metal–organic frameworks or covalent organic frameworks show potential in electrochemical sensing due to their porous structures and high density of active sites. Transition metal dichalcogenides, such as MoS_2_ and WS_2_, show promise for field‐effect transistor‐based biosensors. Reduced graphene oxide and MXenes, with tunable surface functionalities, show promise for both electrical and electrochemical sensing platforms. Monoelemental 2D materials (Xenes) hold dual‐sensing potential, though synthesis and stability remain challenges for some. Hydrogenated Xenes offer improved stability, semiconducting behavior, and functionalization potential, making them strong candidates for biosensing. This review highlights these challenges and advantages while providing perspectives and future directions for optimizing 2D materials in biosensor development.

## Graphical abstract

Various types of 2D materials, encompassing all potential candidates to date, are examined alongside their synthetic methods and functionalization strategies for bioreceptor immobilization. This review highlights approaches for designing and scaling up these materials to enable cost‐effective electrical and electrochemical biosensing applications.

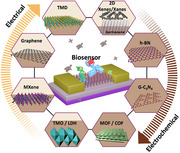

## 1 Introduction

Advancements in biosensor technology have consistently focused on miniaturization to enhance performance, reflecting principles analogous to Moore's Law in the semiconductor industry.^[^ ^1^, ^2^, ^3^, ^4^ ^]^ Intensive research into nanomaterials and their patterning at micro and nano scales has driven significant improvements in biosensing performance.^[^ ^5^, ^6^, ^7^, ^8^ ^]^ However, implementing such high‐precision techniques often requires expensive processes like electron beam lithography, which limits the production of cost‐effective and user‐friendly biosensors. This constraint poses a significant barrier to the widespread adoption of disposable biosensors, which are crucial for accessible point‐of‐care diagnostics and other applications requiring affordability and simplicity.

To address these challenges, ongoing research explores innovative strategies that balance miniaturization with cost efficiency while enhancing biosensing performance. Researchers have investigated efficient fabrication methods using nanomaterials, such as MEMS‐based manufacturing on wafer scales,^[^ ^4^, ^9^ ^]^ roll‐to‐roll printing,^[^ ^10^ ^]^ and 3D printing.^[^ ^11^, ^12^ ^]^ These techniques streamline material deposition and patterning processes, enabling large‐scale production and significantly reducing device costs. This cost‐efficient approach supports the development of disposable biosensors and point‐of‐care devices, which are essential for widespread adoption and accessibility. Extensive research into the use of 0D (e.g., metal nanoparticles),^[^ ^13^, ^14^ ^]^ 1D (e.g., nanowires and carbon nanotubes),^[^ ^15^, ^16^ ^]^ and 2D nanomaterials^[^ ^17^, ^18^ ^]^ in biosensor development highlights their unique properties.

2D materials, consisting of nanosheets just a few atomic or molecular layers thick, possess layer‐dependent physical, electrical, and optical properties that enable tailored performance. These attributes provide excellent conductivity and flexibility in electronic systems, along with a large active surface area, making them suitable for various applications, including electrocatalysis, biosensing, energy storage, and fields like electronics and optoelectronics.^[^ ^19^, ^20^, ^21^, ^22^ ^]^

**Figure 1:**
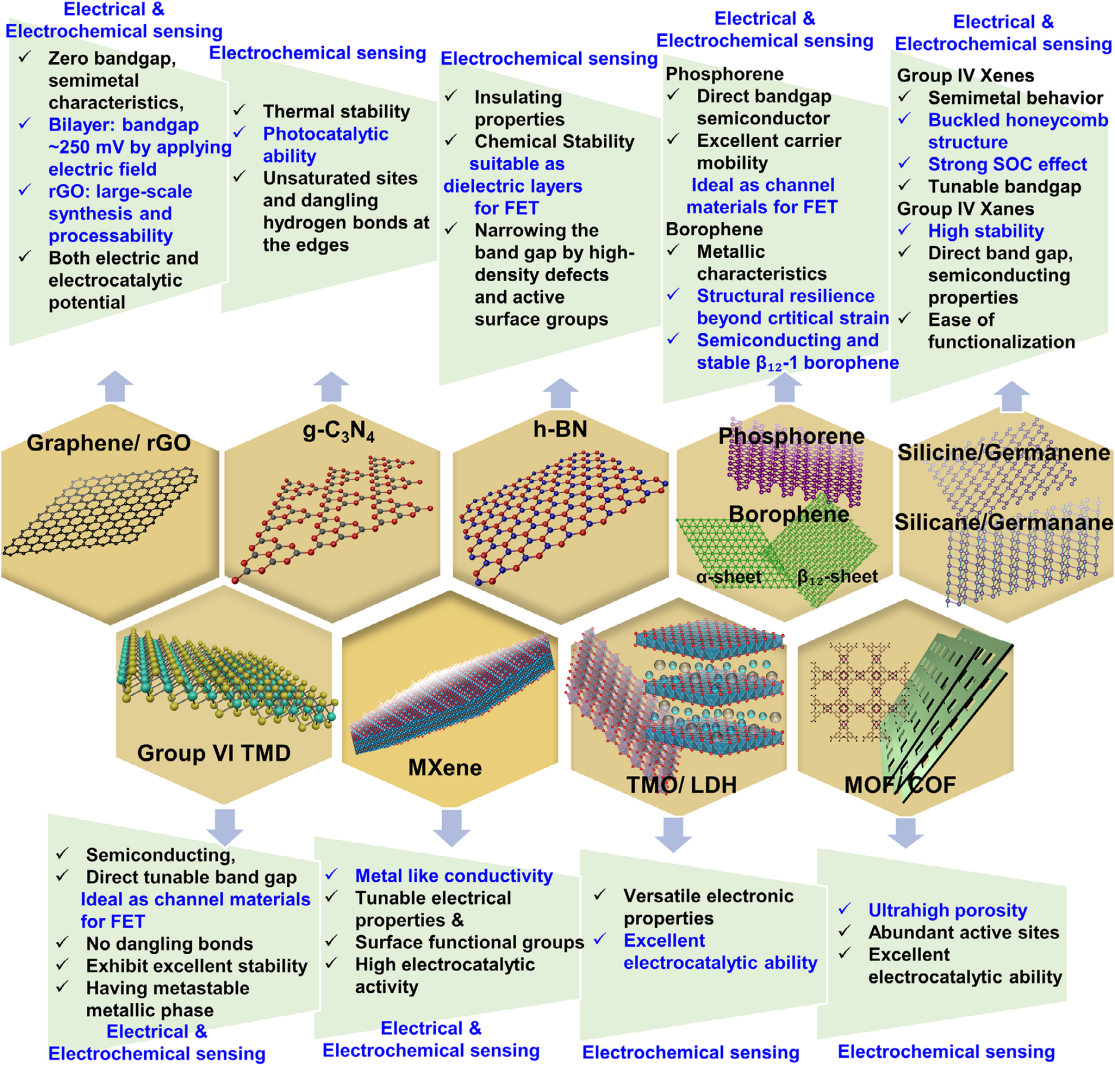
The unique properties of 2D materials highlight their potential for applications in electrical and electrochemical biosensing.

**Figure** **1** highlights the unique properties of various 2D materials that contribute to their potential in electrical and electrochemical biosensing applications.

Graphene is a single layer of carbon atoms arranged in a honeycomb‐like lattice. After the first isolation of graphene from graphite by Novoselov et al. in 2004,^[^ ^23^ ^]^ early research on graphene‐based electrical sensors, such as field‐effect transistors (FETs), began evolving in 2007.^[^ ^24^, ^25^, ^26^ ^]^ Since then, studies on biosensors have expanded to include a wide range of 2D materials, each with unique structures, morphologies, and properties. Transition metal dichalcogenides (TMDs), such as MoS_2_, WS_2_, and MoSe_2_, have a stable 2H semiconducting phase^[^ ^27^, ^28^ ^]^ and show potential for FET^[^ ^29^, ^30^ ^]^ biosensors. However, challenges such as hydrophobicity, reduced carrier mobility, and limited bioreceptor immobilization (via covalent bonding) make them less favorable compared to graphene and reduced graphene oxide (rGO).

MXenes—2D transition metal carbides, nitrides, and carbonitrides—were first synthesized from MAX phases in 2011,^[^ ^31^ ^]^ and numerous varieties of this class of 2D materials continue to emerge. Their metallic conductivity and hydrophilic surfaces make them attractive for biosensing applications.^[^ ^19^ ^]^ However, their solution‐phase stability is often lower than that of MoS_2_ and rGO due to oxidation under ambient conditions. 2D metal–organic frameworks (MOFs) and covalent organic frameworks (COFs)^[^ ^32^, ^33^ ^]^ show promise for electrochemical biosensing due to their porous structures and electrocatalytic activity, though their low conductivity remains a limitation. Similarly, 2D transition metal oxides (TMOs) are catalytically active and stable but suffer from poor conductivity. Semiconducting TMOs, such as α‐MoO_3_, can be effective channel materials for FET biosensors.^[^ ^34^ ^]^ Layered double hydroxides (LDHs), with their positively charged layers,^[^ ^20^ ^]^ are suitable for immobilizing negatively charged bioreceptors and offer electrocatalytic benefits, but their low conductivity is a drawback. Hexagonal boron nitride (h‐BN), which is thermally conductive and electrically insulating,^[^ ^35^ ^]^ is better suited as a dielectric or encapsulation layer in FETs rather than as an active sensing element. Graphitic carbon nitride (g‐C_3_N_4_) is especially effective in photoelectrochemical biosensors due to its photocatalytic properties.^[^ ^36^ ^]^ Monoelemental 2D materials beyond graphene—such as phosphorene, borophene, silicene, and germanene—are collectively known as Xenes. These materials are particularly attractive due to their tunable band gaps and excellent carrier mobility.^[^ ^37^ ^]^ Phosphorene is especially promising for FET biosensors owing to its direct bandgap, high carrier mobility, and good biocompatibility. Borophene, with its metallic behavior, is suitable for electrochemical biosensing; however, its stability remains a concern. On the other hand, Group‐IV Xenes (e.g., silicene and germanene) possess buckled honeycomb structures, semimetallic behavior, strong spin–orbit coupling (SOC), and easily tunable bandgaps, making them potential candidates for flexible electronics and electrical biosensors. Hydrogenated Xenes (Xanes), such as germanane, offer enhanced environmental stability and ease of functionalization, making them more promising for both electrical and electrochemical biosensing applications.

Overall, exploring this diverse family of 2D materials—through strategies like hybridization, heterostructure formation, doping, and functionalization—can significantly advance the design and performance of electrical and electrochemical biosensors, while helping to overcome the inherent limitations of each material.

To construct cost‐effective and scalable electrical and electrochemical biosensors using techniques such as screen printing, spin coating, and spray coating, solution‐processable 2D materials and their hybrids are particularly important. The most significant challenge for 2D materials is achieving large‐scale synthesis for the mass production of devices. This can be addressed through either efficient exfoliation from their 3D bulk materials (top–down approaches) or bottom–up synthesis methods, such as epitaxial vapor deposition or wet chemical synthesis. Top–down approaches include micromechanical cleavage, liquid‐phase exfoliation, and intercalation‐assisted exfoliation, which involve applying forces sufficient to overcome the van der Waals forces between stacked layers. Epitaxy refers to the process of depositing a monocrystalline film onto a monocrystalline substrate, ensuring precise structural alignment. Several thin‐film deposition techniques exist, including chemical vapor deposition (CVD),^[^ ^38^ ^]^ molecular beam epitaxy (MBE),^[^ ^39^ ^]^ pulsed laser deposition (PLD),^[^ ^40^ ^]^ and atomic layer deposition (ALD).^[^ ^41^ ^]^ In terms of cost, effectiveness, scalability, uniformity, and crystallinity of the grown materials, CVD is often the most effective technique for obtaining high‐quality 2D nanosheets suitable for electronic and optoelectronic devices. However, the sophisticated instrumentation, various controlling parameters (including high temperatures), and the complex transfer processes limit these techniques from being widely adopted in research laboratories. On the other hand, wet chemical synthesis is more suitable for large‐scale production.

In this paper, potential 2D materials isolated or synthesized to date, each possessing unique properties along with the advantages of 2D morphology, are described (Figure 1). While significant progress has been achieved with some common 2D materials, many others remain underutilized or insufficiently analyzed by researchers in this field. This review aims to explore the diversity of 2D materials, highlighting their characteristics and significance, from synthesis to application in cost‐effective electrical and electrochemical biosensing devices. It also discusses various functionalization strategies and optimization techniques for effective bioreceptor immobilization on 2D platforms, which are key factors in enhancing biosensing performance by selectively capturing target biomarkers. The review emphasizes the vast potential for further research in the synthesis and optimization of these materials, not limited only to electrical and electrochemical biosensing applications but also for other emerging technologies in electronics and optoelectronics.

## 2 Electrical and Electrochemical Biosensing Platforms

### 2.1 Electrochemical Biosensor

**Figure 2:**
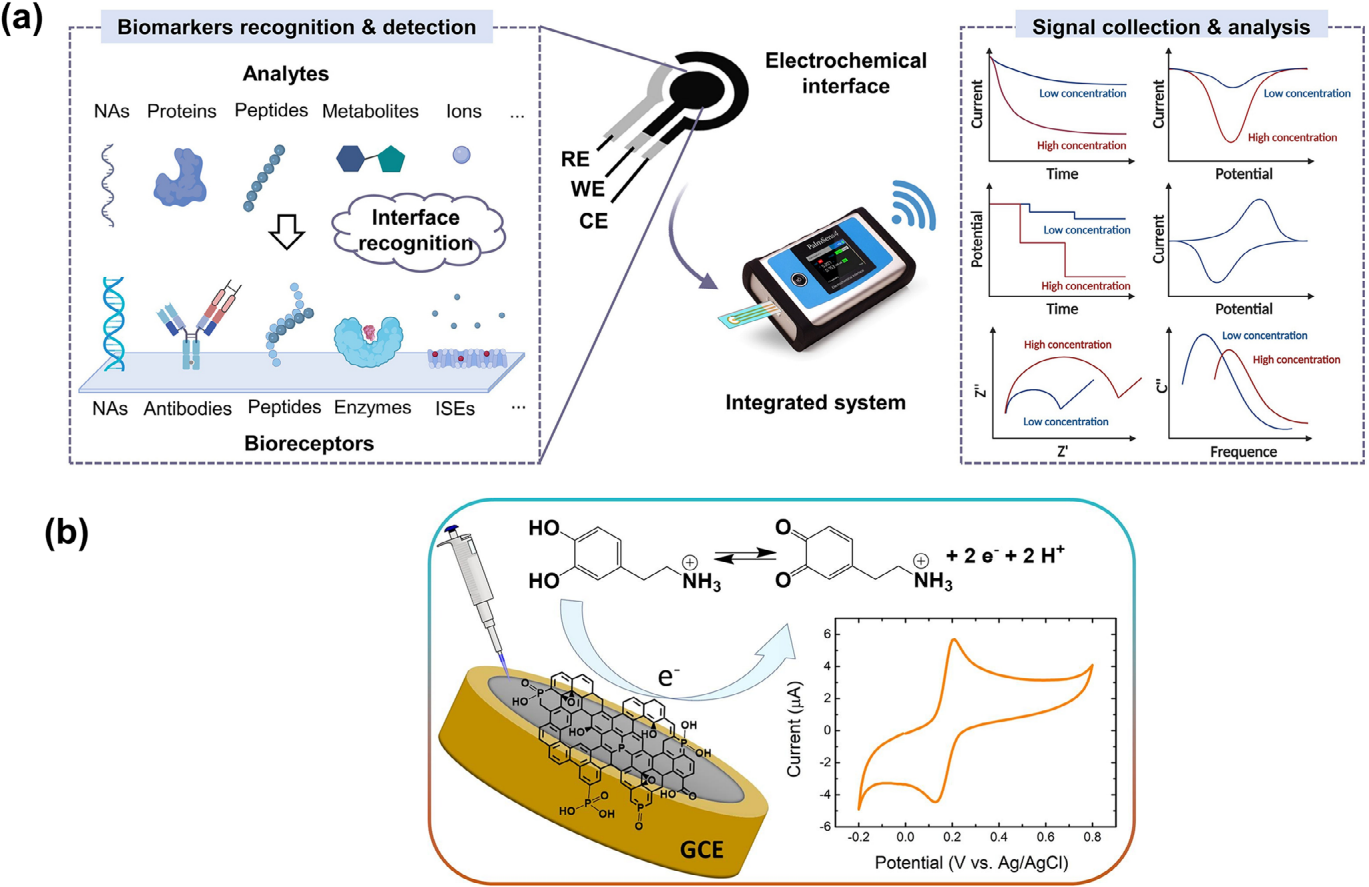
a) Schematic representation of the mechanism of an electrochemical biosensor, from biomarker recognition and detection to signal collection and analysis. Reproduced under terms of the CC‐BY‐NC‐ND license.^[^ ^42^ ^]^ Copyright 2024, Wang et al. b) Mechanism of a non‐enzymatic electrochemical biosensor for dopamine detection based on a functionalized rGO electrocatalytically active electrode. Reproduced with permission.^[^ ^43^ ^]^ Copyright 2022, Elsevier.

Electrochemical biosensors are sensing devices that consist of an electrode with a sensing layer immobilized with bioreceptor elements, which react with the target, causing electrochemical changes (e.g., electric current, potential, conductivity) that can be detected by electrochemical transducing methods, such as potentiometric, impedimetric, conductometric, and amperometric techniques. These biosensors can have either two or three electrodes. In a three‐electrode system, the working electrode is typically coated with 2D nanosheets or a nanocomposite, serving as a platform for the functionalization or immobilization of the bioreceptor, which captures the target and induces electrochemical changes in the device (**Figure** **2**a).^[^ ^42^ ^]^ In a two‐electrode system, the sensing layer (typically a 2D nanosheet) can be connected to two electrodes, and the electrochemical change (e.g., conductivity or resistance) of the device can be measured after the immobilization of a bioreceptor and its reaction with the target species. Effective immobilization of the bioreceptor on the 2D nanosheets is critical for enhancing device sensitivity. The sensitivity, selectivity, reproducibility, and specificity of the biosensor depend on the quality, surface chemistry, and functionalization ability of the 2D nanosheet or nanocomposite film. In electrochemical biosensors, enzymes are typically used as bioreceptors to detect target analytes such as glucose, hydrogen peroxide, dopamine, ascorbic acid, and uric acid, which directly impact human health. However, enzymes are expensive and require an additional immobilization step. In non‐enzymatic electrochemical biosensors, a 2D sensing layer acts as an electrocatalyst for the redox processes of the analytes (Figure 2b).^[^ ^43^ ^]^ Essentially, 2D materials with high electrocatalytic activity can detect the analytes by catalyzing the redox reactions associated with them.

Integrating a microfluidic system into the biosensor can simplify sample preparation, allowing for the direct placement of patient fluids, such as blood or plasma.

### 2.2 Field‐Effect Transistor‐Based Biosensor

**Figure 3:**
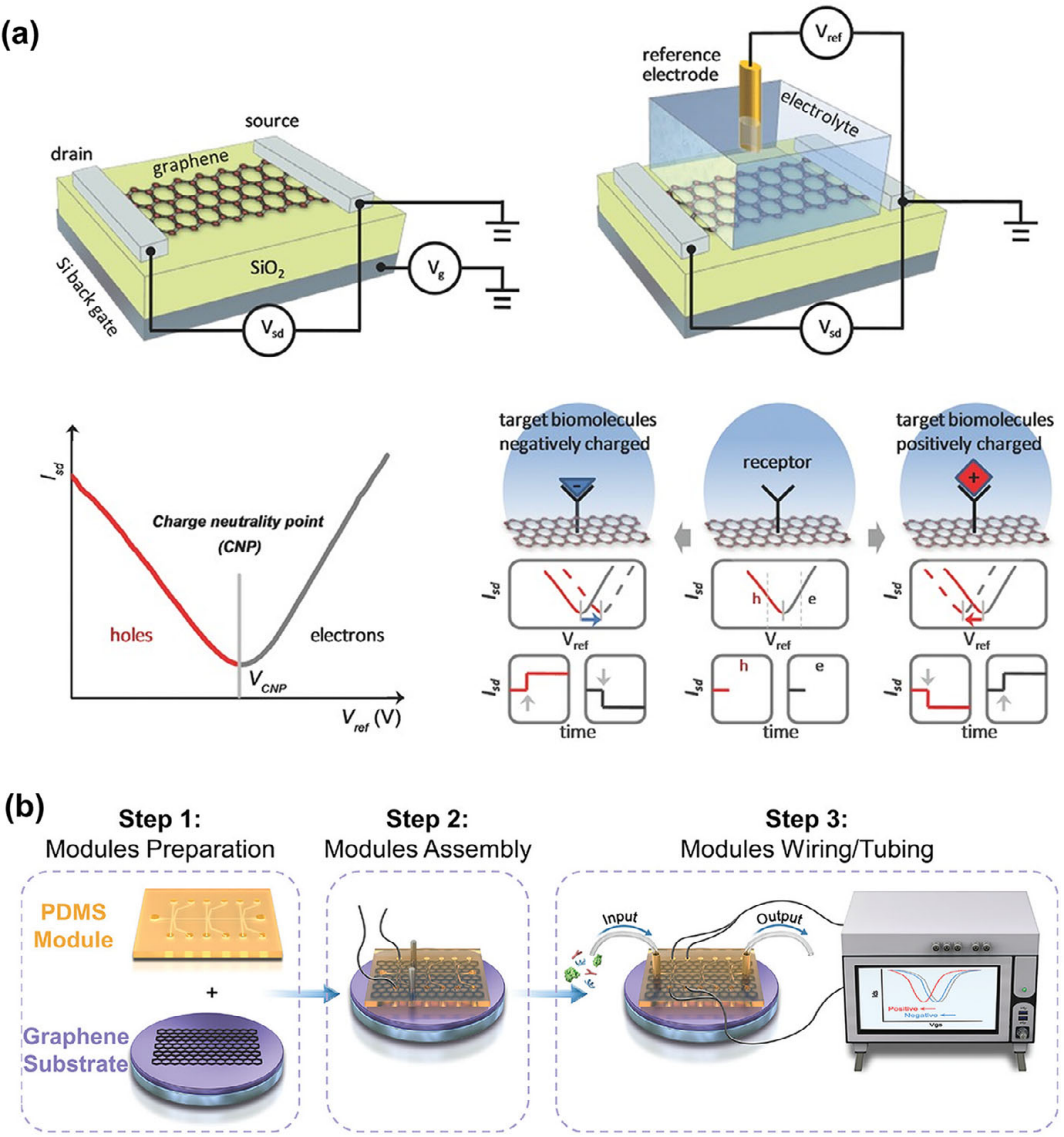
a) Device structure, including dielectric‐gated and electrolyte (liquid)‐gated FETs, and the working principle of FET‐based biosensors using 2D channel materials (showing changes in source–drain current, *I*_sd_, with respect to gate voltage and time). Reproduced with permission.^[^ ^44^ ^]^ Copyright 2017, Wiley. b) Schematic representation of a recently developed fabrication procedure for an FET‐biosensor integrated with a microfluidic channel. Reproduced under terms of the CC‐BY license.^[^ ^46^ ^]^ Copyright 2024, Zhang et al.

A Field‐Effect Transistor (FET) comprises two metallic electrodes, known as the source and drain, which are connected by a thin semiconducting channel (e.g., 2D MoS_2_, graphene, reduced graphene oxide (rGO), etc). The conductivity of this channel is modulated by an electric field. This electric field can be applied either through a conductive silicon substrate via an insulating dielectric layer (e.g., silicon dioxide, silicon nitride) (see dielectric back‐gate graphene FET device in **Figure** **3**a) or through a reference electrode immersed in electrolytes (see electrolyte top‐gate graphene FET in Figure 3a), within a specific voltage range.^[^ ^44^ ^]^ The primary benefits of dielectric‐gated FETs are their stability in various environments, wide operating temperature range, and capacity to endure electric fields up to several volts per nanometer.

Both electrolyte and dielectric gating mechanisms modulate the charge carrier density in 2D materials by forming a capacitor, but they do so in fundamentally different ways. Electrolytes, which conduct ions, become polarized only within a very thin interfacial layer (≈1 nm). In contrast, the entire volume of the dielectric material must polarize. As a result, the geometric capacitance of the electrolyte is significantly higher than that of the dielectric for the same applied voltage. This higher capacitance makes the electrolyte gate more energy‐efficient than the dielectric gate, as nearly all the applied voltage effectively shifts the Fermi level of the 2D material.^[^ ^45^ ^]^

The FET‐based biosensor (bio‐FET) is a highly promising sensing device. It operates by detecting changes in the conductivity of semiconducting channel materials caused by charged biomolecules in the presence of an external electric field. Receptor molecules are immobilized on the surface of the semiconducting channel layer for selective recognition of target biomolecules. In a typical measurement, a constant bias voltage (*V*_sd_) is applied through the gate between the source and drain of the 2D semiconducting channel, and the resulting source‐drain current (*I*_sd_) is monitored. By altering the gate voltage (*V*_g_), the electrochemical potential of charge carriers (i.e., the Fermi energy) can be adjusted, allowing for continuous tuning of the type of charge carriers (holes to electrons) in the 2D semiconducting channel. At the transition between electron and hole regimes, current is minimized, known as the charge neutrality point (CNP). Upon binding of a positively charged target, there is a depletion of hole carriers or accumulation of electron carriers in the semiconducting channel, resulting in a negative shift in the *I*_sd_–*V*_ref_ curve. While binding to a negatively charged target, a positive shift in the *I*_sd_–*V*_ref_ curve is observed (Figure 3a). In the time‐dependent measurement, the binding of a positively charged molecule causes a decrease in the current *I*_sd_ in the hole regime, and an increase in the current in the electron regime. Conversely, the binding of a negatively charged molecule induces an increase in the *I*_sd_ in the hole regime and a decrease in the electron regime.^[^ ^45^ ^]^

This current modulation in the semiconducting channel (Δ*I*_sd_) is related to the change of carrier density (∆n), which depends on the total number of charged biomolecules (N) adsorbed on the surface of channel materials as follows:^[^ ^44^, ^45^ ^]^

(1) $$\Delta I_{\mathrm{sd}}=\frac{w}{l}V_{\mathrm{sd}}e\mu \Delta n\propto N$$

Where w, l, and µ are the width, length of the channel, and charge carrier mobility of channel materials. e is the electron charge.

Sensing response of bioFET depends on the no. of adsorbed biomolecules and Δ*I*_sd_. Since Δ*I*_sd_ depends on the mobility of semiconducting channel materials, a 2D channel material with high charge carrier mobility (e.g., high‐quality mono or few‐layered graphene) is the preferential requirement for better sensing response and better sensing performance in bioFET.

It is noted that non‐charged biomolecules do not affect the FET sensing response unless it is capable of inducing charge variation. There is also a possibility to change the mobility of channel material due to the direct adsorption of biomolecules on the channel surface instead of binding to the receptor immobilized on the surface of the channel layer.

To achieve clinical viability for BioFETs using 2D materials, several key challenges must be addressed: improving reproducibility and manufacturing yield, mitigating Debye screening effects, and preventing the undesired detection of nonspecific species. The integration of microfluidic channels with FET sensors can be a good option for constructing lab‐on‐a‐chip systems to handle samples for point‐of‐care testing in clinical diagnostics (Figure 3b).^[^ ^46^ ^]^ However, ensuring target detection in the presence of various non‐targets in patient samples can be the main challenge.

## 3 2D Materials: Characteristics, Importance, Large‐Scale Production, and Applications in Electrical and Electrochemical Biosensing

### 3.1 Graphene‐Based 2D Materials

Graphene is a flat single layer of sp^2^ carbon atoms, tightly packed into a 2D honeycomb lattice. Graphene is the basic building block for graphitic materials: fullerene (0D), nanotubes (1D), and graphite (3D).^[^ ^47^ ^]^

#### 3.1.1 Importance of Graphene‐Based 2D Materials

**Figure 4:**
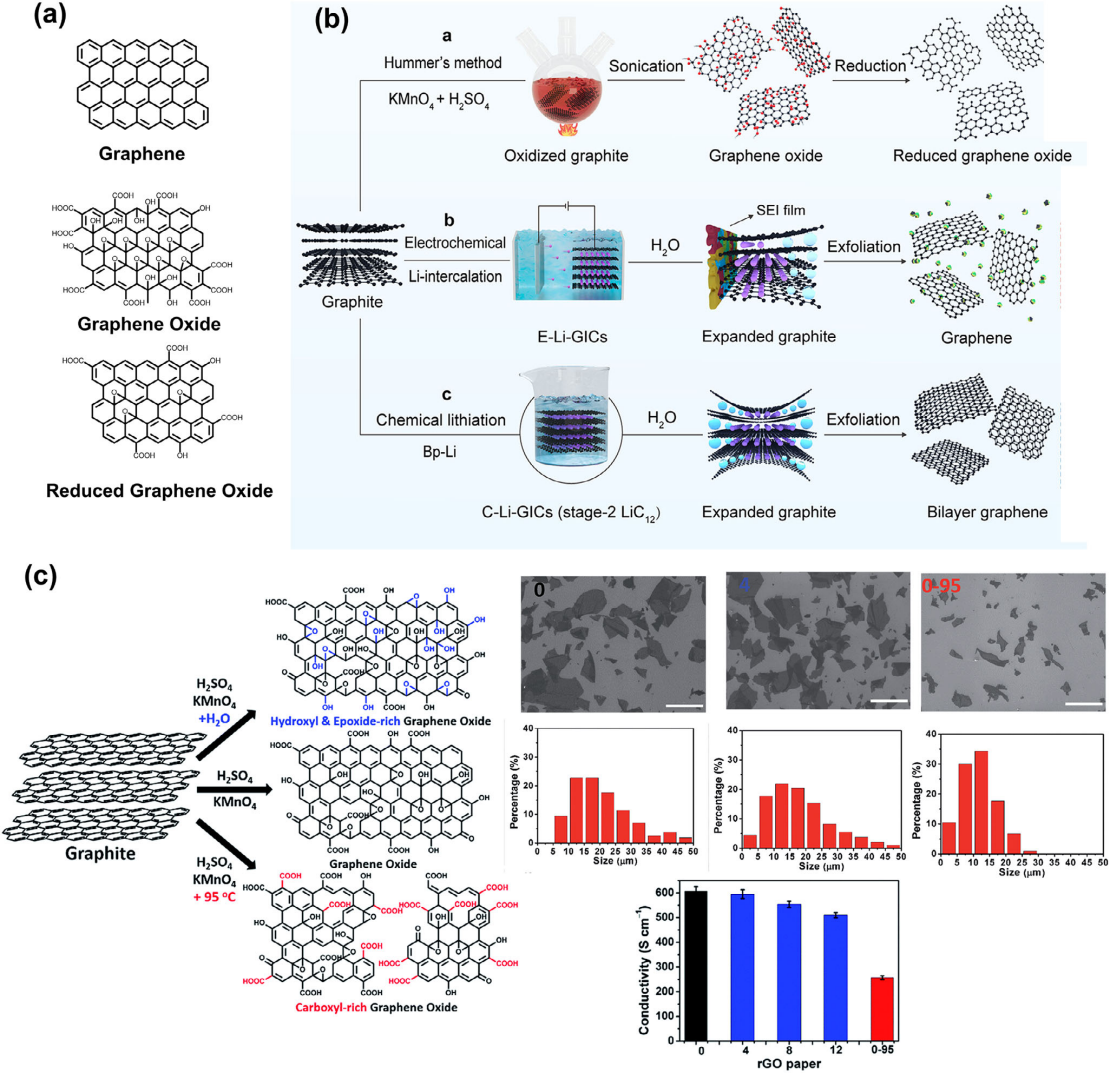
a) Probable chemical structures of graphene‐based 2D materials. b) Three synthetic routes for obtaining Graphene‐like 2D material: Oxidation of graphite by Hummer's method, electrochemical Li‐intercalation‐exfoliation, chemical lithiation‐exfoliation. Reproduced with permission.^[^ ^58^ ^]^ Copyright 2024, ACS. c) Controlled oxidation for obtaining graphene oxide with tunable functional groups, SEM images with flake percentage, and conductivity of reduced graphene oxide based on controlled oxidation in the presence or absence of water. Reproduced under the terms of a Creative Commons Attribution 3.0 Unported License.^[^ ^59^ ^]^ Copyright 2016, Chen et al.

The high theoretical specific surface area (≈2630 m^2^g^−1^), outstanding intrinsic mobility (200000 cm^2^v^−1^s^−1^), high Young's modulus (≈1.0 TPa), good thermal conductivity (≈5000 Wm^−1^K^−1^), and optical transmittance (≈97.7%) of graphene make it promising for various electronic devices and sensors.^[^ ^48^ ^]^ The physicochemical properties of graphene depend on the number of layers due to its unique electronic band structure. The electronic bandgap of nanosheets determines the transport and optical properties.^[^ ^49^ ^]^ Monolayer graphene has no band gap; however, bilayer graphene shows a tunable band gap up to 250 mV with a perpendicular electric field.^[^ ^49^ ^]^ Therefore, bilayer graphene can be promising for highly efficient electronic and photonic devices. Despite the potential electronic properties of graphene that make it suitable for various electronic applications, the large‐scale industrial production and processing of graphene remain quite challenging. Reduced graphene oxide (rGO), a chemically modified form of graphene, represents a promising alternative for utilizing graphene‐like materials in various electronic devices.^[^ ^50^ ^]^ Graphene oxide (GO) serves as the precursor of rGO. Because of the scalable synthesis, various functionalization, and easy deposition on various substrates, GO is the most promising precursor of the chemically modified form of graphene. The exact structure of GO is still debated;^[^ ^51^, ^52^, ^53^, ^54^, ^55^, ^56^ ^]^ however, the most accepted model is the Lerf–Klinowski model^[^ ^55^ ^]^ described in 1996 based on the analysis of ^13^C and proton NMR. According to this model, GO consists of nearly flat carbon grids where only the carbons attached to OH groups may exhibit a slightly distorted tetrahedral configuration. The model describes GO as having two randomly distributed regions: aromatic regions with unoxidized sp^2^‐hybridized graphene, and oxidized regions with aliphatic sp^3^ six‐membered rings. The size of these two regions depends on the degree of oxidation. The oxygen‐containing functional groups include epoxides (C–O–C) and hydroxyls (─OH) primarily on the basal plane, while carboxyl (─COOH) and hydroxyl groups are located at the edges of the graphene sheets (**Figure** **4**a).^[^ ^55^ ^]^ Due to functionalization through oxidation, GO can become hydrophilic and readily disperse in aqueous or polar organic media, forming uniform films. The processing of GO is quite convenient for aqueous or organic solution‐based device fabrication.

#### 3.1.2 Current Synthetic Methods and Challenges in the Large‐Scale Production of Graphene‐Based 2D Materials

##### Mechanical Exfoliation

In 2005, the first few‐atom‐thick 2D graphene was reported through mechanical exfoliation (repeated peeling) of small mesas of highly oriented pyrolytic graphite (HOPG) by scotch tape.^[^ ^23^ ^]^ Even important intrinsic properties, such as the quantum Hall effect, massless Dirac fermions, and superconductivity, can be preserved through mechanical exfoliation of graphene, whereas these properties may be suppressed or not preserved by other exfoliation techniques.^[^ ^57^ ^]^ Not only graphite, but also various kinds of layered crystals can be peeled into single or few‐layered flakes using this simple procedure. This method can produce single‐layer or few‐layer 2D materials with minimal defects and high quality, and it applies to a wide range of layered materials (e.g., graphite, BN, MoS_2_, NbSe_2_, Fe3GeTe_2_).

However, the main limitations of this exfoliation technique are its low yield, small flake size, and lack of uniformity.

##### Chemical Intercalation

Layered materials can be intercalated with different types of intercalants, such as alkali metal ions (e.g., Li, Na, K), organic molecules, metal halides, polymers, etc., due to their lamellar structure. This chemical intercalation weakens the weak van der Waals interactions among stacked layers and expands the interlayer distance. As a result, exfoliation into single‐ or few‐layered 2D materials can be achieved more easily through various exfoliation processes in different organic and inorganic media. This top–down exfoliation technique is known as chemical intercalation and exfoliation. Recently, the Zhu group used biphenyl lithium to intercalate and exfoliate graphite to bilayer graphene (Figure 4b).^[^ ^58^ ^]^ Biphenyl lithium is a strong reducing agent that can react directly with graphite at ambient temperature. The lithium intercalation process of graphite is quite complex and can occur in multiple stages. Second‐order lithiation may occur with biphenyl lithium due to its lower redox potential (+ 0.11 V) compared to the stage‐2 lithium graphite intercalation compounds (0.125 V). They claimed that the bilayer graphene they produced has high quality, excellent structural integrity, a low carbon‐to‐oxygen ratio (≈29.7), and that their procedure can achieve more than 70% selectivity for bilayer graphene. A lower C/O ratio indicates less oxidation and better conductivity, and higher quality of the exfoliated graphene.

##### Electrochemical Intercalation

Electrochemical intercalation and exfoliation of graphite are typically carried out using a two‐electrode system, where bulk graphite serves as the working electrode and a platinum or copper foil acts as the counter electrode.^[^ ^60^, ^61^, ^62^ ^]^ By applying a DC voltage, usually between +5 V and +10 V, various ions or small molecules from the electrolyte intercalate between the graphite layers. This process reduces the π–π interactions among the stacked layers and expands the interlayer distance. During the electrochemical reduction process, gases formed by the intercalated species further expand the layers, leading to the exfoliation of single or few‐layer graphene. The exfoliated graphene can then be dispersed in an organic or inorganic solvent to create graphene ink or a dispersed solution for solution‐processed conductive graphene films. Different types of electrolytes, such as ionic liquid electrolytes,^[^ ^63^ ^]^ aqueous inorganic acids (e.g., H_3_PO_4_ or H_2_SO_4_),^[^ ^60^, ^64^ ^]^ and aqueous inorganic salts (e.g., (NH_4_)_2_SO_4_ or Na_2_SO_4_)^[^ ^61^, ^62^ ^]^ can be used in the electrochemical intercalation of graphene. The setup of the electrochemical cell and the preparation of the bulk graphite electrode can vary and need to be optimized depending on the intercalated ions in the electrolyte solution. In the case of ionic liquid electrolytes, the exfoliation of graphite can result in lower yields, smaller lateral sizes, and functionalization with ionic liquids.^[^ ^63^ ^]^ In contrast, acidic electrolytes can produce graphene with better quality and larger lateral sizes than ionic liquid electrolytes.^[^ ^60^ ^]^ However, acidic electrolytes can lead to higher levels of oxygen‐containing functional groups and a higher C/O ratio due to overoxidation in the acidic medium. Pervez et al. utilized various inorganic salts in aqueous electrolytes at neutral pH for the electrochemical intercalation of graphite.^[^ ^61^ ^]^ They found that sulfate salts, such as ammonium sulfate, are effective for exfoliating graphite due to the lower reduction potential of the sulfate anion (+0.20 V) compared to chloride, nitrate, and chlorate anions. According to their proposed mechanism, water molecules are reduced at the cathode when a bias voltage is applied, forming hydroxyl ions. These hydroxyl ions then nucleophilically attack the edge and grain boundary sites of the graphite, depolarizing and expanding the graphite layers. As a result, sulfate ions and water molecules intercalate between the layers. During the electrochemical process, the reduction of sulfate and the self‐oxidation of water produce SO_2_, O_2_, and other gases. These gases create forces that separate the weakly bonded stacked layers. It is noted that in this electrochemical process, graphite (mainly at the edges) may oxidize. According to their findings, they produced graphene with a large lateral size (up to 44 µm), few layers (≤3), a low oxidation degree (C/O ratio of 17.2), and excellent hole mobility (≈310 cm^2^V^1^s^1^).^[^ ^61^ ^]^ Zhao et al. used a similar electrochemical system to that of Pervez et al. to exfoliate graphite. They incorporated the stable organic radical TEMPO (2,2,6,6‐Tetramethylpiperidin‐1‐oxyl) into the electrolytes as a radical scavenger to prevent oxidative radical attack.^[^ ^62^ ^]^

##### Chemical Vapor Deposition

In the case of chemical vapor deposition (CVD)‐grown graphene, gaseous species such as methane and hydrogen are introduced into the reactor, passing through a hot zone where carbon radicals are formed. These carbon active species agglomerate to form thermodynamically stable (C_n_H_y_) species on the active sites of the metal surface, leading to graphene nuclei. After nucleation and growth, single or few‐layer epitaxial graphene films form on a metal substrate.^[^ ^65^ ^]^ The metal substrate acts as a catalyst to reduce the energy barrier of the reaction, and the deposition mechanism depends on the type of metal substrate used. The quality of the 2D film can be tuned by controlling various CVD growth parameters, including precursor concentration and feed rate, temperature, deposition time and rate, pressure, and the type of substrate.^[^ ^66^, ^67^ ^]^ While CVD‐grown films require expensive instrumentation and careful control of sophisticated parameters, the quality of 2D films produced by this method is significantly higher than those obtained via wet chemical synthesis or bottom–up techniques, and they can be directly integrated into various applications. The major disadvantage of this process is the need to transfer the CVD‐grown film from the catalytic metal substrate to a specific substrate, depending on the application. This requires a complex transfer process. A common transfer method involves coating the film with a polymer (such as PMMA), followed by using a metal etchant to remove the catalytic metal substrate. The polymer‐coated film is then transferred to the target substrate. Finally, the polymer is removed using a suitable organic solvent, leaving the 2D film on the desired substrate.^[^ ^68^ ^]^

##### Oxidation of Graphite for Graphene Oxide

Graphite flake is a naturally occurring mineral that contains many localized defects in its π‐structure, which are susceptible to oxidation.^[^ ^69^ ^]^ Graphene oxide (GO) can be obtained by the conversion of flake graphite to graphite oxide by oxidation, followed by exfoliation of graphite oxide to single or few‐layered GO. The exfoliation of GO from graphite oxide can be facilitated by sonication‐assisted aqueous or organic dispersion. The quality and functionalization of graphene oxide (GO) are strongly influenced by factors such as the oxidation method, the type of oxidizing agents, the nature of the graphite, the size of the graphite flakes, and the oxidation conditions. Controlling the oxidation process is the most important and challenging aspect of GO synthesis. GO obtained through less or partial oxidation contains fewer carboxyl and hydroxyl groups, and the reduced form of this GO exhibits high conductivity and fewer defects, resembling graphene‐like 2D materials. However, due to the lower degree of oxidation, the amount of exfoliated GO may be limited. On the other hand, complete oxidation of graphite can produce GO with a high quantity of hydroxyl and carboxylic functional groups, which is suitable for various post‐modifications and applications. However, the reduced form of fully oxidized GO may have lower conductivity, lower quality, and smaller flake sizes. Therefore, controlling the oxidation process and modifying existing oxidation methods may be necessary to fine‐tune the GO structure according to the specific application of graphene‐like 2D materials.

*Brodie's Method and Its Improvement*:

The oxidation of graphite was first observed by Brodie in 1859, who used powerful oxidants such as KClO_4_ and fuming nitric acid.^[^ ^70^ ^]^ Later in 1898, Staudenmaier refined Brodie's procedure by adding additional sulfuric acid with fuming nitric acid.^[^ ^71^ ^]^ This minor adjustment to the procedure greatly simplified the production of highly oxidized GO. In 1937, Hofmann further modified the procedure.^[^ ^71^ ^]^ However, these methods release ClO_2_ gas because of the use of potassium chlorate.

*Hummers’ Method and Its Improvement*:

Later, the oxidation process was refined by Hummers and Offeman, who utilized KMnO_4_ and H_2_SO_4_ with additional NaNO_3_,^[^ ^72^ ^]^ which was the most widely used oxidation method for graphite, known as the Hummers’ method. Although the oxidation process is faster compared to Brodie's procedure and does not release ClO_2_ gas, it still eliminates toxic gases such as NO_2_ and N_2_O_4_. Reduced graphene produced by thermal shocking of graphite oxide synthesized using the Hummers’ method exhibits higher heterogeneous electron transfer rates and lower overpotential compared to that produced from graphite oxide synthesized using the Staudenmaier or Hofmann's method (the modified Brodie procedure).^[^ ^73^ ^]^ GO synthesized by various methods greatly influences the adsorption of metal. A study of the removal of Pb(II), and Cd(II) ions from aqueous solution was found that GO synthesized from Hummers’ method are most efficient to remove these metal ions compared to GO produced by Staudenmaier, or Hofmann method which is probably due to the presence of more carboxyl groups in GO synthesized by Hummers’ method.^[^ ^74^ ^]^ Marcano et al. reported an improved oxidation method of graphite by using additional H_3_PO_4_ with H_2_SO_4_ and an excess amount of KMnO_4_.^[^ ^75^ ^]^ Usually, the thickness of a monolayer GO flake is ≈0.7–1 nm. The rGO flake prepared by Marcano was almost two layers.^[^ ^75^ ^]^ The electrical conductivity of rGO from Marcano's method was almost similar to that of Hummers’ and modified Hummers’ methods. Although no toxic NOx gases were produced in their oxidation method, an excess amount of H_2_SO_4_ (around five times higher than the Hummers’ method) and KMnO_4_ (2 times higher) were utilized, and the synthetic and purification procedure described in their report is quite complex.^[^ ^75^ ^]^ Although the procedure of Marcano's method and purification methods later were improved,^[^ ^76^, ^77^ ^]^ however, the use of excess sulfuric acid, KMNO_4_, and phosphoric acids makes it costly.

Chen et al. reported that KMnO_4_ and H_2_SO_4_ can effectively oxidize graphite without the need for NaNO_3_ or phosphoric acid,^[^ ^59^ ^]^ which were utilized in the Hummers’^[^ ^74^ ^]^ and Marcano's methods.^[^ ^75^ ^]^ By eliminating NaNO_3_ from the reaction, Chen et al. succeeded in eliminating the production of toxic gas. Moreover, Mn^2+^ can be easily removed through post‐treatment of the wastewater produced during the oxidation and purification process. Later, they improved this oxidation method further by adding water.^[^ ^59^ ^]^ Most importantly, depending on the addition of water and the reaction temperature, the functionalization of synthesized graphene oxide (GO) can be dominated by hydroxyl, epoxide, or carboxyl groups (Figure 4c). They prepared various graphene oxides using three times the weight of the oxidant KMnO_4_, corresponding to graphite's weight, with the addition of different amounts of water in 98% sulfuric acid, naming the samples GO‐n according to the amount of water added in mL. They found that the degree of oxidation and functionalization with epoxide and hydroxyl groups increases with increasing water addition.^[^ ^59^ ^]^ The color of graphite oxide becomes brighter due to the enhanced oxidation and functionalization. Enhanced oxidation and functionalization fragment the delocalized π‐conjugated structure of the graphene sheet into smaller domains, reducing the quality of the GO sheet when more than 6 mL of water is added. This water‐enhanced oxidation is due to strong oxidative radicals formed by Mn‐catalyzed O_3_ decomposition, generated from the oxidation of water with the Mn(VII) compound in the H_2_SO_4_ solution of KMnO_4_. They prepared GO‐0‐95 by adding 100 mL of water after 2 h of oxidation, then raising the temperature to 95 °C. This process resulted in carboxyl‐dominated functionalities and smaller GO sheets. GO defects can be either non‐permanent, removable upon reduction, or permanent, which cannot be fully healed by reduction. The defects in GO‐0‐95 arise from permanent vacancies, indicating that the reaction at 95 °C in the presence of remaining Mn (VII) and water severely destroyed the graphitic domains of the GO sheets (see the GO sheet size in Figure 1). Consequently, the conductivity of GO‐0‐95 was the lowest (see Figure 4c).

It is suspected that with slight modification, the simple modified Hummers method reported by Chen et al.^[^ ^59^ ^]^ could be effective for various applications due to its ability to control different functional groups.

#### 3.1.3 Applications of Graphene‐Based 2D Materials in Electrical and Electrochemical Sensors for Biomarker Detection

**Scheme 1:**
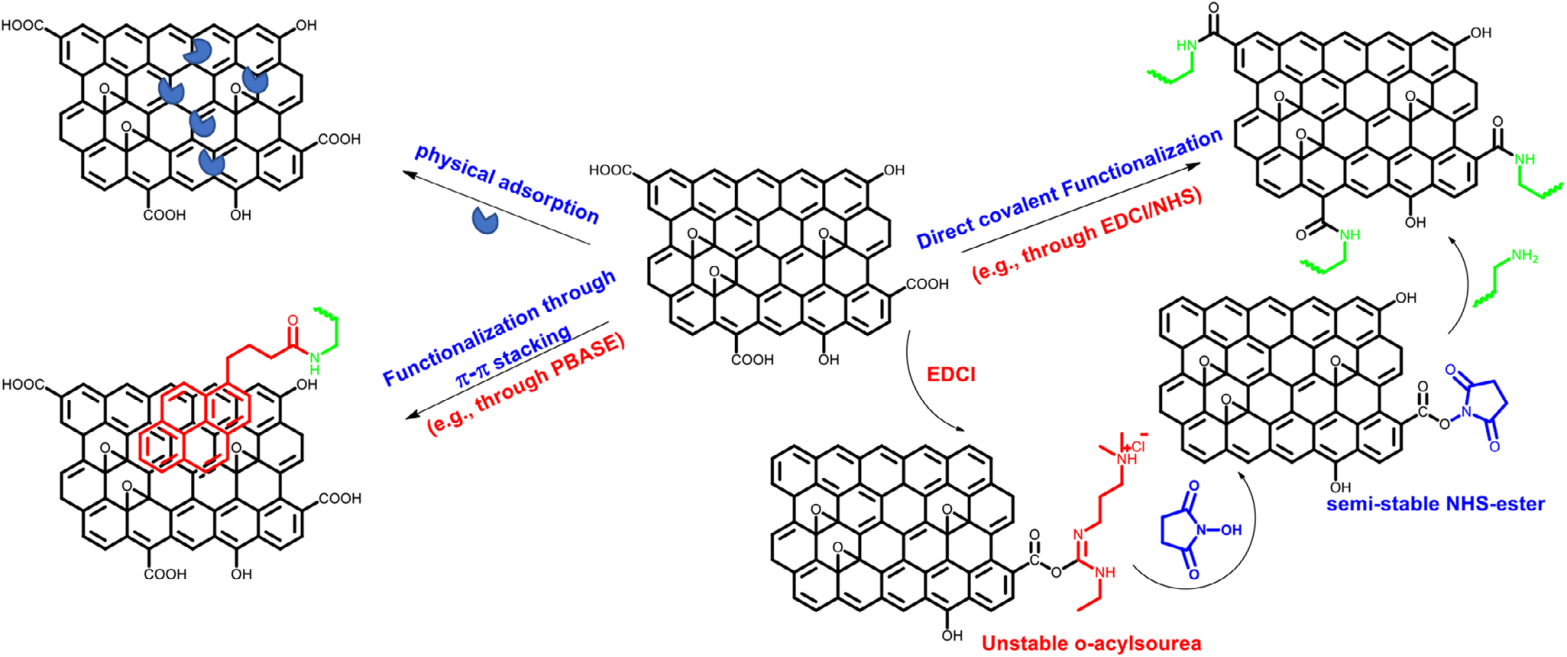
Three common approaches for immobilizing bioreceptors on 2D graphene/rGO‐based sensing platforms to selectively detect target biomarkers: physisorption, functionalization through π–π stacking using PBASE, and covalent linking via EDCl/NHS.

In biosensing, effective immobilization of bioreceptors (e.g., nucleic acids, antibodies, peptides, enzymes) is crucial for the selectivity, reproducibility, and sensitivity of the devices. Enzymes are often physically adsorbed onto the 2D nanosheet surface, while nucleic acids, antibodies, and peptides, which contain amine and other functional groups, can be covalently linked to a functionalized 2D nanosheet surface. Typically, bioreceptor immobilization on graphene‐based 2D nanosheet surfaces can be achieved in three ways: direct covalent functionalization, functionalization through π–π stacking, and physical adsorption (**Scheme** **1**).

##### Graphene

**Figure 5:**
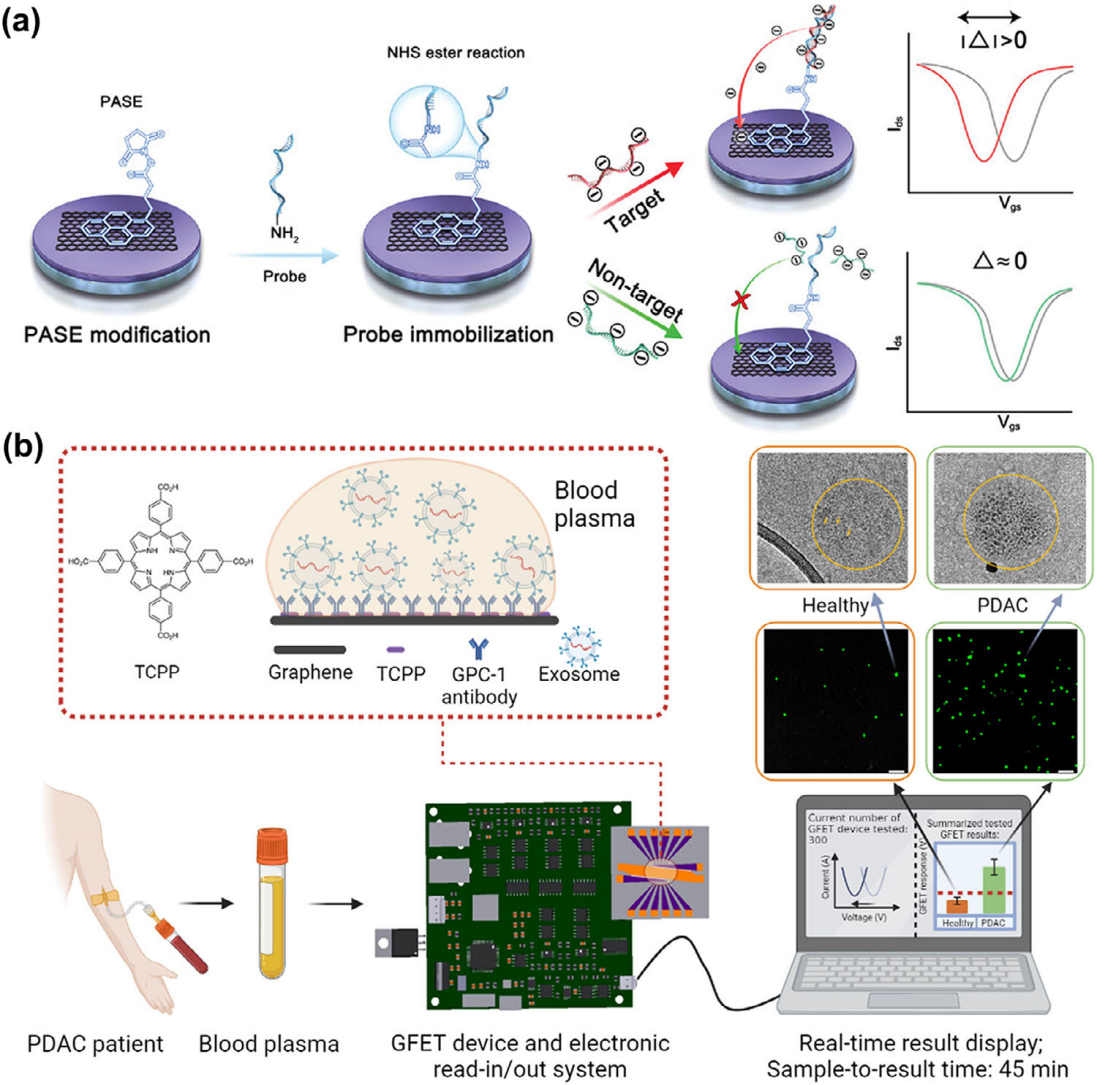
a) Schematic representation of functionalization and immobilization in the graphene sensing layer of a GFET using PBASE, along with the electrical response in the presence of target and non‐target species. Reproduced under terms of the CC‐BY license.^[^ ^46^ ^]^ Copyright 2024, Zhang et al. b) Schematic of a GFET for the real‐time detection of PDAC exosomes from patient plasma samples. Reproduced under terms of the CC‐BY 4.0 license.^[^ ^81^ ^]^ Copyright 2023, Yin et al.

Graphene is one of the most promising 2D materials for electrical and electrochemical biosensing due to its large surface area and high carrier mobility. Additionally, graphene offers ease in functionalizing aromatic biomolecules through π–π interactions. In the case of graphene, bioreceptors can be linked to its surface either through physical adsorption or by covalent linkage with functional groups of π–π stacked foreign species. For instance, the functionalization of bioreceptors (e.g., the antibody for carcinoembryonic antigen (CEA)) can be achieved using 1‐pyrenebutanoic acid succinimidyl ester (PBASE or PASE) on the graphene surface via π–π stacking (**Figure** **5**a), enabling the effective immobilization of the CEA antibody on the graphene surface through covalent linkage with PBASE.^[^ ^78^ ^]^ Tetrakis(4‐carboxyphenyl) porphyrin (TCPP) can also be used for π–π functionalization on the surface of 2D graphene nanosheets. The carboxylic acid groups of TCPP react with EDC/NHS to form a semi‐stable NHS ester, which is then converted to an amide bond through the reaction with the amine group of bioreceptors. Bovine serum albumin (BSA) is typically used after immobilization to block nonspecific binding sites on the 2D nanosheet surface. Hu et al. constructed an FET biosensor using CVD‐grown graphene for the detection of MicroRNA‐208a (miR‐208a), a cardiac‐specific miRNA for the early prediction of acute myocardial infarction, with a detection limit ranging from 0.01 to 1 pm and a LOD of 5.3 fm.^[^ ^79^ ^]^ Huang et al. achieved a limit of detection (LOD) of 100 am and a detection time of up to 30 min for miRNA detection by integrating CVD‐grown graphene‐based FET with exponential target recycling and hybridization chain reaction (TRHCR).^[^ ^80^ ^]^

The microfluidic graphene‐FET was recently fabricated for the significant detection of nucleic acids.^[^ ^46^ ^]^ Figure 5a illustrates the sensing mechanism of this device for controlling non‐target interference.^[^ ^46^ ^]^ Amine‐functionalized DNA probes are immobilized via covalent linkage with π–π stacked PBASE. The positive shift in the charge neutrality point (*V*_CNP_) after PBASE functionalization can be attributed to the p‐type doping effect of graphene due to charge transfer between the pyrene ring and graphene through π–π stacking. After probe DNA immobilization, a negative shift in *V*_CNP_ occurs due to the n‐type doping effect, caused by electron donation from the nucleotides. The detection of the target (e.g., 29‐mer RNA) is achieved selectively, as a more significant negative shift in *V*_CNP_ occurs upon hybridization of the DNA with RNA, causing greater electron donation from the double‐stranded DNA‐RNA hybrid. In contrast, non‐targets (e.g., a single‐nucleotide mutant of the target) cause only a slight shift, due to the lack of specific hybridization with the probe DNA (Figure 5a). An on‐chip point‐of‐care testing FET has also been successfully developed using a CVD‐grown graphene 2D film on Cu foil for the detection of pancreatic cancer exosomes in patient plasma (Figure 5b). This FET‐sensing platform can distinguish between plasma samples from healthy individuals and PDAC (pancreatic ductal adenocarcinoma) patients within 45 min.^[^ ^81^ ^]^

In most cases of electrical or electrochemical biosensors, CVD‐grown graphene is preferred because CVD produces high‐quality 2D materials (**Table** **1**). However, CVD is a complex synthesis method that demands precise control over numerous parameters, and its intricate transfer process further limits its suitability for cost‐effective, large‐scale production.

**Table 1:** Application of various 2D materials for electrical/electrochemical biosensing.

2D materials/Composite^a)^	Synthesis technique	Sensing Platform^b)^	Functionalization for bioreceptor^c)^	Bioreceptor^d)^	Target	LOD/Linear range	Year, Refs.
Graphene	CVD‐growth on Cu‐foil	Electrochemical, EIS	PBASE	anti‐CEA	CEA	0.23 ng mL^−1^/1.0–25.0 ng mL^−1^	2018, ^[^ ^78^ ^]^
Graphene	CVD‐growth on Cu‐foil	FET	PBASE	–	NT‐proBNP	1 pg mL^−1^/10 fg mL^−1^ to 100 pg mL^−1^	2024, ^[^ ^83^ ^]^
Graphene	CVD‐growth on Cu‐foil	FET	PBASE	–	miRNA‐208a	5.3 fm/0.01–1 pm	2024, ^[^ ^79^ ^]^
Graphene	CVD‐growth on Cu‐foil	FET	PBASE	–	22‐mer miRNA	100 am/100 am to 100 nm	2024, ^[^ ^80^ ^]^
Graphene	CVD‐growth on Cu‐foil	FET	PBASE	Anti‐CD63	Exosomes	0.1 µg mL^−1^	2024’ ^[^ ^318^ ^]^
rGO	GO:modified Hummar method, rGO: reduced with HI vapor at 80 °C for 3 h	Electrochemical, Resistance change	O_2_‐plasma treatment + EDC/NHS	Antibody	amyloid‐beta (Aβ) peptides	100 fg mL^−1^ to 1 ng mL^−1^	2017, ^[^ ^82^ ^]^
rGO	GO:modified Hummar method, rGO: reduced with hydrazine vapor at 80 °C for 2 h	FET	PBASE	–	NT‐proBNP	100 fg mL^−1^/10 fg mL^−1^ to 100 pg mL^−1^	2024, ^[^ ^83^ ^]^
2D WS_2_	Surfactant assisted liquid exfoliation	Electrochemical, DPV	No, Physisorption	–	miRNA‐4484	1.61 am/ 1 am–100 fm	2024, ^[^ ^123^ ^]^
2D MoS_2_ on Carbon electrode	surfactant‐assisted liquid phase exfoliation and cascade centrifugation	Electrochemical, CV	No	–	Dopamine, Reboflabin	10 µm/ 0 to 100 µm	2022, ^[^ ^122^ ^]^
2D MoS_2_	Mechanical exfoliation	FET	Al_2_O_3_‐dielectric materials deposition and then through APTES and GA	Anti‐PSA	PSA	100 fg mL^−1^	2017, ^[^ ^17^ ^]^
2D MoS_2_	Mechanical exfoliation	FET	No, Physisorption	Anti‐PSA	PSA	1 pg mL^−1^	2016, ^[^ ^110^ ^]^
2D MoS_2_	Mechanical exfoliation	FET	Al_2_O_3_‐dielectric materials and then Au nanoparticles deposition. DNA‐tetrahedron+ Biotin‐Streptavidin	DNA tetrahedron, B‐SA system, antibody	PSA	1 fg mL^−1^/ 1 fg mL^−1^ to 100 ng mL^−1^	2021, ^[^ ^114^ ^]^
2D MoS_2_	CVD	FET	Au nanoparticles	DNA probe	DNA (chromosome 21 or 13)	100 am/ 0 and 200 am	2019, ^[^ ^115^ ^]^
2D WS_2_	CVD	FET	Physisorption	DNA probe containing a poly‐C (C15)	complementary DNA	3 am/ 10^−16^ to 10^−9^ m	2022, ^[^ ^112^ ^]^
Ti_3_C_2_‐MXene on GCE	Etching from MAX phase	Electrochemical, CV	APTES	monoclonal anti‐ CEA	CEA	18 fg mL^−1^/ 0.0001–2000 ng mL^−1^	2018, ^[^ ^145^ ^]^
Au NPs/Ti_3_C_2_T_x_‐MXene on Au‐electrode	Etching from Ti_3_AlC_2_ MAX phase	Electrochemical, CV	Au─S chemical bonds	reduced thiol‐modified C‐DNA	microRNA‐155	0.35 fm/ 1.0 fm to 10 nm	2020, ^[^ ^146^ ^]^
MIP/ Ti_3_C_2_T_x_‐MXene on Ag electrode	Etching by LiF and HCl	Electrochemical, EIS	Ag‐4ATP‐GA	MIP Technique (CEA template)	CEA	9.41 ng mL^−1^ / 10 to 100 ng mL^−1^	2025 ^[^ ^151^ ^]^
*V*_2_C‐Mxene@Au NPs in SPCE	Etching from *V*_2_AlC MAX phase	Electrochemical, SWV	Au─S chemical bonds	Mucin1 Aptamer	Mucin1	3.45 fg mL^−1^/ 1.0 ‒ 500 pg mL^−1^	2025 ^[^ ^149^ ^]^
Ti_3_C_2_T_x_‐MXene	Etching from Ti_3_AlC_2_ MAX phase	Electrochemical, CV	PEI, GA	Anti‐Vit‐D	Vit‐D	1 pg mL^−1^/ 0.1 to 500 ng mL^−1^.	2025 ^[^ ^150^ ^]^
Ti_3_C_2_T_x_‐MXene/GO	Etching from Ti_3_AlC_2_ MAX phase	FET	EDC/NHS	Aptamer‐NH_2_	E. coli	3 CFU mL^−1^	2025 ^[^ ^154^ ^]^
Zr‐based MOF (521 MOF) on Au electrode	PVP‐assisted mild synthesis	Electrochemical, EIS	No functionalization, interaction of Zr‐ions with phosphate groups of aptamer strand	Mucin 1 aptamer sequence	Mucin 1	0.12 pg·ml^−1^ / 0.001 to 0.5 ng·ml^−1^	2017, ^[^ ^186^ ^]^
Co‐MOF on GCE	Triethyl amine assisted mild synthesis	Electrochemical, CV	–	–	H_2_O_2_	0.69 µm/ 0.5 µm to 832.5 µm	2020, ^[^ ^181^ ^]^
NiMn‐MOF on GCE	Hydrothermal process	Electrochemical, CA	–	–	Glucose	0.28 µm	2023 ^[^ ^182^ ^]^
Cu‐TCPP MOF@ Ag NPs on GCE	Benzoic acid assisted synthesis	Electrochemical, DPV	Various interactions, commonly π–π stacking	Aptamer OTA	**ochratoxin A** (OTA)	0.08 fg mL^−1^/ 0.10 fg mL^−1^ – 1 µg mL^−1^)	2021 ^[^ ^185^ ^]^
**CuZr‐MOF** On GCE	Hydrothermal conditions+ triethylamine assisted synthesis	Electrochemical, SWV	Various interactions, commonly π–π stacking	Aptamer	miR‐21	0.45 zm /1 zm to 1 pm.	2023 ^[^ ^187^ ^]^
AuNPs@COF on GCE	COF nanosheets: wet chemical process and ultrasonication Au NPS: in situ of Au NPs in COF nanosheets	Electrochemical, SWV	Various interactions	Exosome probe (activator DNA + exosome aptamer)	PD‐L1+ exosome	38 particles µL^−1^/ 1.2 × 10^2^ to 1.2 × 10^7^ particles µL^−1^	2022, ^[^ ^190^ ^]^
Au–Ag NPs‐COF on GCE	COF nanosheets: wet chemical process and ultrasonication Au and Ag NPS: in situ in COF nanosheets	Electrochemical, CV	–	–	H_2_O_2_	0.44 nm/ 2.0 nm–1.0 mm	2022, ^[^ ^191^ ^]^
COFs on SPCE	Modulating synthsis	Electrochemical, CV, DPV	Various interactions	MIP	glutathione	0.191 µm/ 1–1000 µm	2025 ^[^ ^189^ ^]^
ZnO (2D nanowalls) film supported on MoS_2_	ZnO: Electrodeposition MoS2: Solication‐assisted liquid exfoliation (DMF)	Electrochemical, DPV	No functionalization Electrostatic interaction of ZnO and ssDNA	ssDNA	DNA	6.6×10^−16 ^ m/ 1.0×10^−15^–1.0×10^−6^ m	2017, ^[^ ^206^ ^]^
MoO_3_‐WO_3_ on Au‐electrode	Atomic Layer Deposition	Electrochemical, CV	Electrostatic interaction	–	Dopamine	20 nm	2023 ^[^ ^202^ ^]^
Fe‐Cu‐LDH and rGO on PGE	Solvothermal process	Electrochemical, DPV	Various interaction	Anti‐PSA	PSA	100 fg mL^−1^–10 µg mL^−1^	2023 ^[^ ^213^ ^]^
NiCo LDH on SPCE	Solvothermal process	Electrochemical, CV	Various interaction	–	Lactate	0.53 mm	2021 ^[^ ^210^ ^]^
Ni–Cr LDH/GO on Au‐electtrode	Exfoliation‐reassembling	Electrochemical, CV, EIS, DPV	Covalent Interaction	Peptide	SDMA	0.1 ng mL^−1^/ 0–1 ng mL^−1^	2025 ^[^ ^214^ ^]^
Black phosphorous	Mechanical Exfoliation	FET	Al_2_O_3_ passivation layer, followed by Ni‐pattern (Au on Ni) then traped with CMC‐functionalized magnetic particles	Anti‐cortisol	Cortisol	1 am/ 1 am–1 µm	2024, ^[^ ^255^ ^]^
Black phosphorous on GCE	Liquid‐phase exfoliation in NMP	Electrochemical, DPV	–	–	Uric acid	0.33 µm/ 1–1000 µm	2023, ^[^ ^257^ ^]^
2D g‐C_3_N_4_/CuO	2D g‐C_3_N_4_/CuO: Pyrolysis	Electrochemical, CV and amperometric	No functionalization Electron transfer from CuO to Dopamine	–	Dopamine	1.1×10^−10^ m/ 2.00 × 10^−9^ to 7.11 × 10^−5^ mol L^−1^	2018, ^[^ ^224^ ^]^
h‐BN on GCE	Combustion, carbothermal reduction and nitridation methods	Electrochemical CV, DPV	–	–	Ascorbic Acid Dopamine Uric acid	3.77 µm,0.02 µm,0.15 µm/ 30–1000, 0.5–150 and 1–300 µm	2018, ^[^ ^239^ ^]^
Ge–H	Deintercalation of CaGe_2_ using HCl	Electrochecal. EIS	Physisorption	DNA‐probe	SNP	34pM/1 × 10^−12^ and 1 × 10^−8^ m	2021 ^[^ ^315^ ^]^
Ge–H	Deintercalation of CaGe_2_ using HCl	Electrochemical, CV	Physisorption	Glucose oxidase	Glucose	6.3 × 10^−6^ m	2021 ^[^ ^308^ ^]^
Ge–H	Deintercalation of CaGe_2_ using HCl	Electrochemical, EIS	Au NPs	ssDNA	cocaine drug	4.9 ± 0.1 am	2025 ^[^ ^316^ ^]^
Ge–H	Deintercalation of CaGe_2_ using HCl	Electrochemical, EIS	Physisorption	BSA‐KA‐AntiKA‐AntiIgG BSA‐QA‐AntiQA‐AntiIgG	KA QA	26.79 nm (5.07 ng mL^−1^) 68.11 nm (11.38 ng mL^−1^)	2024 ^[^ ^306^ ^]^

^a)^ GCE:glassy carbon electrode; PGE: pencil graphite electrode; SPCE: Screen‐printed carbon electrode;

^b)^ SWV: square wave voltammetry; DPV: differential pulse voltammetry; CV: cyclic voltammetry; EIS: electrochemical impedance spectroscopy; CA: Chronoamperometry; FET: field effect transistor;

^c)^ PBASE = 1‐ pyrenebutanoic acid succinimidyl ester, EDC = 1‐ethyl‐3‐[3‐dimethylaminopropyl]‐carbodiimide, NHS = N‐hydroxysuccinimide, APTES = 3‐aminoprpyl)triethoxysilane, biotin–streptavidin = B‐SA;

^d)^ Prostate cancer antigen = PSA, carcinoembryonic antigen = CEA, SDMA = Symmetric dimethylarginine.

The lack of a band gap and its chemical inertness reduce graphene's competitiveness in semiconductor and sensor technologies. Its limited processability in nontoxic aqueous solvents also makes graphene less competitive for biosensing devices.

##### Reduced Graphene Oxide

Reduced graphene oxide (rGO), a chemically modified form of graphene, is more processable and contains hydrophilic oxygen‐containing functional groups (e.g., ─COOH), which can be converted into semi‐stable NHS, Sulfo‐NHS, or HOBt esters using EDC/NHS, Sulfo‐NHS, or HOBt (NHS = N‐hydroxysuccinimide, HOBt = N‐hydroxybenzotriazole). Bioreceptors containing amine functionalities can then be directly immobilized on the rGO/GO surface through covalent linkage by forming amide bonds. This improves the selectivity and sensitivity of the device due to more effective immobilization. It has been found that O_2_‐plasma treatment of rGO can enhance surface reactivity for biomolecular interactions and improve the sensitivity of rGO‐based biosensing devices for the detection of amyloid‐beta (Aβ) peptides, a key pathological marker of Alzheimer's disease.^[^ ^82^ ^]^

Excess functional groups can reduce the conductivity of rGO sheets. Therefore, controlling the functionality of rGO during GO synthesis and its reduction is crucial for biosensing applications. It was recently reported that rGO‐based FETs exhibit better performance (higher dynamic range and lower limit of detection) than CVD‐grown graphene FETs for the detection of NT‐proBNP, which is the standard biomarker for heart failure.^[^ ^83^ ^]^ This improved performance is attributed to the rough surface of rGO and the presence of a bandgap. Therefore, rGO‐based electrical and electrochemical biosensors may be more promising than graphene‐based biosensors due to their inexpensive and scalable wet chemical synthesis, ease of modification, and the ability to tailor functional groups for effective bioreceptor immobilization.

### 3.2 Transition Metal Dichalcogenides (TMDs)

TMDs are a class of materials with the empirical formula MX_2_, where M represents a transition metal from groups 4–10, and X represents a chalcogen atom (e.g., S, Se, Te). TMDs with metal elements from groups 4–7 are usually layered in structure, while those from groups 8–10 are typically non‐layered. In this review, the discussion focuses on group‐6 TMDs (such as MoS_2_, MoSe_2_, MoTe_2_, WS_2_, and WSe_2_) due to their layered structures and relevance to biosensing applications. Group‐6 TMDs are found in nature^[^ ^84^ ^]^ and can also be produced through synthetic methods.^[^ ^85^ ^]^ In the layered structure, each layer is composed of an atomic layer of transition metal sandwiched between two atomic layers of chalcogen atoms, bound by strong covalent bonds. These layers are stacked together by weak van der Waals interactions to form bulk TMD crystals. The metal coordination in TMDs can be either trigonal prismatic or octahedral, depending on the arrangement of metal and chalcogen atoms.^[^ ^27^, ^28^ ^]^ Group‐6 metals (e.g., Mo, W) mostly favor trigonal prismatic coordination.

Unlike graphite, bulk TMDs exhibit different types of polymorphs and stacking polytypes due to the presence of three layers of atoms (X–M–X) in an individual MX_2_ monolayer. The most common polymorphs found in TMDs are 1T, 2H, and 3R, where the digit represents the number of X–M–X units in the crystalline unit cell, and the letter indicates the type of symmetry (T for tetragonal, H for hexagonal, and R for rhombohedral). In the case of single‐ or few layered 2D group‐6 TMDs, typically, two types of polymorphs, 1T (such as, 1T MoS_2_, octahedral metal coordination, D_3_d point group)^[^ ^86^ ^]^ or 1T’ (such as, 1T’ MoS_2_, distorted octahedral coordination)^[^ ^87^ ^]^ and 2H (such as 2H MoS_2_, trigonal prismatic coordination, D_3_h point group), are found.

#### 3.2.1 Importance of TMDs as 2D materials

In the TMD layer, no dangling bonds are present; as a result, the layer of TMDs exhibits excellent stability under atmospheric conditions. Due to their layered structures, TMDs from group 6 elements (such as MoS_2_) exhibit anisotropy similar to that of graphite. Specifically, their electrical, thermal, and mechanical properties are stronger in the in‐plane direction compared to the out‐of‐plane direction, owing to the strong bonding network within the layers and the weak van der Waals interactions between the stacked layers.

**Figure 6:**
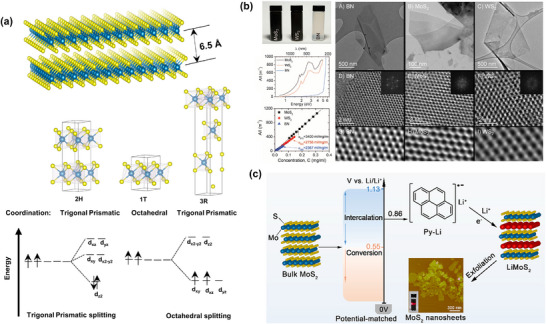
a) Structure of MoS_2_: three types of polymorphisms, and two different metal coordination with d‐orbital splitting. b) Photograph, absorption spectra, Lambert–Beer plots, and TEM analysis of liquid‐phase exfoliated MoS_2_, WS_2_, and h‐BN in organic solvents (NMP for MoS_2_ and WS_2_, and IPA for h‐BN). (b): Reproduced with permission.^[^ ^100^ ^]^ Copyright 2011, the American Association for the Advancement of Science (AAAS). c) Pyrene‐lithium‐assisted intercalation‐exfoliation technique. Reproduced with permission.^[^ ^101^ ^]^ Copyright 2022, ACS.

The electronic properties of TMDs can vary depending on the ligand‐field splitting of the d‐electrons of the transition metal, which is related to the coordination of the transition metal in TMDs. For instance, 1T MoS_2_ and WS_2_ are metallic due to the partial filling of degenerate d‐orbitals in octahedral coordination. On the other hand, 2H MoS_2_ and WS_2_ are semiconducting due to the complete filling of the d_z2​_ orbital caused by the splitting in trigonal prismatic coordination (**Figure** **6**a).^[^ ^27^, ^28^ ^]^ It should be noted that 1T MoS_2_ or WS_2_ is metastable and can be converted to 2H MoS_2_ by aging under ambient conditions, especially in the case of thin films produced by restacking single layers of MoS_2_ on a glass substrate,^[^ ^88^ ^]^ or by annealing at 300 °C.^[^ ^89^ ^]^ However, the stabilization of 1T MoS_2_ can be enhanced by various processes, such as doping with metals (e.g., Ru, V)^[^ ^90^, ^91^ ^]^ and functionalization.^[^ ^84^ ^]^ Due to its metallic characteristics, high surface area, and excellent conductivity, the 1T phase of MoS_2_ exhibits outstanding catalytic activity toward the hydrogen evolution reaction.^[^ ^92^, ^93^ ^]^

Single or few‐layered group 6 MX_2_ (such as MoX_2_ and WX_2_) has gained significant attention because of its unique physical and chemical characteristics compared to their bulk counterparts, primarily due to the confinement effect.^[^ ^94^, ^95^ ^]^ These materials typically appear as sheets, most commonly nanosheets. The thickness of an exfoliated monolayer of MX_2_ is ≈6–7 Å.^[^ ^96^ ^]^ The high surface area due to the planar structure, uncoordinated surface atoms, excellent mechanical and environmental stability, and solution processability make them suitable for producing transparent thin films, analogous to graphene‐like applications. Unlike graphene, which is a zero‐bandgap semimetal, single‐layer MoX_2_ possesses a direct intrinsic bandgap. Efforts to open a bandgap in graphene often lead to reduced carrier mobility and increased fabrication complexity.^[^ ^97^, ^98^ ^]^ This direct bandgap is an important feature for enabling efficient switching behavior in FETs and makes MoX_2_ a potential material for future electronic and optoelectronic devices. The indirect bandgap (≈1.3 eV)^[^ ^99^ ^]^ of semiconducting MoS_2_ transforms into a direct intrinsic bandgap (≈1.8 eV)^[^ ^21^ ^]^ in its monolayer form, making it suitable for constructing interband tunnel FETs,^[^ ^29^, ^30^ ^]^ which offer lower power consumption compared to conventional transistors. Due to the direct bandgap of 2D group‐VI MX_2_ materials, photoluminescence from single‐layer MX_2_ enables a wide range of optoelectronic applications.^[^ ^89^ ^]^ Although the mobility of single‐layer MoS_2_ is relatively low (≈0.5–3 cm^2^ V^−1^ s^−1^), studies have shown that placing a high‐κ dielectric layer, such as hafnium oxide, above the single‐layer MoS_2_ can enhance its mobility as high as 200 cm^2^ V^−1^ s^−1^,^[^ ^96^ ^]^ comparable to that of graphene nanoribbons.

#### 3.2.2 Current Synthetic Methods and Challenges in the Large‐Scale Production of 2D MoS_2_/WS_2_

##### Mechanical Exfoliation

By using suitable substrates with a strong affinity for adhering to 2D crystals (such as through covalent‐like quasi‐bonding (CLQB) between the substrate and the 2D layers), the top layer of a bulk layered crystal can be removed or separated without compromising its properties or structure. Noble metals, such as gold, can serve as substrates for CLQB with 2D crystals because their Fermi level remains in a partially filled band with mostly s‐electrons, which do not affect the electronic structure of the 2D crystals. Huang et al. reported the mechanical exfoliation of various TMDs on Au‐film substrates using Scotch tape.^[^ ^57^ ^]^ They also claimed that some layered materials, such as FeSe, PtTe_2_, and PdTe_2_, which cannot be exfoliated by other methods, can be successfully exfoliated using this Au‐supported mechanical exfoliation method.

##### Liquid‐Phase Exfoliation

Exfoliation of bulk layered materials by common organic and inorganic solvents using the aid of mechanical stirring and ultrasonication may result in single or few‐layered nanosheets because of weak van der Waals interaction among stacked layers. This liquid phase exfoliation can apply to most layered materials with weak van der Waals interactions. However, the efficiency of this simple top–down exfoliation depends on the number of factors. The most successful solvents are those that can minimize the energy of exfoliation by optimizing the balance of solvent–solvent, solvent–solute, and solute–solute binding energies.^[^ ^100^ ^]^ Coleman et al. achieved dispersion concentrations of up to 0.3 mg mL^−1^ for MoS_2_ and 0.15 mg mL^−1^ for WS_2_ in NMP by optimizing the dispersion procedure, including sonication and centrifugation time and conditions.^[^ ^100^ ^]^ Importantly, the exfoliation process using NMP solvent did not distort the hexagonal structure of MoS_2_ and WS_2_ (Figure 6b). However, when depositing the MoS_2_ exfoliated with this solvent onto a SiO_2_ substrate by spraying, some multilayers and clusters may form alongside the thin layer, likely due to aggregation during deposition.^[^ ^100^ ^]^ Since the boiling point of NMP is very high, the deposition process can be performed at ambient temperature. However, a certain amount of NMP may remain on the dry film. Zhou et al. utilized a mixed solvent, ethanol/water, to disperse and exfoliate TMDs.^[^ ^102^ ^]^ It is noted that neither water nor ethanol alone can disperse the TMDs in significant amounts. However, the combination of ethanol and water can disperse a reasonable amount of TMDs. Zhou et al. managed to disperse MoS_2_ and WS_2_ with concentrations up to 0.018 and 0.032 mg L^−1^, respectively, using an optimum mixed solvent ratio. However, the dispersion concentration was quite low compared to a single organic solvent, NMP.^[^ ^100^ ^]^ These exfoliated nanomaterials possess 2D characteristics, forming few‐layered sheets with no distortion of the hexagonal structure.^[^ ^102^ ^]^ Caution should be taken when ultrasound treatment is used; excessively high power may damage the exfoliated nanosheet size. Although exfoliation of layered materials by dispersion in common organic solvents through ultrasonication is a simple and cost‐effective approach, the yield of single‐ or few‐layered nanosheets is not high.

##### Chemical Intercalation

Group 6 TMDs, such as MoS_2_ and WS_2_, can typically be intercalated with Li^+^ using organolithium reagents (e.g., n‐butyl lithium in hexane)^[^ ^30^, ^103^, ^104^ ^]^ to form Li_x_MX_n_ reduced phases with expanded lattices, which are then exfoliated through a hydration process, usually with the aid of ultrasonication. During this process, hydrogen gas is evolved, which helps to separate the stacked layers into mono‐ or few‐layered TMDs. The exothermic reaction between Li_x_MX_n_ and water can produce a corrosive alkaline medium, and prolonged exposure can damage the exfoliated sheets. Lithium intercalation in 2H MX_n_ transforms it into a metastable 1T phase, altering its semiconducting properties.^[^ ^103^ ^]^ However, annealing at high temperatures or using IR laser‐assisted radiation^[^ ^103^ ^]^ can revert the 1T or (1T’) phase to the semiconducting 2H phase. Without sonication, the lithium intercalation process with n‐BuLi can be time‐consuming and require high temperatures.^[^ ^104^, ^105^ ^]^ Fan et al. reduced the intercalation time (≈1.5 h) by optimizing the reaction with ultrasonication, which speeds up the rate of Li^+^ intercalation through acoustic cavitation.^[^ ^103^ ^]^ It is important to note that the entire lithium intercalation process must be conducted under dry and inert conditions. Low yield of single or few‐layered TMDs can be obtained by incomplete lithium intercalation. In contrast, excess lithium intercalation may cause to decomposition of TMDs to metal nanoparticles and lithium sulfide.^[^ ^101^ ^]^ Therefore, the control of lithium intercalation can be challenging. Zheng et al. used alkali metal adducts, such as sodium, potassium, or lithium naphthalene, instead of organolithium compounds to exfoliate MoS_2_.^[^ ^106^ ^]^ In the metal naphthalene adduct, the metal donates an electron to the aromatic system, forming a radical anion with strong reducing properties. Initially, they expanded the MoS_2_ layers using a hydrazine solution under hydrothermal conditions (heating in an autoclave at 130 °C for 48 h). During this intercalation, N_2_H_4_ is oxidized to N_2_H_5_^+^. The intercalated N_2_H_5_^+^ is thermally unstable and can produce N_2_, H_2_, and NH_3_ gases upon heating, which causes the MoS_2_ to expand ≈100 times compared to its initial size. In the second step of intercalation, various alkali metal naphthalene adducts were used. After exfoliation, they found that sodium naphthalene provided the best quality MoS_2_ monolayer flakes. However, the two‐step procedure for MoS_2_ intercalation and purification is quite cumbersome. Additionally, the preliminary expansion in a closed autoclave at high temperatures may pose safety concerns due to the production of various gases, particularly hydrogen and ammonia, in a confined system. According to the voltage profile, cyclic voltammetry, and in situ XRD during the electrochemical lithiation process, Zhu et al. found that the lithium ion intercalation reaction in MoS_2_ (forming a lamellar LiMoS_2_, 1T phase) occurs at +1.13 V, whereas the decomposition of intercalated LiMoS_2_ to Mo and Li_2_S occurs at +0.55 V.^[^ ^101^ ^]^ They claim that the direct use of lithium naphthalene adducts may cause the decomposition of bulk MoS_2_ into Mo and Li_2_S due to the over‐reduction power of lithium naphthalene adducts (redox potential +0.38 V, which is lower than the decomposition reaction potential).^[^ ^101^ ^]^ Therefore, a pre‐expansion process may be required before using lithium naphthalene adducts for intercalation. Instead of lithium naphthalene adducts, Zhu et al. found pyrene lithium for the safe interaction with MoS_2_ because of its higher positive redox potential (+0.86 V, higher than the decomposition potential), which prevents the decomposition of the bulk MoS_2_ (Figure 6c). The reduction ability of pyrene lithium is less than that of naphthalene lithium due to greater electron delocalization in the quaternary benzene ring of pyrene and a lower HOMO energy compared to naphthalene lithium. They succeeded in reducing the intercalation process to a single step and a short time (within 1 h), achieving a single‐layer MOS_2_ nanosheet yield of ≈80%. However, the lateral dimension of MoS_2_ nanosheets obtained by this procedure was reported to be ≈100–600 nm,^[^ ^101^ ^]^ which was smaller than the reported dimension (3–10 µm) from Zheng et al.’s method.^[^ ^106^ ^]^ Recently, Zhao et al. reported on 2D MoS_2_ exfoliation techniques using zero‐valent transition metal complex intercalants, such as dicobalt octacarbonyl (Co_2_(CO)_8_).^[^ ^107^ ^]^ This intercalant‐assisted exfoliation method can produce mixed‐phase (both 2H and 1T) 2D MoS_2_ nanosheets. However, the removal of intercalant materials requires treatment with hydrochloric acid

##### Electrochemical Intercalation

In the electrochemical lithiation process, layered Group‐6 TMDs bulk materials coated on copper act as the cathode, while lithium foil serves as the anode. A Li‐salt (typically LiPF_6_) dissolved in a carbonate solvent is used as an electrolyte. The electrochemical lithiation is performed using galvanostatic discharge at a low current (usually 0.05 mA). In the discharge process, Li⁺ ions are inserted into the interlayer spaces of layered materials, which weakens the van der Waals interactions. After the discharge process is complete, the intercalated compound (e.g., Li_x_MX_2)_ is removed, washed, and then exfoliated by ultrasonication in ethanol or water.^[^ ^108^ ^]^

The point should be noted that the layered TMDs bulk materials are usually deposited on copper foil using a binder (typically PVDF polymer) and conductive carbon black nanoparticles, and removing these materials can be a cumbersome process. Additionally, the amount of intercalated compounds depends on the surface area of the electrode. Conducting this electrochemical process also requires a battery test system and an Ar‐filled glove box, which complicates this type of exfoliation technique.

#### 3.2.3 Applications of 2D MoS_2_/WS_2_ in Electrical and Electrochemical Sensors for Biomarker Detection

Due to the direct bandgap of 2D TMDs (e.g., MoS_2_), they are preferred over graphene as channel layers in FETs, as they help reduce leakage current. Using MoS_2_ as the channel material and HfO_2_ as the dielectric, a high on/off current ratio above 1 × 10⁸ at room temperature has been reported.^[^ ^96^ ^]^ The absence or low density of dangling bonds on the MoS_2_ surface, along with its inherent n‐type conductivity, results in higher‐quality FETs with fewer scattering centers and lower flicker noise levels.^[^ ^109^ ^]^

##### Bioreceptor Immobilization via Physisorption

The presence of van der Waals affinity or hydrophobicity makes the MoS_2_ surface effective for the nonspecific binding of bioreceptors through physisorption. For instance, Yoo et al. reported an MoS_2_‐FET sensor with a LOD of 1 pg mL^−1^, where the anti‐PSA antibody was non‐specifically bound to MoS_2_ via physisorption.^[^ ^110^ ^]^ This is feasible due to the reasonable hydrophobicity of the MoS_2_ surface, which is comparable to that of Au nanoparticles,^[^ ^111^ ^]^ known for their ability to physisorb proteins and biomolecules. Bahri et al. reported a CVD‐grown WS_2_‐FET DNA biosensor with a LOD of 3 am, where a diblock DNA probe containing a (poly‐C) (C15) sequence was physisorbed onto the WS_2_ surface.^[^ ^112^ ^]^

##### Bioreceptor Immobilization via Coating a Thin Film of Dielectric Oxides or Au

Generally, 2D TMDs are chemically inert; therefore, functionalizing their surfaces for effective bioreceptor immobilization can be quite challenging. Unlike graphene‐ or rGO‐based sensors, covalent functionalization for immobilizing bioreceptors on MoS_2_‐based sensors is usually performed indirectly. Commonly, a thin film of dielectric material, such as HfO_2_^[^ ^113^ ^]^ or AlO_2,_^[^ ^17^ ^]^ is deposited atomically on top of the MoS_2_ channel layer in FET sensors. O_2_ plasma treatment can then be effective in generating hydroxyl groups on the thin films of high‐dielectric metal oxides.

**Figure 7:**
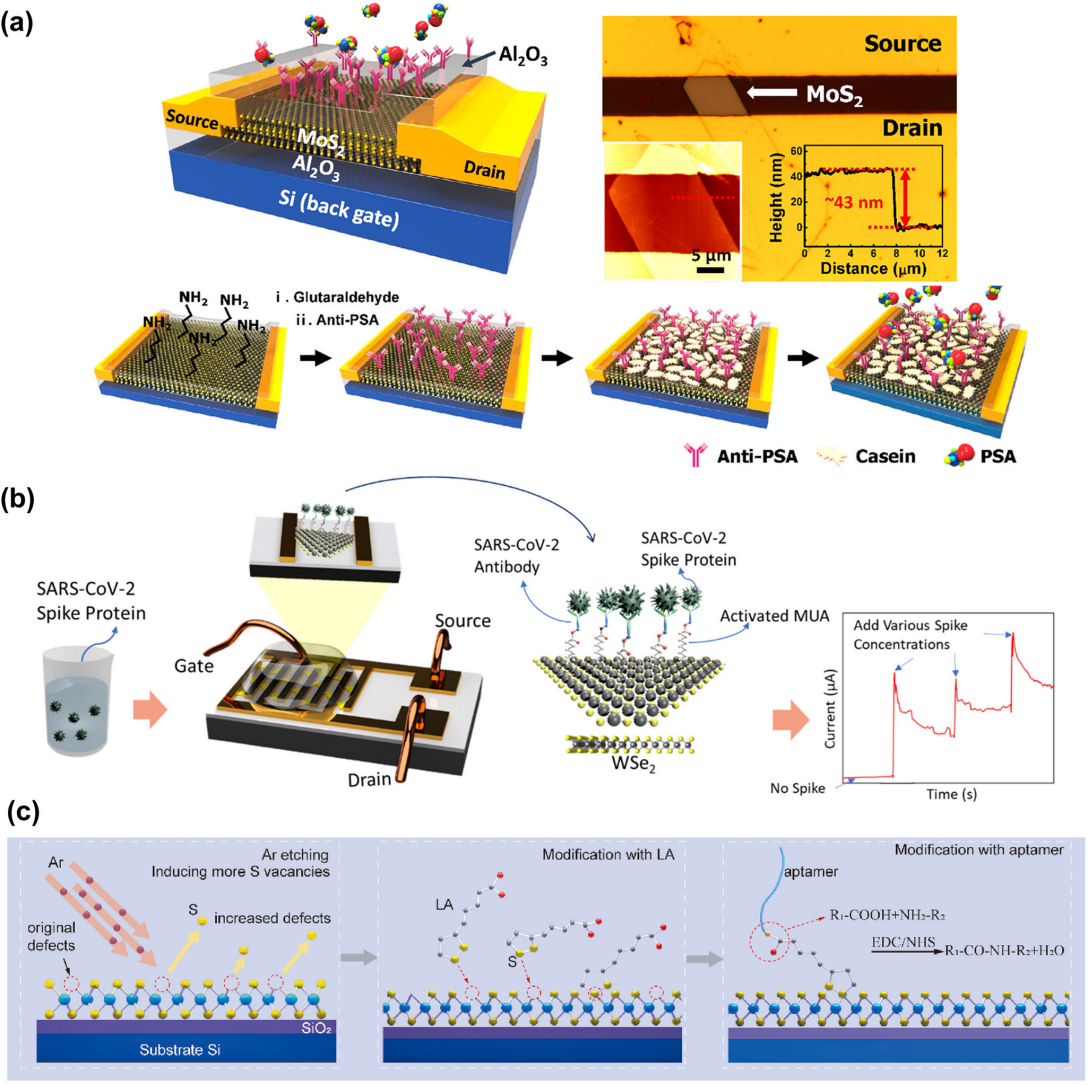
a) Ultrathin Al_2_O_3_ high‐k dielectric layer on an MoS_2_ channel for APTES functionalization. Reproduced with permission.^[^ ^17^ ^]^ Copyright 2017, ACS. b) Schematic representation of a WSe_2_‐based FET sensor for the detection of SARS‐CoV‐2, showing covalent functionalization of the antibody via adsorption of MUA and activation of its carboxyl groups using EDC/NHS coupling. Reproduced with permission.^[^ ^118^ ^]^ Copyright 2021, ACS. c) Schematic illustration of defect creation at sulfur vacancy sites in MoS_2_ by argon etching, followed by repair with lipoic acid (LA) for covalent functionalization of the aptamer through EDC/NHS coupling in a MoS_2_‐FET biosensor. Reproduced with permission.^[^ ^119^ ^]^ Copyright 2025, Elsevier.

Due to the presence of available hydroxyl groups on the surfaces of Al_2_O_3_ or HfO_2_, these surfaces can effectively couple with silane coupling agents (e.g., triethoxysilylpropylamine, APTES) to introduce amine groups. These amine groups can then react with glutaraldehyde (GA) to form aldehyde functional groups, which can covalently bond with the amine groups of a bioreceptor (**Figure** **7**a). After APTES functionalization on high‐k dielectrics in MoS_2_‐FETs, sulfo‐NHS‐biotin can also be covalently attached to the amine‐functionalized APTES, enabling selective detection of streptavidin protein through the well‐known streptavidin–biotin complex. Park et al. reported an MoS_2_‐based FET biosensor for the detection of prostate cancer antigen (PSA) with a limit of detection of 100 fg mL^−1^, where they functionalized aldehyde groups through APTES and GA on an Al_2_O_3_ dielectric film in the MoS_2_‐FET (Figure 7a).^[^ ^17^ ^]^ The anti‐PSA antibody then specifically bound through covalent bonds via the well‐known aldehyde–amine chemistry. Another functionalization technique, demonstrated by Zhang et al. in an MoS_2_‐FET sensor, was the thermal deposition of Au on an Al_2_O_3_ layer to facilitate the formation of a self‐assembled monolayer with DNA tetrahedrons. Streptavidin was used to anchor the DNA tetrahedra to biotin‐anti‐PSA. A blocking buffer (BB) containing 1% casein and BSA in PBS was used to prevent nonspecific binding.^[^ ^114^ ^]^ In the case of MoS_2_ FET biosensors, if bioreceptor functionalization is performed indirectly, by coating a thin dielectric layer or Au film over the MoS_2_ sensing layer, there may be a reduction in device sensitivity due to the increased distance between the target and the MoS_2_ surface. Liu et al. deposited Au nanoparticles on the MoS_2_ sensing layer to immobilize probe DNA through Au─S bond formation, enabling the specific detection of target DNA with a detection limit of 0.1 fm.^[^ ^115^ ^]^

Direct APTES functionalization on the MoS_2_ surface may be possible through O_2_ plasma treatment. Ryu et al. reported the functionalization of APTES on the MoS_2_ surface, which was then linked with biotin or GA for the detection of streptavidin or interleukin‐1β, respectively.^[^ ^116^ ^]^ Park et al. constructed nanopores in the MoS_2_ surface using BCP lithography for effective edge‐selective functionalization.^[^ ^117^ ^]^ The resulting thermodynamically unstable nanoring edges were oxidized by O_2_ plasma treatment and subsequently functionalized with APTES‐GA. As a result, a large number of aptamer–cortisol complexes could be selectively captured on the nanoring edges of the MoS_2_ nanopores through efficient aldehyde–amine chemistry. However, the creation of nanopores in MoS_2_ can be a complex and sophisticated process.

##### Bioreceptor Immobilization via Adsorption through Thiol‐Containing carboxylic Acids

During the synthesis or exfoliation of 2D TMDs, defects such as vacancies on chalcogen atoms may be generated. Theoretical studies reveal that sulfur vacancies in MoS_2_ or other TMDs can be repaired using thiol‐containing molecules.^[^ ^120^, ^121^ ^]^ Fathi‐Hafshejani et al. constructed a WSe_2_‐based FET for detecting SARS‐CoV‐2.^[^ ^118^ ^]^ To enhance the interaction between biomolecules and the WSe_2_ crystal, the 11‐mercaptoundecanoic acid (MUA) chemical linker was first attached to the WSe_2_ surface, likely through the SH‐terminated end of MUA binding to selenium vacancy sites on the WSe_2_ surface. A self‐assembled monolayer (SAM) of MUA can then form through chemisorption and physisorption. AFM images clearly show that the thickness increased after MUA attachment, probably due to the formation of the SAM on the WSe_2_ surface. A comparison of the density of states near the Fermi level between the MUA and the WSe_2_ system indicates that no charge transfer occurred between them. The COOH group of MUA can then be activated through EDC/NHS chemistry to form an N‐hydroxysuccinimide ester, which can interact with the amine groups of antibodies to form an amide bond (Figure 7b). Very recently, Wang et al. reported a MoS_2_‐based FET biosensor for cytokine detection, in which they functionalized MoS_2_ using a lipoic acid linker containing a 1,2‐dithiolane ring.^[^ ^119^ ^]^ To ensure efficient attachment of the lipoic acid linker, they first created defects on the MoS_2_ surface by argon etching to generate reactive sites on the MoS_2_ channel. The carboxylic acid group in the lipoic acid, attached to the defect site on the MoS_2_ surface, was then activated through EDC/NHS coupling to enable effective and covalent binding of the aptamer (Figure 7c).

These functionalization and immobilization techniques pave the way for constructing sensitive and selective group‐6 TMD‐based FET biosensors for various biomarker detections and disease monitoring.

2D TMDs (such as MoS_2_ and WS_2_) have also been successfully used to modify working electrodes for the electrochemical detection of dopamine, riboflavin,^[^ ^122^ ^]^ miRNA,^[^ ^123^ ^]^ and other analytes, highlighting their great potential in electrochemical biosensing.

### 3.3 Transition Metal Carbide, Nitride, or Carbonitride (MXene)

MXenes are transition metal carbides, nitrides, or carbonitrides with the general formula M_n+1_X_n_T_x_ (where n = 1–4). Typically, they consist of 2–5 atomic layers of a transition metal M (generally from Groups 3–6: Sc, Y, Ti, Zr, Hf, V, Nb, Ta, Cr, Mo, W) interleaved with X layers (carbon and/or nitrogen). Surface termination groups, T_x_ (e.g., ─OH, O, S, F, Cl), form on the outer basal plane of the transition metal M during the synthesis of MXenes from their parent MAX phase. The MAX phase is a ternary transition metal carbide, nitride, or carbonitride with the formula M_n+1_AX_n_, where A represents a metal layer from Group 13 or 14 (such as Al, Si, or Ga). Due to the variety of termination groups and transition metals, number of atomic layers (currently, 2–5) of transition metal, and different type of X layer (C, N, or CN), an infinite number of MXenes is possible, and over thirty types of MXene have already been produced (such as Ti_3_C_2_T_x_,^[^ ^124^ ^]^ Ti_2_CT_x_,^[^ ^125^ ^]^ Ti_3_CNT_x_,^[^ ^126^ ^]^ Hf_3_C_2_T_x_,^[^ ^127^ ^]^ Nb_2_CT_x_,^[^ ^128^ ^]^ *V*_2_CT_x_,^[^ ^129^ ^]^ Mo_2_CT_x_,^[^ ^130^ ^]^ and Ta_4_C_3_T_x_,^[^ ^131^ ^]^ and so on). Among these MXenes, Ti_3_C_2_T_x_ is the most studied MXene. The surface termination groups in MXenes can be single or mixed, depending on the synthetic methods used to derive MXenes from the parent MAX phase and their compositions.

#### 3.3.1 Importance of MXenes as 2D Materials

It is assumed that all MXenes exhibit metallic conductivity in the absence of surface termination groups because the free electrons in the transition metals act as carriers. Unlike metals, the termination groups in MXenes change the density of states (DOS) and shift the Fermi level, resulting in tunable electrical properties.^[^ ^19^, ^132^, ^133^ ^]^ The termination groups on MXenes significantly impact their properties, including hydrophilicity, environmental stability, and electrical, thermoelectric, thermal, optical, and mechanical properties. Based on DFT calculations, it was also predicted that ─OH and ─F terminations on Ti_3_C_2_ alter the semimetal characteristics of non‐terminated Ti_3_C_2_ to semiconducting characteristics, with a separation between the valence and conduction bands of ≈0.05–0.1 eV. Thus, tuning the electronic properties by changing the surface termination functional groups is possible, making MXene, like MoS_2_, suitable for electronic applications such as transistors.^[^ ^31^ ^]^ Theoretical studies predict that Cr_2_C‐based MXene may exhibit a ferromagnetism‐antiferromagnetism transition, caused by a half‐metal to insulator transition, due to surface functionalization with ─F, ─OH, ─H, and ─Cl groups, and this transition can be controlled by altering the surface functional groups.^[^ ^134^ ^]^ Vacuum annealing or thermal treatment can lead to the desorption or complete removal of termination groups.^[^ ^132^, ^135^ ^]^ During annealing or thermal treatment, oxygen terminations are more stable than fluoride. For example, heating Ti_3_C_2_T_x_ above 550 °C results in the desorption of ─F termination groups, with oxygen taking their place. At 750 °C, the ─F groups are completely removed. Hart et al. found that partial removal of surface termination groups (─OH, ─F, and ═O) through vacuum annealing can increase conductivity.^[^ ^132^ ^]^ The de‐functionalization rate of Mo_2_TiC_2_T_x_ was found to be higher than that of Ti‐based MXenes.^[^ ^132^ ^]^

The intercalants used for delaminating MXene layers also affect their electrical properties.^[^ ^132^ ^]^ Large organic cations like TBA^+^ (tetrabutylammonium ion) decrease conductivity more than smaller alkali cations (such as Li^+^), as large ions increase the interlayer gaps of MXene flakes. The removal of TBA^+^ and water intercalants via vacuum annealing alters the semiconducting properties of MXenes to metallic.

Flake size and defects also affect the electrical and optical properties of MXenes.^[^ ^136^ ^]^ Increasing the flake size improves conductivity and optical absorbance, while smaller flake sizes enhance electrolyte access due to an increased number of active sites. By improving the quality of Ti_3_C_2_ MXene from a more organized Ti_3_AlC_2_‐MAX phase using excess Al, high electrical conductivity of up to 20000 S cm^−1^ with improved oxidation stability has been reported for solution‐processed MXene 2D films.^[^ ^137^ ^]^ The free‐standing film of Ti_3_C_2_T_x_ MXene produced using blade‐coating techniques exhibits outstanding tensile strength, up to 560 MPa, which is greater than that of aluminum foil.^[^ ^138^ ^]^ Based on a theoretical study, it was found that nitride‐based MXenes may exhibit better conductivity, structural, and elastic stability than carbide‐based MXenes due to their lower lattice constants and smaller monolayer thickness.^[^ ^139^ ^]^

Thus, in contrast to other 2D materials, which are typically semiconductors, semimetals, insulators, or dielectrics, MXenes offer 2D nanosheets with metallic electrical conductivity. Furthermore, their electrical, thermal, optical, and many other important properties can be tuned by modifying surface terminations through various synthetic methods and post‐modification processes. Their hydrophilicity and solution processability make them uniquely promising 2D materials for various applications, including electronics, optoelectronics, energy storage, catalysis, and sensing. Furthermore, the use of MXenes with other 2D materials and conductive polymers to fabricate solution‐processable heterostructures and devices makes them highly attractive as future 2D materials. The hydrophilicity, electrochemical activity in various analytes, and rich surface chemistry of MXene make it highly suited for biosensing.^[^ ^19^ ^]^

#### 3.3.2 Synthesis of MXene from Parent MAX Phase by Selective Etching and Challenges

**Figure 8:**
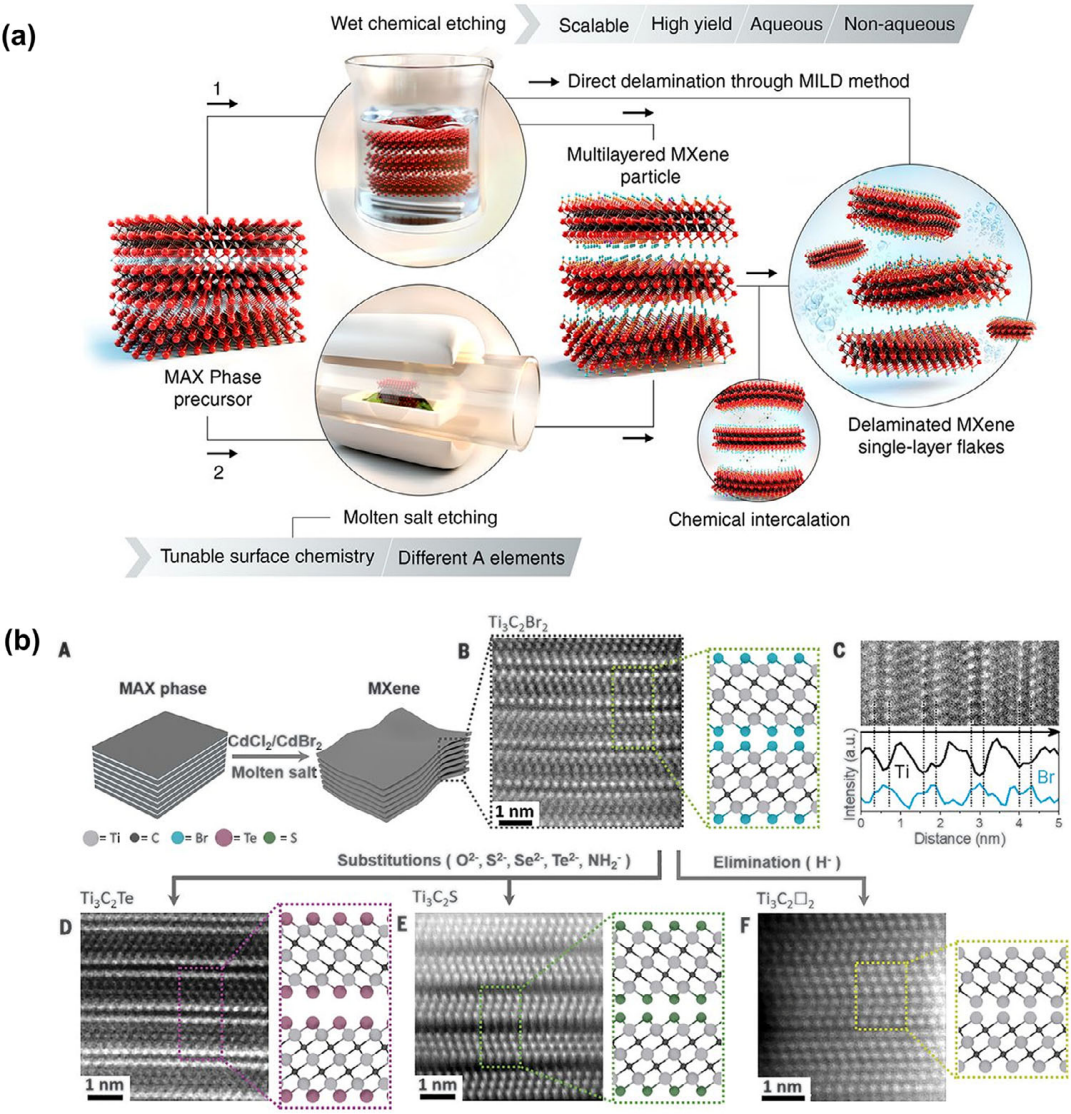
a) Comparison of wet chemical and molten salt etching to produce MXene from a MAX precursor. Reproduced under terms of the CC‐BY license.^[^ ^19^ ^]^ Copyright 2021, VahidMohammadi et al. b) The production of Br‐terminated Ti_3_C_2_Br_2_ MXene by molten salt etching and post‐modification of Br surface termination to various substituents. Various MXenes with atomic‐resolution high‐angle annular dark‐field (HAADF) images are shown. The square box in Ti_3_C_2_□_2_ indicates the vacancy formed by the removal of Br termination groups. Reproduced under the terms of the CC‐BY license.^[^ ^140^ ^]^ Copyright 2020, Kamysbayev et al.

In the production of MXene (M_n+1_X_n_T_x_), the A layer (Al, Si, or Ga) is usually selectively removed from the MAX (M_n+1_AX_n_) phase using a suitable etchant to obtain weakly stacked MX layers. Further separation of the MX layers through sonication or with the help of intercalants can produce single‐layer flakes (**Figure** **8**a).

It is important to note that, despite the comparatively weaker metallic bonding between the M and A atomic layers (compared to the ionic and/or covalent bonds between the M and X atomic layers in MAX phase crystals), mechanical exfoliation to produce MXene by removing the A layer is not possible. However, selective electrochemical or chemical etching of the A layer in the MAX phase, which is a kinetically and thermodynamically favorable reaction that dissolves the reaction products, can successfully produce MXene. In 2011, the first MXene (Ti_3_C_2_T_x_) was reported from Ti_3_AlC_2_ by selective etching of the Al layer using an aqueous HF solution.^[^ ^31^ ^]^ In this process, the surface termination groups can be hydroxyl, oxygen, and fluoride.^[^ ^31^, ^141^ ^]^ Alhabeb et al. provided updated guidelines for MXene synthesis, either through in situ HF formation within the reaction vessel or by using a direct aqueous HF solution.^[^ ^142^ ^]^ According to their study, a 5% HF solution can completely etch the Al layers and effectively produce Ti_3_C_2_T_x_ MXene. High‐quality MXenes with larger flake sizes can be achieved by ex situ HF formation using a mixture of HCl and LiF, a method they referred to as the “mild” version of MXene synthesis. Although this procedure cannot completely etch all the Al layers, it results in a mixture of MXene and MAX phase powders, which can be easily separated by centrifugation at different rotational speeds. Another advantage of this method is that, due to the presence of Li⁺ ions in the reaction system, single‐ or few‐layered MXene nanosheets in aqueous dispersion can be directly obtained via exfoliation during purification with deionized water.

Aqueous acid solution etching of the MAX phase results in oxidation and hydrolysis, leading to mixed surface termination groups in MXenes. The etching of the MAX phase using molten salt can avoid unwanted oxidation and hydrolysis, resulting in MXenes with a single termination group (Figure 8b). For instance, etching the MAX phase with molten salts such as ZnCl_2_ or CuCl_2_ at 750 °C can produce MXenes with a single Cl‐ surface termination group.^[^ ^143^, ^144^ ^]^ Kamysbayev et al. reported the production of MXene with Br surface termination groups by etching the MAX phase using molten CdBr_2_.^[^ ^140^ ^]^ These bromine‐terminated groups can be further modified to oxygen, sulfur, selenium, tellurium, and NH groups (Figure 8b). Different surface‐terminated MXenes can be produced as stable colloidal solutions of single‐layer flakes through Li intercalation, followed by sonication in the polar organic solvent N‐methylformamide. It is noted that this type of molten salt etching process requires high temperatures above 600 °C and an Ar‐filled glove box. In contrast, wet chemical etching is more convenient, can be used with both aqueous and nonaqueous solvents, and does not require sophisticated experimental setups. Further research into the wet chemical etching process, focusing on identifying suitable etchants and intercalants for MXenes with desirable surface termination groups, could be a promising avenue.

#### 3.3.3 Applications of MXenes in Electrical and Electrochemical Sensors for Biomarker Detection

MXene can be distinct from other 2D materials due to its metallic, high electrical conductivity, hydrophilicity, ease of functionalization, and good ion‐intercalation ability. These properties make MXene a promising candidate for electrical and electrochemical biosensing applications.

**Figure 9:**
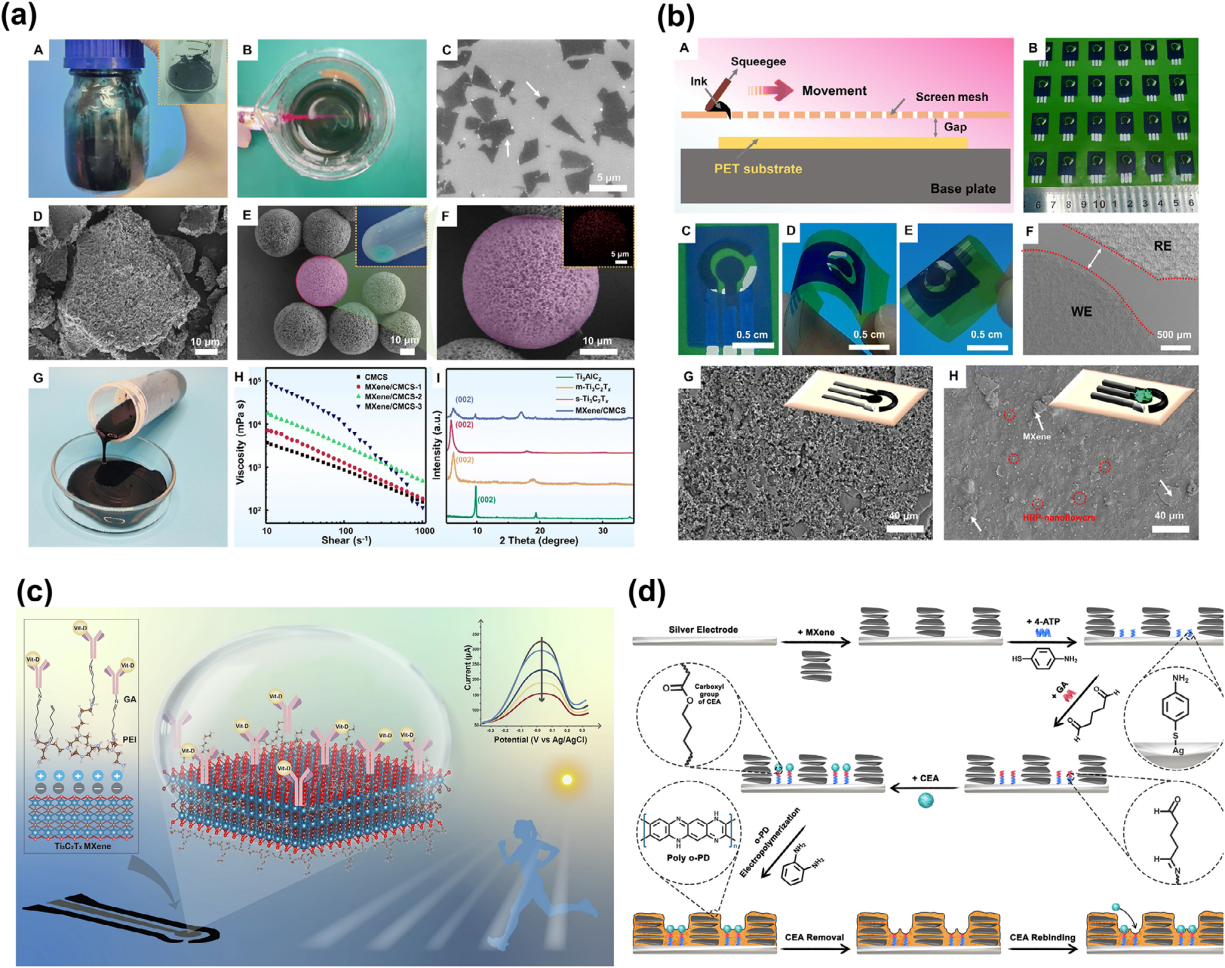
a) Characterization of single‐ and multilayered MXene and HRP nanoflowers after self‐assembly, along with screen‐printable MXene/CMC ink. b) Schematic of the screen‐printing fabrication process and large‐scale preparation of a three‐electrode electrochemical sensor, with corresponding SEM images. Reproduced with permission.^[^ ^147^ ^]^ Copyright 2024, RSC. c) Schematic illustration of an electrochemical sensor based on amine‐functionalized MXene with PEI for the detection of vitamin D. Reproduced under the terms of CC‐BY‐NC‐ND license.^[^ ^150^ ^]^ Copyright 2025, Barman et al. d) Schematic representation of the electrochemical MIP biosensor fabrication and bioreceptor immobilization technique for the detection of CEA. Reproduced with permission.^[^ ^151^ ^]^ Copyright 2025, ACS.

Kumar et al. developed an APTES‐functionalized mono‐ or few‐layered MXene (Ti_3_C_2_)‐based electrochemical biosensor for the detection of carcinoembryonic antigen (CEA), achieving a linear detection range of 0.0001–2000 ng mL^−1^ and a sensitivity of 37.9 µA ng^−1^ mL cm^−2^.^[^ ^145^ ^]^ They also found that [Ru(NH_3_)_6_]^3+^ is more effective as a redox probe compared to [Fe(CN)_6_]^3─/4−^, as the oxidation and reduction of [Ru(NH_3_)_6_]^3+^ occur at lower potential windows, which helps minimize the oxidation of the MXene layer at higher potentials. Yang et al. developed an Au nanoparticles (NPs)/Ti_3_C_2_ MXene‐based electrochemical sensor for the detection of miRNA‐155, achieving a detection limit of 0.35 fm (S/N = 3) with a linear range of 1.0 fm to 10 nm.^[^ ^146^ ^]^ This performance is attributed to the selective detection of the target through Au–S‐linked, reduced thiol‐modified C‐DNA bioreceptors. Recently, an electrochemical biosensor was fabricated using screen‐printing of an aqueous MXene ink prepared by combining multilayer MXene with carboxymethyl cellulose (CMC).^[^ ^147^ ^]^ Horseradish peroxidase (HRP) nanoflowers, catalytic enzymes with flower‐like nanostructures formed through self‐assembly, were then immobilized on the electrode surface for the effective detection of hydrogen peroxide (**Figure** **9**a,b). This screen‐printing fabrication approach offers a cost‐effective and scalable method for the production of MXene‐based electrochemical biosensors. There is a report on a *V*_2_CT*_x_*‐MXene‐based electrochemical sensor fabricated on a carbon electrode, using Nafion as an immobilizing agent for MXene and glutaraldehyde for the effective binding of protein A. This enables the further immobilization of anti‐SARS‐CoV‐2 antibodies for the detection of the SARS‐CoV‐2 spike protein.^[^ ^148^ ^]^ It should be noted that Nafion can physically interact, likely through hydrogen bonding, with the hydroxyl‐terminal groups on the MXene surface, resulting in a uniform film coating. Additionally, Nafion can interact with glutaraldehyde (GA) through physical interactions such as hydrogen bonding and entrapment within its ionic clusters. However, Nafion cannot form covalent bonds with GA. The MUC1 biomarker was effectively detected electrochemically using a system based on *V*_2_C‐MXene nanosheets and Au nanoparticles (Au NPs). In this approach, the MUC1‐specific aptamer was modified with thiol groups and immobilized on a carbon electrode (SPCE) modified with *V*_2_C‐MXene@Au NPs with Cd^2+^ ions. This electrochemical biosensor exhibited excellent performance with high selectivity for MUC1 detection within a range of 1.0–500 pg mL^−1^, achieving a LOD of 3.45 fg mL^−1^.^[^ ^149^ ^]^ Since Ti_3_C_2_T_x_ MXenes produced by HF etching contain negatively charged functional groups such as ─F, ─OH, and ─O, their aqueous dispersions can electrostatically interact with positively charged ions. Barman et al. functionalized amine groups onto the MXene surface using polyethyleneimine (PEI), taking advantage of the electrostatic interaction between the positively charged functionalities of PEI and the negatively charged MXene surface. GA was then used to cross‐link the amine groups, introducing aldehyde groups. Subsequently, Anti‐Vitamin D (Anti‐Vit‐D) was immobilized through covalent linkage via aldehyde–amine interactions (Figure 9c). Using this effective covalent immobilization technique, their electrochemical sensor was able to detect vitamin D deficiency with a remarkable LOD of **1 pg mL^−1^** and a wide detection range from **0.1 to 500 ng mL^−1^**.^[^ ^150^ ^]^

Instead of using natural receptors, a layer of artificial receptors called molecularly imprinted polymers (MIPs) can be created to form a receptor template for electrochemical biosensing. MXene was incorporated into the MIP layer to enhance the sensitivity of the sensor. First, the Ag electrode was modified with MXene, followed by the formation of self‐assembled monolayers (SAMs) using 4‐aminothiophenol (4‐ATP).^[^ ^151^ ^]^ The 4‐ATP interacts with the Ag electrode through S–Ag bonds within the nano/microcavities formed between the MXene nanoparticles, providing confined spaces for interaction between the solution and the analyte. The amine group of 4‐ATP is then functionalized with GA to enable an aldehyde–amine reaction with CEA. Subsequently, molecularly imprinted polymers (MIPs) are created by the electropolymerization of ortho‐phenylenediamine (o‐PD), facilitating CEA template removal, improving imprinting efficiency, and enhancing sensor stability (Figure 9d).

An MXene‐based FET electrical biosensor for dopamine sensing has also been reported.^[^ ^152^ ^]^ There was a report of an extended‐gate FET aptasensor based on MXene. In this case, the functional groups on the MXene surface were activated by EDC/NHS coupling, without any additional modification to introduce carboxylic acid groups.^[^ ^153^ ^]^ It is important to note that as‐synthesized MXenes typically do not possess carboxylic acid termination groups. Furthermore, if the existing epoxy or hydroxyl surface terminations cannot be converted into a carboxyl‐containing linker, EDC/NHS coupling would not be an effective option. The GO and Ti_3_C_2_T_x_ MXene composite layer was also used as the channel material in an FET sensor. Due to the introduction of GO, covalent functionalization through EDC/NHS coupling became feasible. As a result, an amine‐functionalized aptamer could be directly linked via covalent bonds, enabling effective immobilization. This provided the sensor with improved selectivity, reproducibility, and a detection limit of 3 CFU mL^−1^ for *E. coli*.^[^ ^154^ ^]^ Recently, Li et al. fabricated a hydrogel‐gated FET sensor by combining CVD‐grown graphene and MXene.^[^ ^155^ ^]^ They functionalized MXene with APTES, taking advantage of the available ─OH groups on the MXene surface, which can covalently bond with the silanol groups in APTES. However, they did not explain how the SARS‐CoV‐2 antibody binds to the amine groups of APTES.

MXene‐based electrochemical and electrical FET biosensors are still in the early stages of development. The effective immobilization of bioreceptors on the MXene 2D sensing layer is crucial for the sensitivity and overall performance of the device. After summarizing the recent developments, it can be assumed that amine functionalization through APTES or PEI would be an effective strategy for bioreceptor immobilization on the MXene surface. Another effective approach could be Au NPs or a thin film of Au on the MXene surface that immobilizes the bioreceptor through an Au–S covalent bond. Molten salt etching of the MAX phase can produce MXene with Br‐terminated surfaces, which can be further modified to amine or other functionalities to effectively immobilize the bioreceptor for the selective and effective detection of the target biomarker. Further research is required to optimize the functionalization of MXene 2D layers for bioreceptor immobilization, with in‐depth analysis needed to explore various MXenes with different surface functionalities. Challenges such as corrosion from using HF, high temperatures, reagent costs, and other limitations associated with the selective removal of the metal layer from the parent MAX‐phase to obtain MXene need to be addressed.

### 3.4 2D Metal or Covalent Organic Frameworks

Metal–organic frameworks (MOFs) are crystalline porous materials constructed from networks of metal ions or clusters (metal nodes) coordinated with organic ligands. These frameworks can be 1D, 2D, or 3D, depending on the combination of metal ions and organic ligands used. As a result, the physical and chemical properties of MOFs can vary based on the type of metal ions, organic ligands, their combinations, structures, and morphologies. Like MOFs, another type of crystalline porous material is known as covalent organic frameworks (COFs).

#### 3.4.1 Importance of 2D Metal or Covalent Organic Frameworks

Compared to 3D MOFs, 2D MOFs have attracted significant attention due to their thin, layered structures with atomic or molecular thickness.^[^ ^32^, ^33^ ^]^ They are also more flexible and easier to process due to their sheet‐like nature, offering a high surface area and moderate conductivity. 2D MOFs may have lower thermal and chemical stability, as well as reduced porosity, compared to 3D MOFs. Despite these challenges, their tunable structures, large surface area, ultrahigh porosity, and abundant active sites make 2D MOFs promising for electrocatalysis and biosensing. Similar to 2D MOFs, 2D COFs also hold promise as electrocatalysts and in biosensing due to their porous structure, tailored functionalities, and chemical and thermal stability.

#### 3.4.2 Synthesis of MOFs and COFs and challenges

The controlled synthesis of 2D MOFs is more challenging than that of bulk MOFs.

The synthetic methods of 2D MOFs have been broadly discussed in several review papers.^[^ ^158^, ^159^, ^160^, ^161^, ^162^ ^]^ The synthesis of 2D MOF can be done by two general approaches: the top–down approach, applicable for layered MOF, and the bottom–up approach, chemically grown from starting precursors

##### Top–Down Approaches

Like graphite and other layered materials, layered MOFs can be exfoliated using various top–down approaches, such as mechanical and liquid‐phase exfoliation, by applying different types of forces.

Layered MOFs possess strong coordination bonds within the layers and weak van der Waals or hydrogen bonds between the layers. Physical forces such as sonication in liquid dispersion, shear force, and ball‐milling can break these weak interactions and exfoliate the material into 2D nanosheets.

A class of pillared‐layered 3D MOFs, M_2_(bdc)(dabco), where M = Zn, Co, or Ni, H_2_bdc = 1,4‐benzenedicarboxylic acid, and the pillar ligand dabco = 1,4‐diazabicyclo‐[2.2.2]octane, can be exfoliated into 2D MOFs by breaking the interlayer bonds through the exchange of pillar ligands with capping solvent guest molecules, assisted by sonication. Sonication also helps prevent the reorganization of the 2D MOF layers.^[^ ^163^ ^]^

 Another liquid‐phase exfoliation was carried out on bulk layered MOFs (such as ELM‐12, Cu(bpy)_2_(OTf)_2_, where bpy = 4,4′‐bipyridine and OTf = trifluoromethanesulfonate, and Zn_2_(bim)_4_, where bim = benzimidazole) in an acetone dispersion using shear force generated by a commercial blender.^[^ ^164^ ^]^ Usually, liquid‐phase exfoliation through ultrasonication and shear force not only breaks the weak interlayer bonds but can also damage the 2D nanosheets. Another challenge is the selection of a suitable solvent; sometimes, a mixed solvent system is more effective, as it needs to both exfoliate and stabilize the 2D nanosheets.

A softer physical exfoliation method, such as ball‐milling combined with low‐power ultrasonication, can reduce this damage. Peng et al. exfoliated Zn_2_(benzimidazole)_3_(OH)(H_2_O) nanosheets using a soft physical method involving ball‐milling and ultrasonication in a mixed solvent.^[^ ^165^ ^]^ Jiang et al. exfoliated Ni_3_(HITP)_2_ (HITP = 2,3,6,7,10,11‐hexaaminotriphenylene) nanosheets using a soft physical method involving ball‐milling and ultrasonication.^[^ ^166^ ^]^

Top–down approaches that apply external physical forces are simple and convenient, but these forces can damage the crystallinity of the materials, resulting in nonuniform flake sizes and limited exfoliation. However, the selection of a suitable solvent and the optimization of sonication or other external forces can improve exfoliation while reducing structural defects.

##### Bottom–Up Techniques

2D MOFs can also be synthesized using various bottom–up techniques, including interfacial synthesis, three‐layer synthesis, modulated synthesis, templating synthesis, hydrothermal, and wet chemical synthesis.

**Figure 10:**
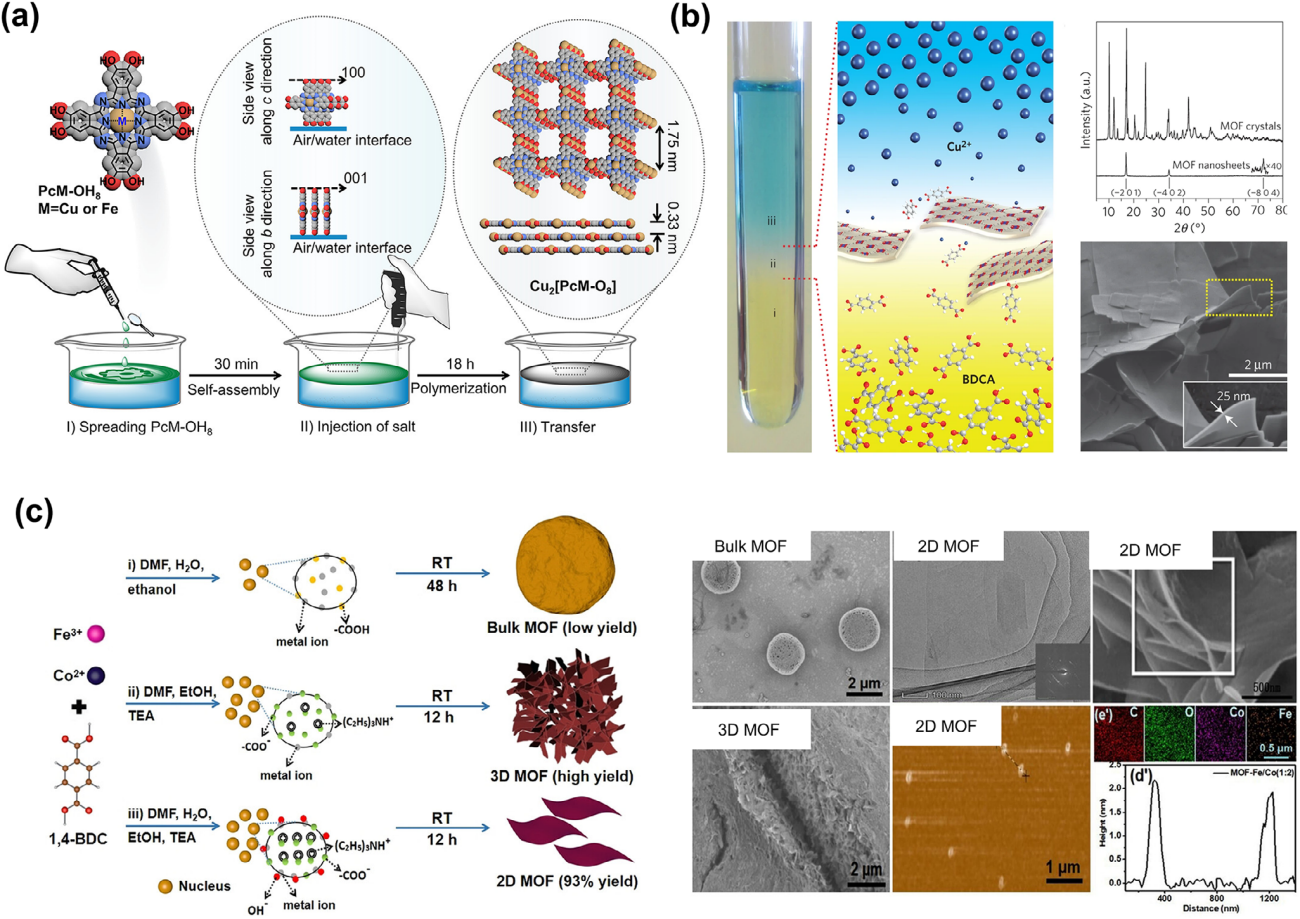
Growth of MOF nanosheets via various bottom–up synthesis methods. a) Schematic representation of Cu_2_[PcCu–O_8_] 2D MOF film grown at the water/air interface. Reproduced with permission.^[^ ^156^ ^]^ Copyright 2021, ACS. b) Synthesis of CuBDC nanosheets with lateral sizes of 0.5–4 µm and thicknesses of 5–25 nm using a three‐layer synthesis technique. Reproduced with permission.^[^ ^33^ ^]^ Copyright 2014, Springer Nature Limited. c) A simple synthetic process for obtaining 2D bimetal MOF nanosheets in the presence of water and TEA, along with SEM, TEM, and AFM images of the synthesized bulk, 3D, and 2D MOF. Reproduced with permission.^[^ ^157^ ^]^ Copyright 2021, Wiley.

*Interfacial synthesis*:

In interfacial synthesis, MOF nanosheets are grown at interfaces such as liquid/liquid, liquid/air, or liquid/solid by reacting metal nodes with ligands. In the case of liquid/liquid interfacial synthesis, metal ions and ligands are dissolved in two different immiscible liquids (e.g., water and ethyl acetate/dichloromethane). For example, Cu–BHT (BHT = benzenehexathiol) MOF nanosheets^[^ ^167^ ^]^ and 2D copper Cu‐TCPP MOF (TCPP = tetrakis(4‐carboxyphenyl)porphyrin)^[^ ^168^ ^]^ were synthesized using this method. In liquid/air interfacial synthesis, a small amount of an organic solvent containing the ligand is sprayed onto the water surface. The evaporation of the volatile solvent creates a water/air interface. Metal salts are carefully added to the water layer. The reaction between the metal ions and ligands at the water/air interface forms a 2D MOF film (**Figure** **10**a). This method allows for better control over the nucleation and growth kinetics of MOFs. Cu_2_[PcCu–O8] 2D MOF film (PcCu–O8 = (2,3,9,10,16,17,23,24‐octahydroxyphthalocyaninato)copper) with a thickness of ≈20 to ≈600 nm, a room temperature conductivity of ≈5.6 × 10^−4^ S cm^−1^, and a Hall mobility of ≈4.4 cm^2^ V^−1^ s^−1^ was reported using water/air interfacial synthesis.^[^ ^156^ ^]^ A thin film of {Fe(py)_2_[Pt(CN)_4_]} (py, pyridine) 2D MOF was reported by the liquid/solid interfacial synthesis.^[^ ^169^ ^]^

*Three‐layer synthesis*:

In three‐layer synthesis, two miscible solvents (e.g., DMF and acetonitrile) with different densities are used along with a third buffer layer. The solvent with higher density (such as DMF) forms the bottom layer, where the metal ions are dissolved, while the solvent with lower density (such as acetonitrile) forms the top layer, where the ligands are dissolved. The buffer layer (such as a mixture of equal amounts of acetonitrile and DMF) remains in the middle. In this layer, metal ions from the bottom layer and ligands from the top layer diffuse and form thin MOF nanosheets. These MOF nanosheets precipitate in the middle layer, which can control the overgrowth of nanosheets (Figure 10b). Examples include the synthesis of CuBDC (BDC = 1,4‐benzenedicarboxylate) nanosheets with lateral sizes of 0.5–4 µm and thicknesses of 5–25 nm.^[^ ^33^ ^]^ ZnBDC, CoBDC,^[^ ^170^ ^]^ and 2D MOFs such as Cu(1,4‐NDC) and Cu(2,6‐NDC) (where NDC = naphthalenedicarboxylate) can also be synthesized using this method.^[^ ^32^ ^]^

*Modulated synthesis*:

Anisotropic growth of MOFs with various morphologies^[^ ^171^ ^]^ (e.g., nanocubes, nanorods, and nanosheets) can be achieved using small molecules, known as modulators (such as acetic acid and pyridine), which have functional groups similar to the organic linkers. These modulators competitively coordinate with the metal nodes to control the growth of the MOF crystals. For instance, [Cu_2_(BDC)_2_(BPY)]_n_ nanosheets were reported to be synthesized via modulated synthesis using acetic acid as the modulator.^[^ ^172^ ^]^ The size of the nanosheets depends on the concentration of the modulator

Sonication can generate high temperature and pressure in closed, airtight conditions. MOF nanosheets can also be produced from precursor solutions using sonication. The synthesis of Ni‐Co‐MOF nanosheets has been reported via sonication, where a colloidal solution of Co^2^⁺, Ni^2^⁺, BDC ligand, and triethylamine in a water/DMF/ethanol mixture was sonicated for 8 h under airtight conditions.^[^ ^22^ ^]^ This method is convenient and straightforward for MOF synthesis. However, controlling the morphology and nanosheet size can be challenging.

*Templating Synthesis*:

Recently, Huang et al. demonstrated a templating technique to synthesize various 2D conductive (c)‐MOFs through the transformation of insulating MOFs into c‐MOFs.^[^ ^173^ ^]^

The resulting nanostructured 2D c‐MOFs possess electrical conductivity and a larger surface area than bulk 3D MOFs. Some 3D MOFs (such as CuBDC, ZIF‐8, and HKUST‐1) can be unstable in acidic solutions (pH 5.0) or under atmospheric conditions due to weak metal–oxygen or metal–nitrogen bonds, whereas 2D c‐MOFs can remain stable in acidic solutions (as low as pH 3.0). Therefore, 3D MOFs can act as sacrificial templates for the synthesis of 2D c‐MOFs, facilitated by the thermodynamic driving force. Recently, a free‐standing quasi‐2D MOF/GO heterostructured film was developed using acoustotemplating, based on the concept that acoustomicrofluidic techniques are capable of aligning GO sheets horizontally into stacked planar layers and crystallizing quasi‐2D (q‐2D) copper‐based MOF structures (such as Cu‐BTC).^[^ ^174^ ^]^ This process templates the MOF within the interstitial spaces between the aligned GO layers. The available metal ions in the quasi‐2D sword‐like MOF crystals can coordinate with the oxygen functionalities in GO, providing an efficient heterostructured film.

*Wet Chemical Synthesis*:

Ge et al. synthesized bimetal MOF‐Fe/Co nanosheets with high yield (above 90%) by a simple wet chemical synthetic procedure at room temperature.^[^ ^157^ ^]^ They found that in the presence of a small amount of water and triethylamine (TEA), 2D MOFs could be grown and stabilized. TEA deprotonated the COOH groups of 1,4‐BDC (1,4‐benzene dicarboxylic acid) and produced a small amount of hydroxide ions in water, which stabilized the edges of the 2D MOF and prevented aggregation into 3D MOFs (Figure 10c). The resulting bimetal MOF showed excellent electrocatalytic ability for the oxygen evolution reaction (OER). It is suspected that this type of bilayer 2D MOF with electrocatalytic ability could be suitable for electrochemical biosensors.

##### 2D COFs

Like 2D MOFs, 2D COFs can also be synthesized by similar bottom–up approaches such as interfacial synthesis, hydrothermal synthesis, and templated synthesis. Lamellar or layered COFs, having weak interactions either through van der Waals forces, π–π stacking, or hydrogen bonding, can also be exfoliated by top–down approaches. Some recent review articles and book chapters cover the synthesis of COFs exclusively.^[^ ^175^, ^176^, ^177^, ^178^ ^]^ Readers are encouraged to explore these resources for detailed synthetic processes. Recently, Cusin et al. reported the preparation of micrometre‐thick, oriented 2D COF films via a kinetically controlled pathway.^[^ ^179^ ^]^ They synthesized imine‐linked 2D COFs by coating a 3D covalent adaptable network (CAN) intermediate onto a Si substrate, which spontaneously aligns to relieve tensile stresses caused by solvent evaporation. A subsequent lift‐off process transforms the 3D‐oriented polymer network into a free‐standing, porous 2D COF film. The transformation from an amorphous to a crystalline film occurs under solvothermal annealing.

#### 3.4.3 Applications of 2D MOFs/COFs in Electrical and Electrochemical Sensors for Biomarker Detection

##### 2D MOFs

2D MOFs with high electrocatalytic activity and good compatibility with electrodes can efficiently catalyze the redox reactions of analytes such as glucose, hydrogen peroxide, dopamine, ascorbic acid, and uric acid, which are linked to various health conditions.^[^ ^180^ ^]^ MOFs often exhibit size‐selective adsorption due to their porous structures, where the pores act as molecular sieves. This allows smaller molecules or atoms to pass through and be adsorbed, while larger ones are excluded, making MOFs effective for size‐based selectivity in applications like gas separation and sensing. Liu et al. found that a 2D Co‐MOF‐based non‐enzymatic hydrogen peroxide electrochemical sensor outperforms Ni or NiCo‐MOF‐based devices, with a low detection limit of 0.69 µm and a fast sensing response in alkaline solution.^[^ ^181^ ^]^ This superior performance is attributed to the better conductivity and catalytic ability of Co‐MOF in peroxide redox reactions. The synthesis of the Co‐MOF nanosheet was also a straightforward process, involving the dissolution of the 1,4‐BDC (1,4‐benzenedicarboxylic acid) ligand and cobalt salt in the presence of triethylamine, followed by ultrasonication. Zr‐based MOFs are promising candidates for biomedical applications due to their low toxicity and high stability. Mao et al. prepared an electrochemical glucose biosensor using ultrathin bimetallic NiMn‐MOF nanosheets synthesized via a hydrothermal process. This biosensor demonstrated a detection limit of 0.28 µm (S/N = 3). The presence of heterogeneous metals and the ultrathin morphology contributed to the enhanced catalytic activity in glucose oxidation.^[^ ^182^ ^]^ Ultrathin 2D conductive (c)‐MOF nanosheets (CuHHTP), synthesized via a surfactant‐assisted solution method, were combined with Au nanoparticles (Au NPs) to construct a nonenzymatic electrochemical sensor for the detection of H_2_O_2_, taking advantage of their synergistic electrocatalytic activity for sensitive H_2_O_2_ detection.^[^ ^183^ ^]^

DNA can be anchored onto MOF surfaces through π–π stacking, metal coordination, hydrogen bonding, or van der Waals interactions. Hu et al. constructed a CoNi bimetallic MOF‐based electrochemical sensor for the detection of miRNA‐126.^[^ ^184^ ^]^ The complementary DNA (cDNA) can be strongly anchored on the MOF surface and within its pores through various interactions, including hydrogen bonding, π–π stacking, and metal coordination. As a result, an amplified electrochemical response with a detection limit of 0.14 fm and high selectivity was achieved. It is assumed that 2D nanosheets of this type of MOF could offer even better electrochemical sensing.

Porphyrins in 2D Cu‐TCPP MOF sheets can interact via π–π stacking with various aptamers, making them suitable for constructing aptasensors. Qiao et al. utilized a 2D Cu‐TCPP MOF‐modified GCE electrode as an aptasensor for the detection of ochratoxin A (OTA), a mycotoxin that can damage the kidneys.^[^ ^185^ ^]^ To improve sensitivity and enable signal amplification, they further introduced Ag nanoparticles (Ag NPs) and a DNA walker strategy. As a result of this signal amplification approach, they managed to achieve a detection limit of 0.08 fg mL^−1^ and a linear range from 0.10 fg mL^−1^ to 1 µg mL^−1^.

He et al. fabricated a 2D 521‐MOF‐based electrochemical biosensor for the detection of mucin 1 (MUC1), a cancer‐related biomarker, with a low detection limit of 0.12 pg·ml^−1^ and 0.65 pg·ml^−1^ based on electrochemical impedance and SPR, respectively.^[^ ^186^ ^]^ The 2D Zr‐based MOF was synthesized using a polymer surfactant (polyvinyl pyrrolidone), ZrOCl_2_·8H_2_O, and 4′,4''',4'''''‐Nitrilotris[1,1′‐biphenyl]‐4‐carboxylic acid (H_3_NBB) ligands in a mixture of trifluoroacetic acid and diethylformamide. The high sensitivity and selectivity of this aptasensor may be attributed to the effective immobilization of the aptamer, due to the strong affinity between the phosphate groups of the aptamer strand and the Zr ions.

**Figure 11:**
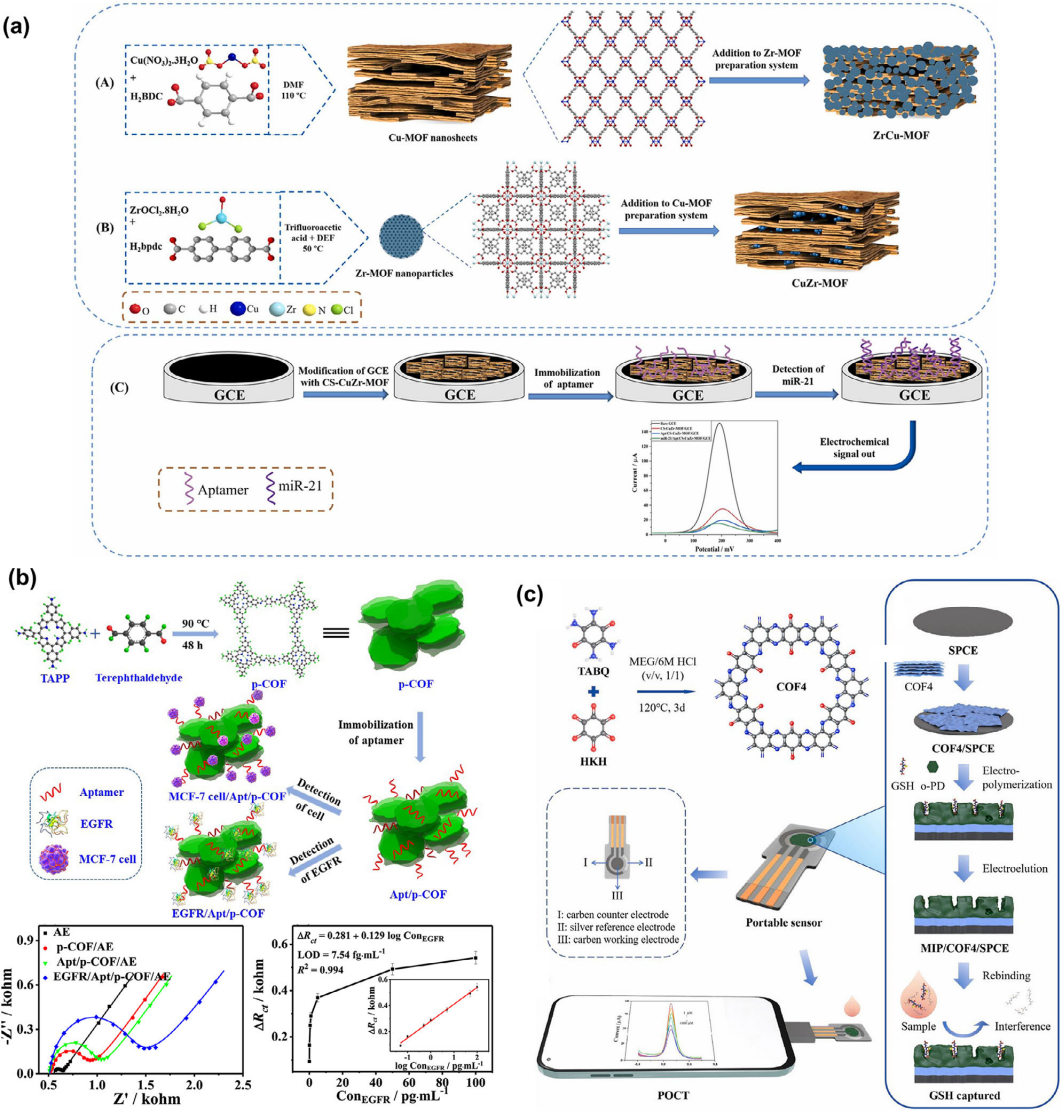
Application of MOFs and COFs for electrochemical biosensing. a) Synthetic procedure of bimetallic CuZr‐MOF nanosheets with schematic illustration of the fabrication and sensing mechanism of an electrochemical biosensor based on CuZr‐MOF nanosheets for detecting miR‐21. Reproduced with permission.^[^ ^187^ ^]^ Copyright 2023, Elsevier. b) Schematic representation of the p‐COF‐based aptasensor for sensing EGFR or MCF‐7 cells with EIS curves of different electrodes for the detection of EGFR, and the dependence of ΔRct on the concentration of EGFR. Reproduced with permission.^[^ ^188^ ^]^ Copyright 2019, Elsevier. c) Synthetic scheme of aza‐fused π‐conjugated COF, along with the fabrication and sensing mechanism of a molecularly imprinted polymer (MIP) based electrochemical sensor for the detection of glutathione using a 2D c‐COF modified screen‐printed carbon electrode (SPCE). Reproduced with permission.^[^ ^189^ ^]^ Copyright 2025, Elsevier.

It is well known that aptamers can be effectively immobilized on the surface of 2D MOF nanosheets through various interactions, such as π–π stacking, hydrogen bonding, metal coordination, van der Waals forces, and electrostatic interactions between the negatively charged nucleic acid sequences and the organic linkers. An electrochemical aptasensor based on bimetallic CuZr‐MOF nanosheets (see **Figure** **11**a) was developed, exhibiting an excellent detection range from 1 zm to 1 pm, with a detection limit of 0.45 zm for miR‐21, a biomarker for cancer diagnosis.^[^ ^187^ ^]^ This outstanding performance can be attributed to the high surface area, large pore size of the MOF nanosheets, and strong interactions between the aptamer strands and the MOF structure.

##### 2D COFs

The porous crystalline materials, COFs, have gained significant attention due to their customizable functionalities, porosity, and stability. Compared to bulk COFs, 2D COF nanosheets are excellent candidates for biosensing because of their shorter electron transfer paths, more exposed outer surfaces, and accessible binding sites.^[^ ^190^ ^]^ Yan et al. fabricated a porphyrin‐based COF (p‐COF) electrochemical aptasensor for detecting epidermal growth factor receptor (EGFR) and living Michigan Cancer Foundation‐7 (MCF‐7) cells. The sensor demonstrated a broad linear detection range of 0.05–1000 pg mL^−1^ for EGFR concentration, with limits of detection (LOD) of 7.54 fg mL^−1^ for EGFR and 61 cells mL^−1^ for MCF‐7 cells (Figure 11b).^[^ ^188^ ^]^ This excellent performance can be attributed to the 2D nanosheet structure, highly conjugated framework, and large pore channels of the p‐COF nanosheets. Wang et al. utilized AuNPs@COF nanosheets on glassy carbon electrodes (GCE) for the electrochemical detection of programmed death‐ligand 1 protein‐positive (PD‐L1+) exosomes, a cancer biomarker, achieving a low detection limit of 38 particles µL^−1^.^[^ ^190^ ^]^ The incorporation of Au NPs into the COF enhances conductivity, hydrophilicity, biocompatibility, and the immobilization of exosome probes. The COFs were synthesized using 5,10,15,20‐tetra(p‐aminophenyl)porphyrin (H2TAPP), 2,4,6‐trimethylbenzaldehyde (TBA), and 4,4′‐biphenyldialdehyde (BPDA), while the Au NPs were grown in situ in an aqueous dispersion of the COF nanosheets. Like 2D MOF nanosheets, 2D COFs can also be excellent candidates for non‐enzymatic biosensing to catalyze the redox reactions of various analytes. Arul et al. utilized an Au‐Ag NPs‐COF composite on GCE to construct a non‐enzymatic H_2_O_2_ biosensor with a limit of detection (LOD) of 0.44 nm (S/N = 3).^[^ ^191^ ^]^ Typically, mono‐ or bimetallic NPs tend to aggregate due to their high surface energy. The 2D COF sheet can serve as a potential platform for anchoring these metal NPs due to its crystallinity and porosity.

Very recently, a molecularly imprinted polymer (MIP)‐based electrochemical sensor was developed for the detection of glutathione using a 2D aza‐fused π‐conjugated COF‐modified screen‐printed carbon electrode (SPCE).^[^ ^189^ ^]^ This c‐COF‐based MIP sensor shows promise due to its low cost, simple preparation and operation, and the high conductivity of the c‐COF (see Figure 11c).

Various 2D MOFs and COFs can be excellent candidates for electrochemical biosensors. Remarkable progress has been made in developing electrochemical biosensors based on these types of 2D materials. However, there is still room for improvement in scalable synthesis, enhancement of electrical conductivity, and broadening of their applications. Moreover, by combining these materials with other 2D materials, taking advantage of their high affinity for biomolecules, which is facilitated by their functional groups, metal ions, micro and nanoporous structures, and ability to donate electrons through π–π stacking, there is significant potential for further applications in electrochemical biosensors.

### 3.5 Transition Metal Oxides (TMOs) and Layered Double Hydroxides (LDHs)

#### 3.5.1 Importance of 2D TMOs and LDHs

Even though metal oxides are earth‐abundant materials, pure and fully monolayered forms of metal oxides are rarely accessible due to their ion‐stabilized lamellar bulk structures. Typically, 2D transition metal oxides (MO_x_) can be categorized as either layered or non‐layered. Common examples of layered MO_x_ are MoO_3_ and *V*_2_O_5_.^[^ ^192^ ^]^ The thermally stable polymorph α‐MoO_3_ of MoO_3_ is the semiconducting layered form, double layers of edge‐sharing MoO_6_ octahedra, vertically held together by weak van der Waals forces, and can be exfoliated into a 2D form, which is a potential candidate for various applications, from electronics, optoelectronics, to sensing applications. α‐MoO_3_ could be a potential 2D semiconducting channel material for FET with improved carrier mobility due to its high dielectric constant and tunable band structure.^[^ ^34^ ^]^ Few‐layered or monolayer planar hexagonal TiO_2_ obtained by Zhang et al. exhibits p‐type semiconducting properties with hole mobilities of up to 950 cm^2^ V^−1^ s^−1^ at ambient temperature.^[^ ^193^ ^]^ TiO_2_, Fe_2_O_3_, and Ni_2_O_3_ are examples of few‐layered or monolayer planar hexagonal 2D MOx. ZnO, a direct band gap (≈3.37 eV) semiconductor, has gained attention among TMOs for its unique optical, electrical, and mechanical properties, such as stable excitons (binding energy of 60 meV), low electron effective mass, strong luminescence, high transparency in the visible range, and tunable n‐type conductivity. These features make ZnO suitable for various applications in photonics, piezotronics, optoelectronics, and sensor technologies.^[^ ^194^, ^195^, ^196^ ^]^ Bulk ZnO contains defects, particularly oxygen and zinc vacancies,^[^ ^194^ ^]^ which strongly influence its electrical and optical properties. Therefore, defect‐free 2D ZnO, with its high surface area, holds great promise for electrical and optoelectronic devices.

2D layered double hydroxides (LDHs), also known as anionic clays, have the general formula M^II^_1‐x_M^III^_x_(OH)_2_(A^n−^)_x/n_.yH_2_O, where M^II^ and M^III^ are divalent and trivalent metal cations, respectively (M^I^ and M^IV^ are also possible), and A^n−^ represents interlayer anions. Bulk LDH is typically formed by stacking positively charged, brucite‐like octahedral layers of metal hydroxides with negatively charged organic or inorganic species between the layers to neutralize the positive charges. Due to the variety of metal ions and anionic species, a vast number of LDHs with distinct properties can be synthesized and applied as 2D nanomaterials for various applications. Specifically, 2D (Ni, Co‐based) LDHs are excellent electrocatalysts for the oxygen evolution reaction (OER) due to their favorable structural composition, ability to form porous films, physicochemical properties, and catalytic activity.^[^ ^20^ ^]^ LDHs are also attractive for use in energy storage and conversion, chemical sensing, optics, magnetics, and biomedical applications.^[^ ^20^, ^197^, ^198^, ^199^, ^200^ ^]^ The main limitation of LDHs is their poor intrinsic electrical conductivity (10^−13^ to 10^−17^ S cm^−1^), which is insufficient for use in various electrical and optoelectrical devices.

#### 3.5.2 Synthesis of Key 2D TMOs and LDHs and Challenges for Large‐Scale Production

**Figure 12:**
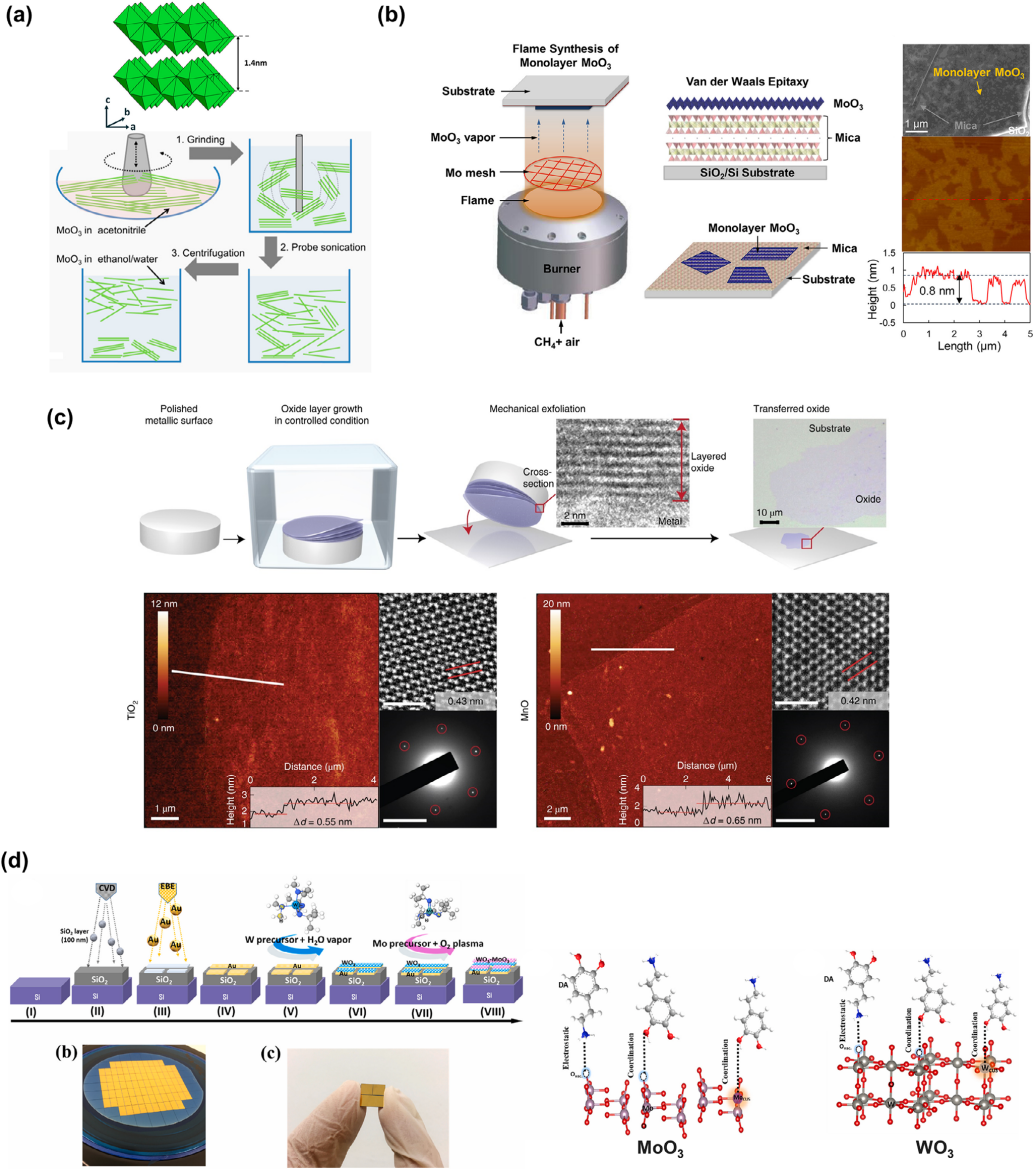
a) The crystal structure of α‐MoO_3_, double layers of edge‐sharing MoO_6_ octahedra, and liquid‐phase exfoliation. Reproduced with permission.^[^ ^34^ ^]^ Copyright 2013, ACS. b) Monolayer or few‐layered MoO_3_ grown on mica through epitaxial van der Waals growth mechanism using CH_4_‐flame. Reproduced with permission.^[^ ^201^ ^]^ Copyright 2017, ACS. c) Schematic procedure of hexagonal layered MOx growth and mechanical exfoliation to 2D layered MOx, with an AFM image and thickness profile; HRTEM image and SAED patterns for TiO_2_ and MnO. For AFM, samples were mechanically exfoliated onto SiO_2_/Si substrates, and for TEM, samples were exfoliated onto holey carbon support grids. Reproduced with permission.^[^ ^193^ ^]^ Copyright 2021, Springer Nature. d) Schematic of the fabrication of a MoO_3_‐WO_3_ heterostructured sensing electrode by atomic layer deposition for dopamine sensing, and the various interactions of dopamine with MoO_3_ and WO_3_. Reproduced under terms of the CC‐BY license.^[^ ^202^ ^]^ Copyright 2023, Lopa et al.

Balendhran et al. utilized three‐step liquid phase exfoliation to obtain 2D α‐MoO_3_ flakes with an average 2.8 nm flake size (**Figure** **12**a).^[^ ^34^ ^]^ Cai et al. reported the synthesis of a monolayer MoO_3_ film via epitaxial vapor deposition of Mo, using a CH_4_ flame and air as oxidizers, on a mica growth substrate. The process is based on a van der Waals epitaxial growth mechanism (Figure 12b).^[^ ^201^ ^]^ Zhang et al. were the first to report the layered planar hexagonal phase of MO_x_ obtained through controlled oxidation at the metal–gas interface.^[^ ^193^ ^]^ Highly crystalline monolayer hexagonal MO_x_ can be mechanically exfoliated from its bulk crystal grown on a substrate, by being physically transferred or pressed onto the surface of another substrate (Figure 12c). However, the growth of MO_x_ is a complicated procedure that requires sophisticated instruments and precise control. Recently, few‐atomic‐layer, defect‐free ZnO nanosheets were reported, produced by liquid‐phase exfoliation of ZnO powder (micron‐sized) using a 1% triethylamine solution in ethanol, through mild sonication for 12 h.^[^ ^196^ ^]^

LDHs can be synthesized and exfoliated through various well‐known methods, such as one‐step hydrothermal/solvothermal processes,^[^ ^203^ ^]^ two‐step topochemical synthesis,^[^ ^204^, ^205^ ^]^ and anion exchange followed by exfoliation (Figure 12d). In this two‐step process, micrometer‐sized, hexagonally shaped brucite‐like LDH (such as Co^2^⁺‐Fe^2^⁺ hydroxide) is first prepared through an HMT hydrolysis reaction. Then, oxidation of the brucite‐like LDH by an oxidizing agent (such as Br_2_ or *I*_2_) converts it to hydrotalcite‐like LDH (such as Co^2^⁺‐Fe^3^⁺ LDH). Excess positive charges in the layer are balanced by I^−^ or Br^−^ ions, which are sandwiched between the layers. After ion exchange and exfoliation, LDH nanosheets can be formed.^[^ ^204^, ^205^ ^]^

#### 3.5.3 Applications of 2D TMDs and LDH in Electrical and Electrochemical Sensors for Biomarker Detection

##### TMOs

2D TMOs provide effective biosensing platforms, particularly for non‐enzymatic biosensors, due to their intrinsic redox activity for catalyzing biomolecules, diverse oxidation states, tunable electronic properties, inherent chemical stability, and biocompatibility.

Lopa et al. prepared a heterostructured WO_3_ and MoO_3_‐based electrode by atomic layer deposition for the electrochemical detection of dopamine, owing to its effective catalytic ability and enhanced surface reaction kinetics resulting from the different semiconducting properties, surface defects, and sub‐stoichiometric phases exhibited by the heterostructure.^[^ ^202^ ^]^ This heterostructured TMO‐based electrochemical sensor demonstrated a low detection limit of 20 nm and shows promise for dopamine detection in clinical biological samples.

A 2D molybdenum trioxide (MoO_3_) FET‐based biosensor was developed using bovine serum albumin (BSA) as a model protein, leveraging the promising high permittivity of 2D α‐MoO_3_ nanoflakes.^[^ ^34^ ^]^ With the assistance of Au NPs, BSA immobilization is achieved due to the strong affinity of gold NPs for BSA. The sensing mechanism can be described as follows: negatively charged BSA produces a negative potential in the active layer. Since the 2D MoO_3_ sensing film consists of an n‐type FET, the channel conductance is reduced due to the negative potential generated by BSA. The electrostatic interaction between positively charged ZnO and negatively charged DNA can effectively immobilize probe DNA.^[^ ^206^, ^207^ ^]^ To enhance function and conductive performance, Yang et al. fabricated an electrochemical biosensor using electrodeposited ZnO (2D nanowalls) film on a thin layer of MoS_2_ for DNA sensing (Figure 17a).^[^ ^206^ ^]^ Their ZnO/MoS_2_ electrochemical biosensor exhibited high sensitivity due to the effective immobilization on the ZnO nanosheet surface and a low detection limit of 0.66 fm.

##### LDHs

Layered double hydroxides (LDHs) are promising nanomaterials for enhancing the performance of electrochemical biosensors due to their positively charged layers, which contain counterions in the interlayer space, electrochemically active sites, layered structure, excellent thermal stability, and ion‐exchange ability. The positively charged LDH layer enables effective immobilization of negatively charged bioreceptors, such as DNA, enzymes, and amino acids, through electrostatic interactions.

For instance, Zn–Al–Cl and Cu–Mn–Cl LDHs have been successfully applied to construct electrochemical biosensors.^[^ ^199^, ^203^, ^208^ ^]^ In Ni/Al LDH, the positively charged Ni is electrochemically active, and the Ni^2+^/Ni^3+^ redox couple in alkaline solution effectively facilitates glucose detection. Li et al. deposited a Ni/Al LDH film on Ti foil via a simple coprecipitation method to create an electrode for a non‐enzymatic electrochemical glucose sensor, achieving a low detection limit of 5 µm.^[^ ^209^ ^]^

**Figure 13:**
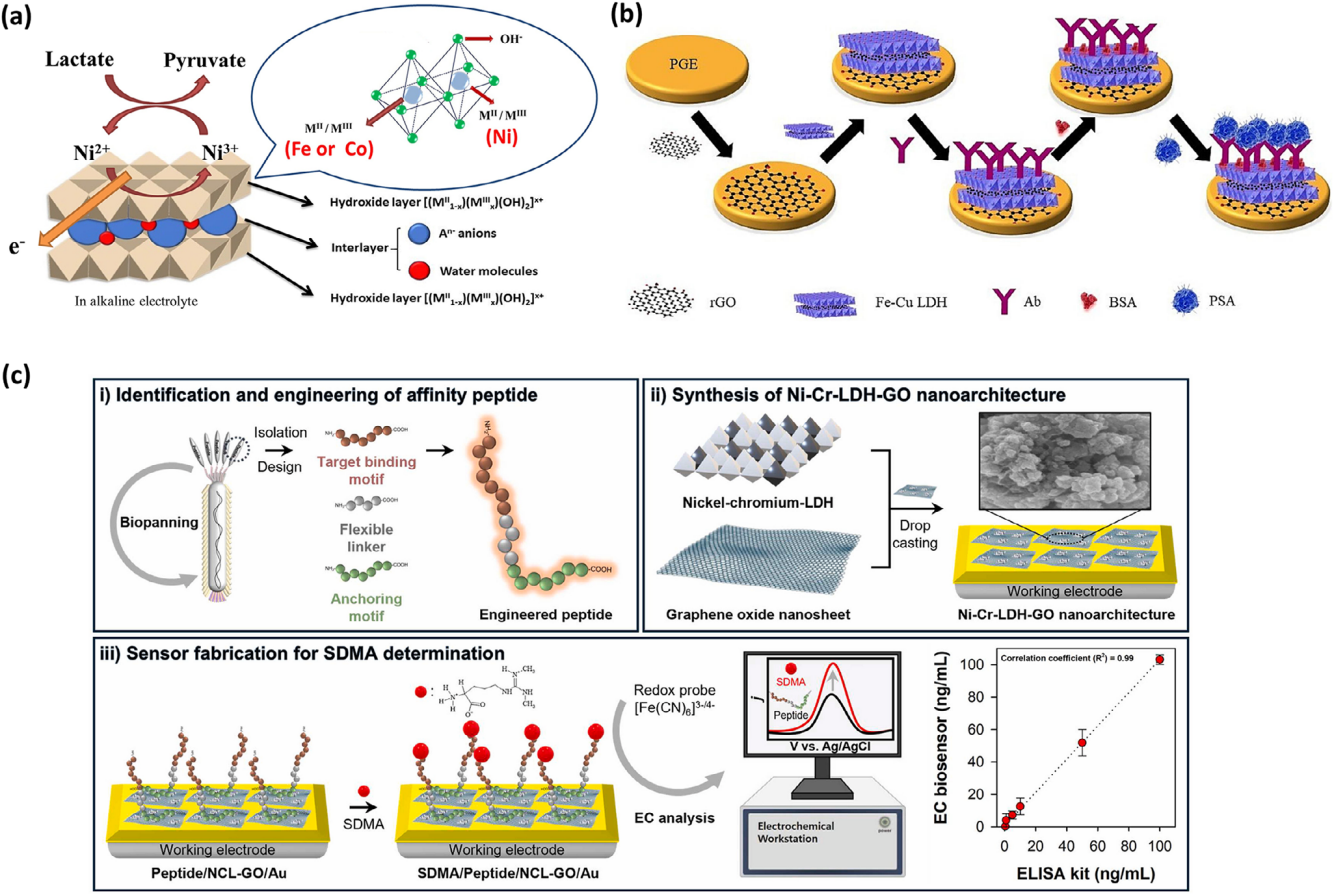
a) Electrocatalytic ability of Ni–Co LDH for lactate oxidation and sensing. Reproduced with permission.^[^ ^210^ ^]^ Copyright 2021, Elsevier. b) Schematic representation of an electrochemical biosensor combining Fe–Cu LDH and rGO for the detection of prostate‐specific antigen (PSA). Reproduced with permission.^[^ ^213^ ^]^ Copyright 2023, Elsevier. c) Schematic of the development of a peptide‐based electrochemical biosensor by combining a 2D–2D nanoarchitecture of nickel–chromium LDH and GO nanosheets with a single linear peptide for the detection of symmetric dimethylarginine (SDMA). Reproduced with permission.^[^ ^214^ ^]^ Copyright 2025, Elsevier.

Ni(Co/Fe)‐based LDH on screen‐printed carbon electrodes (SPCE) was investigated as an enzyme‐free electrochemical sensor for lactate detection. It was found that NiCo‐LDH exhibited superior electrocatalytic activity compared to NiFe‐LDH in lactate oxidation (**Figure** **13**a).^[^ ^210^ ^]^

However, typical limitations of LDHs include poor conductivity, difficulty in separating from solution, and aggregation in the solid state. To enhance conductivity and electron transfer kinetics, the combination of LDHs with nanoparticles and GO/rGO can be effective for biosensing applications. A glassy carbon electrode modified by the self‐assembly of positively charged semiconducting Zn–NiAl LDH sheets with negatively charged rGO exhibits electrocatalytic activity toward biomolecules and can be used to detect dopamine, uric acid, ascorbic acid, and others. Asif et al. used this combination of positively charged Zn–NiAl LDH and negatively charged rGO as an electrochemical biosensor for detecting ascorbic acid, dopamine, and uric acid, achieving detection limits of 13.5, 0.1, and 0.9 nm, respectively, at a signal‐to‐noise ratio of 3.^[^ ^211^ ^]^ This performance is attributed to the synergistic effects of rGO and LDH, including the excellent conductivity from rGO, the intercalation ability of LDH, and the large surface area and high number of active sites. Vajedi et al. used ZnAl‐LDH in combination with CoFe_2_O_4_ NPs, graphene oxide (GO), and chitosan, and deposited it electrophoretically on FTO for a DNA biosensor, achieving an etoposide (ETO) detection limit of 0.0010 µm.^[^ ^212^ ^]^ It is noted that CoFe_2_O_4_ NPs alone tend to aggregate due to magnetic dipole interactions. The electrostatic interaction between the NH_3_^+^ groups of chitosan and the negatively charged phosphate (PO_4_^−^) backbone of DNA is assumed to facilitate effective DNA immobilization. Ghasemi et al. developed a label‐free electrochemical immunosensor for the detection of the prostate cancer biomarker prostate‐specific antigen (PSA) by combining Fe‐Cu‐LDH and rGO as a nanocomposite sensing element (Figure 13b).^[^ ^213^ ^]^ The sensor exhibited high sensitivity, excellent selectivity, and a wide linear detection range of 100 fg mL^−1^ to 10 µg mL^−1^, with a limit of detection of 63.24 fg mL^−1^. This performance was attributed to the synergistic contribution of rGO and LDH, which provided a high surface area for enhanced bioreceptor immobilization, excellent electron transfer ability, and strong electrostatic interactions between the negatively charged PSA antibody and the positively charged Fe‐Cu‐LDH.

Recently, a peptide‐based electrochemical biosensor was developed by combining a 2D–2D nanoarchitecture of nickel‐chromium layered double hydroxide (LDH) and graphene oxide (GO) nanosheets for the detection of symmetric dimethylarginine (SDMA), a biomarker associated with kidney dysfunction (Figure 13c). Enhanced conductivity and improved electron transport pathways were achieved due to the synergistic contributions of both GO and LDH. A single linear peptide was selected to specifically capture and bind SDMA, which further enhanced the biosensing performance, achieving a limit of detection of 0.1 ng mL^−1^ within a range of 0–1 ng mL^−1^.^[^ ^214^ ^]^

### 3.6 Graphitic Carbon Nitride

#### 3.6.1 Importance of 2D Graphitic Carbon Nitride

**Figure 14:**
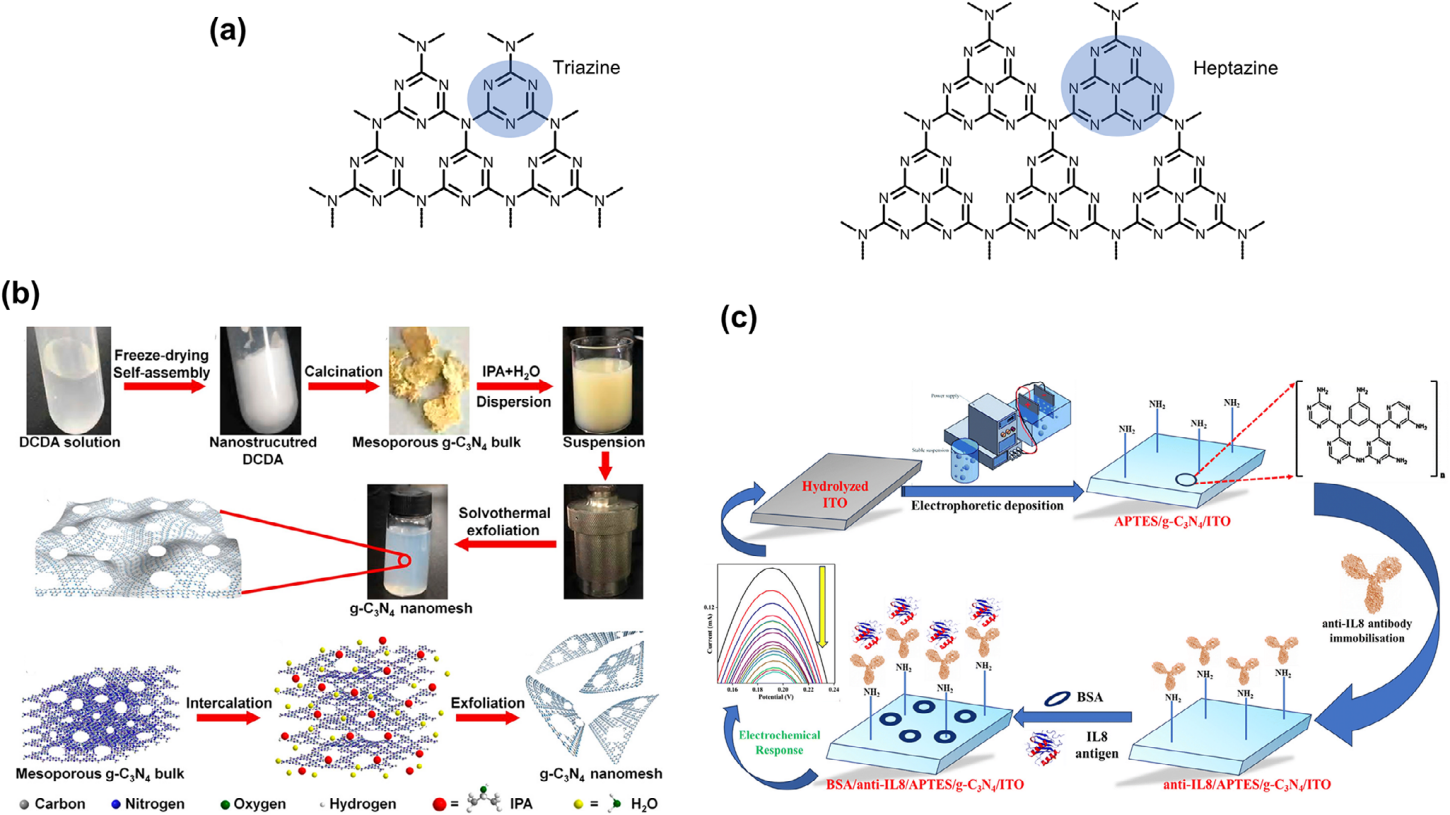
a) Structures of graphitic carbon nitride (g‐C_3_N_4_) based on triazine and heptazine (tri‐s‐triazine). b) A schematic representation of bulk g‐C_3_N_4_ synthesis using dicyandiamide (DCDA) as a precursor and the solvothermal exfoliation technique for obtaining mesoporous 2D nanosheets. (b): Reproduced with permission.^[^ ^220^ ^]^ Copyright 2016, ACS. c) Utilization of APTES‐functionalized g‐C_3_N_4_ for the electrochemical detection of interleukin‐8. Reproduced with permission.^[^ ^223^ ^]^ Copyright 2025, RSC.

According to theoretical calculations, five polymorphs of carbon nitrides (α‐C_3_N_4_, β‐C_3_N_4_, cubic‐C_3_N_4_, pseudocubic‐C_3_N_4_, and g‐C_3_N_4_) can naturally exist.^[^ ^215^ ^]^ Among these five polymorphs, g‐C_3_N_4_ (graphitic carbon nitride) has the narrowest bandgap of 2.7 eV, with excellent light absorption abilities, as well as outstanding thermal stability (up to 600 °C in air) and chemical stability.^[^ ^36^ ^]^ The two types of structural isomers of g‐C_3_N_4_, namely tri‐s‐triazine (tri‐ring of C_6_N_7_) and s‐triazine (ring of C_3_N_3_), can be formed (**Figure** **14**a) depending on the precursors and condensation methods, with tri‐s‐triazine being the most stable. The highly ordered melon‐type polymeric C_3_N_4_ structure has pendant amino groups and is formed first. Further condensation results in a denser and less defective C_3_N_4_ structure with tri‐s‐triazine building blocks.^[^ ^216^ ^]^ In contrast to graphene, the g‐C_3_H_4_ nanosheet is composed of tri‐s‐triazine units bridged by amino groups, with dangling hydrogen atoms in the C–N layer and periodic vacancies within the lattice structure.^[^ ^217^, ^218^, ^219^ ^]^ The interlayer distance of g‐C_3_N_4_ is ≈0.326 nm, ≈3% denser than crystalline graphite (d ≈ 0.335 nm). 2D single‐atom nanosheets have unique physical properties, such as greater surface area, higher intrinsic carrier mobility, significant changes in the energy band, and many other advantages compared to their bulk counterparts. For instance, compared to bulk g‐C_3_H_4_, the band gap of single‐layer g‐C_3_H_4_ nanomesh increases by 0.16 eV due to the quantum confinement effect. This increase in the band gap shifts the conduction band to a more negative potential, providing a higher thermodynamic driving force for photocatalytic reduction reactions. Additionally, the single‐layer g‐C_3_H_4_ nanomesh exhibits enhanced light‐harvesting ability due to the multiple scattering effect and the presence of numerous defect sites on the mesoporous surface.^[^ ^220^ ^]^ Due to the presence of many unsaturated sites and dangling hydrogen bonds at the edges of g‐C_3_N_4_ nanosheets, they can be further modified for various applications and are also potential candidates for biosensing applications.

#### 3.6.2 Synthesis of 2D g‐C_3_N_4_: Methods and Challenges

Bulk g‐C_3_N_4_ is typically obtained through the polymerization of cyanamide, dicyandiamide, or melamine.^[^ ^221^ ^]^ The most suitable synthetic method for g‐C_3_N_4_ is the temperature‐initiated condensation of dicyanodiamine using a LiCl and KCl salt melt as the solvent. This process results in layered g‐C_3_N_4_ with hexagonally arranged s‐heptazine (C_6_N_7_) units, where the C–N bonds in the layer are covalent, and the layers are stacked by van der Waals interactions in a graphitic staggered fashion.^[^ ^222^ ^]^ Similar to the various exfoliation methods used for graphite and TMDs to obtain 2D nanosheets, single or few layers of g‐C_3_N_4_ can also be produced. Han et al. reported that single‐layer g‐C_3_H_4_ nanomesh, obtained through the solvothermal exfoliation of mesoporous bulk g‐C_3_H_4_ (Figure 14b), demonstrates excellent photocatalytic hydrogen evolution.^[^ ^220^ ^]^

#### 3.6.3 Applications of 2D Graphitic Carbon Nitride in Electrical and Electrochemical Sensors for Biomarker Detection

2D g‐C_3_N_4_ is primarily used in photoelectrochemical biosensors due to its effective photocatalytic activity. However, its excellent biocompatibility, chemical stability, and low cost also make it attractive for electrochemical biosensors, particularly for non‐enzymatic biosensing. For the electrochemical detection of dopamine, Zou et al. fabricated a biosensor based on a 2D‐g‐C_3_N_4_/CuO nanocomposite coated on a GCE.^[^ ^224^ ^]^ The 2D‐g‐C_3_N_4_/CuO nanocomposite was synthesized via pyrolysis using melamine to form 2D g‐C_3_N_4_ in the presence of cupric acetate monohydrate, the precursor for CuO nanomaterials. Cu–N coordination between CuO and g‐C_3_N_4_ can occur. The enhanced conductivity of the g‐C_3_N_4_/CuO nanocomposite, compared to pure g‐C_3_N_4_, may accelerate the charge transfer process. The increased conductivity and the narrowing of the g‐C_3_N_4_ band gap due to the presence of CuO nanomaterials likely contribute to the high sensitivity of the electrochemical measurement for dopamine sensing.

Recently, Yadav et al. utilized APTES‐functionalized g‐C_3_N_4_ for label‐free sensing of interleukin‐8 in saliva, which may be effective for the early diagnosis of oral cancer (Figure 14c).^[^ ^223^ ^]^ They functionalized APTES on the g‐C_3_N_4_ surface, where the amine group in the attached APTES was covalently coupled to the carboxyl (─COOH) groups in the activated anti‐IL8 antibody through EDC/NHS coupling. Generally, g‐C_3_N_4_ does not have surface–OH groups that can covalently bond with APTES without prior functionalization by oxidation, O_2_‐plasma treatment, or other methods. In their functionalization process, dispersed g‐C_3_N_4_ in isopropanol (IPA) was treated with APTES at 60 °C, a condition that is assumed insufficient for oxidation to introduce ─OH groups. Furthermore, they did not characterize their functionalized g‐C_3_N_4_ to confirm the surface modification.^[^ ^223^ ^]^

### 3.7 Hexagonal Boron Nitride (h‐BN)

Hexagonal boron nitride (h‐BN) is an insulating, layered material composed of partially ionic, sp^2^‐bonded alternating boron and nitrogen atoms in a hexagonal honeycomb arrangement. The layers of h‐BN are stacked via van der Waals interactions in an AA stacking pattern^[^ ^225^ ^]^ to form a bulk crystal. It is often referred to as “white graphene” because of its layered nature and structural similarity to graphene.

#### 3.7.1 Importance of 2D h‐BN

Unlike graphene, which has zero bandgap and semimetal characteristics, h‐BN exhibits insulating properties with a wide bandgap of 5.9 eV.^[^ ^35^ ^]^ 2D h‐BN has an atomically flat surface, free from dangling bonds and charge traps, and it also exhibits high chemical stability and large surface optical phonons. Additionally, its lattice constant is similar to that of graphite. 2D h‐BN shows promise as an insulating layer for graphene‐FETs, offering advantages over SiO_2_. The use of SiO_2_ as an insulating layer in graphene‐based devices can diminish graphene's intrinsic properties, such as mobility, and limit performance due to electron scattering caused by the dangling bonds, roughness of the SiO_2_ surface, and optical phonons.^[^ ^226^, ^227^, ^228^, ^229^ ^]^ Several studies have reported that using 2D h‐BN as an insulating layer in graphene‐FETs increases graphene mobility and improves carrier inhomogeneity by an order of magnitude compared to SiO_2_.^[^ ^226^, ^230^ ^]^ Not only in graphene‐based FETs, but increased mobility and reduced hysteresis were also observed in 2D TMD‐FETs (such as MoS_2_) when using an h‐BN insulating layer.^[^ ^231^ ^]^ This improvement is due to the atomically flat surface of h‐BN, which is free from charge traps and highly chemically stable. It is noted that a monolayer or a few layers of h‐BN are not thick enough to reduce intrinsic defects and the tunneling effect.^[^ ^232^, ^233^ ^]^ Therefore, multilayer h‐BN can be more effective as an insulating layer than single‐layered h‐BN, as a thicker film reduces defects and minimizes the tunneling effect.

#### 3.7.2 Synthesis of 2D h‐BN: Methods and Challenges

**Figure 15:**
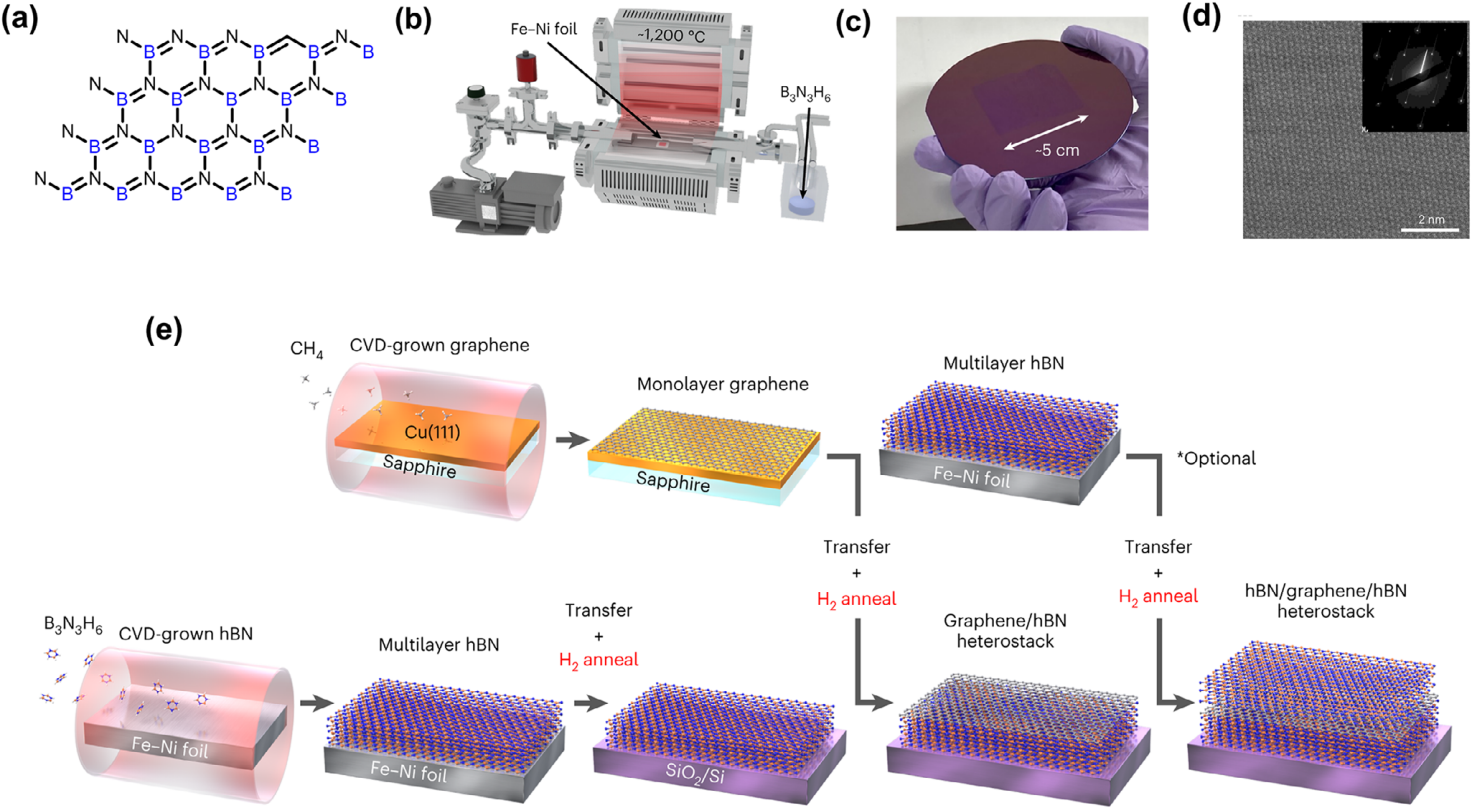
a) Structure of hexagonal boron nitride (h‐BN). b) CVD setup for growing multilayer h‐BN on Fe–Ni foil. c) Digital image of multilayer h‐BN after transfer onto a SiO_2_/Si substrate. d) STEM image and SAED pattern of multilayer h‐BN. e) Schematic of the preparation of heterostructured h‐BN/graphene/h‐BN on a SiO_2_/Si substrate by transferring CVD‐grown graphene and multilayer h‐BN films for graphene‐FET. (b)–(e): Reproduced under terms of the CC‐BY license.^[^ ^234^ ^]^ Copyright 2023, Fukamachi et al.

There are several reports on the synthesis of uniform multilayer h‐BN using chemical vapor deposition (CVD), a bottom–up approach,^[^ ^233^, ^234^ ^]^ which ensures the excellent quality of 2D h‐BN. Fukamachi et al. demonstrated the transfer of CVD‐grown multilayer h‐BN (**Figure** **15**b–e) to integrate with CVD‐grown graphene for constructing graphene FETs.^[^ ^234^ ^]^ Hirata et al. prepared high‐quality h‐BN nanosheet films using a physical vapor deposition technique that combined magnetron sputtering and annealing. They found that an optimal annealing temperature of 900 °C resulted in the formation of a well‐ordered h‐BN structure.^[^ ^235^ ^]^ Even though the quality of 2D h‐BN films is better with CVD or PVD techniques, an effective top–down approach for the large‐scale production of h‐BN nanosheets is more practical for the convenient application of various solution‐processable devices and cost reduction. There are some reports of obtaining 2D h‐BN using sonication‐assisted liquid‐phase exfoliation;^[^ ^100^, ^236^ ^]^ however, the quality, production yield, and stability still need further improvement. Ye et al. reported the liquid‐phase exfoliation of h‐BN in common organic solvents using a hyperbranched polyethylene stabilizer, which prevents the aggregation of h‐BN nanosheets through weak non‐covalent CH–π interactions.^[^ ^237^ ^]^

#### 3.7.3 Applications of 2D h‐BN in Electrical and Electrochemical Sensors for Biomarker Detection

Since h‐BN is an electrically insulating (having a wide band gap) material with excellent mechanical strength and inertness, it shows promise primarily as a gate dielectric in 2D FET biosensors. Additionally, it can serve as an encapsulant and passivation layer to protect air‐sensitive materials. Despite the wide band gap of h‐BN, numerous attempts have been made to use h‐BN for the electrocatalytic detection of biomolecules, such as dopamine,^[^ ^238^ ^]^ ascorbic acid, and uric acid,^[^ ^239^ ^]^ based on the consideration that high‐density defects and active surface groups formed during various synthesis methods can narrow the band gap^[^ ^240^, ^241^ ^]^ and enhance electrocatalytic activity. Li et al. synthesized flake h‐BN for the electrochemical detection of ascorbic acid, dopamine, and uric acid, achieving detection limits of 3.77, 0.02, and 0.15 µm, respectively.^[^ ^239^ ^]^

### 3.8 2D Xenens

Beyond graphene, mono‐elemental 2D materials are also classified as Xenes, where X can be an element from group II to VI.^[^ ^242^, ^243^, ^244^, ^245^ ^]^ Recently noble metal Au‐2D membrane was also reported by a top–down approach.^[^ ^246^ ^]^ Phosphorene, Borophene (Group III Xene), Silicene, and Germanene (Group IV Xenes) are potential monoelemental 2D materials for electrical and electrochemical biosensors are discussed here.

#### 3.8.1 Black Phosphorus/ Phosphorene

##### Importance of Phosphorene as a 2D Material

Black phosphorus, the stable layered allotrope of phosphorus, consists of an orthorhombic structure where each layer is built from sp^3^‐hybridized phosphorus atoms arranged in a puckered honeycomb lattice. The layers are stacked via van der Waals interactions. It is a p‐type semiconducting material and can be mechanically exfoliated to mono‐ or few‐layer forms, similar to graphene and MoS_2_.^[^ ^200^ ^]^ In contrast to graphite, the spin–orbit interaction in black phosphorus is more pronounced due to the heavier phosphorus atoms compared to carbon. Additionally, the layers are not completely flat like graphene but are puckered due to sp^3^‐hybridization. Mono‐ or few‐layered 2D materials of black phosphorus are also known as phosphorene.^[^ ^247^ ^]^ Black phosphorus is considered a direct or nearly direct bandgap semiconductor, with its bandgap being tunable depending on the number of layers.^[^ ^248^ ^]^ The band gap of a monolayer of black phosphorus is wider (≈1.51 eV) compared to that of five layers (≈0.59 eV) and bulk materials. In addition to its semiconducting nature, the carrier mobility of monolayer black phosphorus can be exceptionally high, ranging from 10000 to 26000 cm^2^V^−1^s^−1^ according to theoretical predictions.^[^ ^249^ ^]^ In comparison, semiconducting TMDs such as MoS_2_ have a mobility of ≈200 cm^2^V^−1^s^−1^, which can be improved up to ≈500 cm^2^V^−1^s^−1^, much lower than that of monolayer black phosphorus and graphene. Another unique property of monolayer or few‐layer black phosphorus is its strong anisotropic electrical and optical transport, which arises from the puckered surface caused by sp^3^ hybridization. The conductivity is generally higher in the direction perpendicular to the troughs, and holes are more mobile than electrons.^[^ ^249^ ^]^

##### Synthesis of Phosphorene: Methods and Challenges

**Figure 16:**
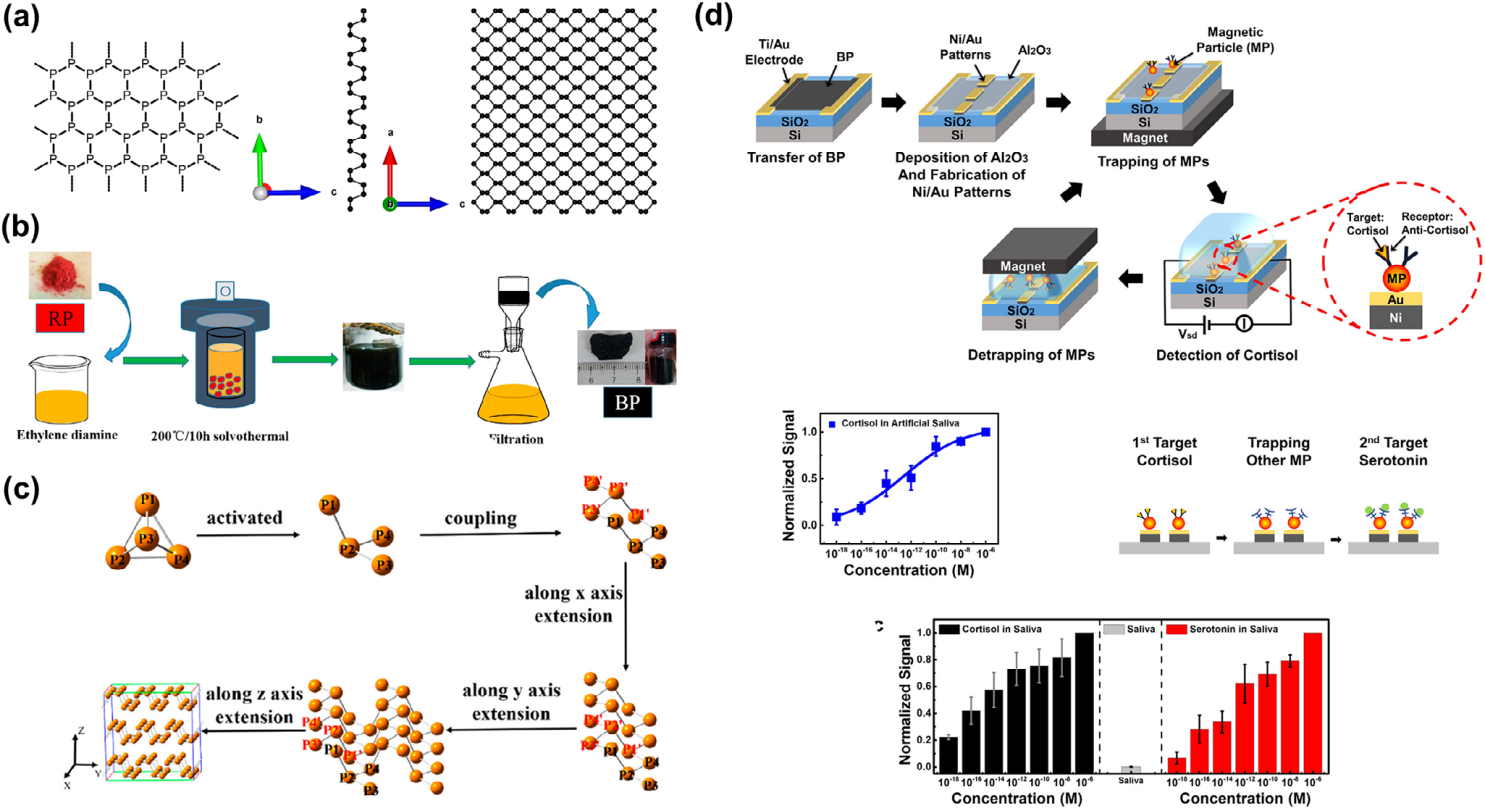
a) Chemical and crystal structure of black phosphorus. b) A schematic illustration of the solvothermal process using red phosphorus as the precursor material, and c) the possible black phosphorus formation mechanism through the activation of P_4_ by ethylenediamine. (b) and (c): Reproduced with permission.^[^ ^254^ ^]^ Copyright 2020, ACS. d) Schematics of a black phosphorus‐based reusable FET for monitoring different hormones in artificial saliva, with a normalized response at varying concentrations. Reproduced with permission.^[^ ^255^ ^]^ Copyright 2024, ACS.

Black phosphorus is typically synthesized from white or red phosphorus under high temperature and pressure.^[^ ^250^, ^251^ ^]^ Although 2D nanosheets can be exfoliated from bulk phosphorus using both top–down methods (such as mechanical exfoliation and liquid‐phase exfoliation with NMP solvent) and bottom–up methods (such as chemical vapor deposition and chemical processes),^[^ ^252^ ^]^ commercial bulk black phosphorus remains quite expensive. Partially oxidized black phosphorus nanosheets with 2–18 atomic layers and a layer‐to‐layer spacing of ≈0.53 nm have been reported using a solvothermal process with ethylenediamine as the solvent and white or black phosphorus as the starting materials (**Figure** **16**b).^[^ ^253^, ^254^ ^]^ Ethylene diamine activates P_4_ by transferring an electron from the HOMO of ethylene diamine (the electron donor) to the LUMO of the P_4_ molecule (the electron acceptor). Coupling and extension in different directions then lead to black phosphorus (Figure 16c).^[^ ^254^ ^]^

This process significantly reduces the cost of obtaining multilayered black phosphorus nanosheets. The functionalities and dispersibility of partially oxidized black phosphorus nanosheets for further solution processing remain unexplored. Further research is needed to determine cost‐effective and large‐scale production methods for monolayer or few‐layered 2D phosphorene nanosheets for electronic, optoelectronic, and biomedical applications.

##### Applications of Phosphorene in Electrical and Electrochemical Sensors for Biomarker Detection

Few‐layered black phosphorus, a stable and unreactive allotrope of phosphorus, is a promising candidate for the channel material in high‐performance FETs due to its direct bandgap semiconducting properties and excellent carrier mobility.^[^ ^256^ ^]^ These properties enable features such as low noise and a high on‐off ratio. However, functionalizing BP for the immobilization of bioreceptors and ensuring its environmental stability under ambient conditions can be challenging. Recently, Lee et al. fabricated a reusable black phosphorus‐based FET biosensor for cortisol detection.^[^ ^255^ ^]^ An Al_2_O_3_ passivation layer was deposited on the black phosphorus layer to protect the channel material from oxygenated molecules while preserving its electrical properties. Ferromagnetic Ni patterns (10 nm Au on 30 nm Ni) were then fabricated to trap N‐cyclohexyl‐N'‐(2‐morpholinoethyl)carbodiimide metho‐p‐toluenesulfonate (CMC)‐functionalized magnetic particles, which selectively bind to anti‐cortisol through amide bond formation. The sensor can be reused (up to 8 times) by detaching the magnetic particles with a magnet, significantly reducing the cost of detection (Figure 16d).^[^ ^255^ ^]^ The electrochemical detection of uric acid, within a detection range of 1–1000 µm and a detection limit of 0.33 µm, was reported using black phosphorus nanosheets modified glassy carbon electrodes through differential pulse voltammetry (DPV). Black phosphorus nanosheets were exfoliated from bulk phosphorus using sonication‐assisted liquid exfoliation in the organic solvent NMP.^[^ ^257^ ^]^

#### 3.8.2 Borophene

##### Importance of Borophene as a 2D Material

Boron, the fifth element in the periodic table, lies between metallic beryllium and nonmetallic carbon and has the electronic configuration [He]2s^2^2p^1^. Because of its single 2p electron, it can favor metallicity; however, its orbital radius is close to the 2s state, which allows for non‐metallicity. This unique electronic configuration enables the formation of covalent two‐center bonds and metallic‐like multi‐center bonds in the bulk phase. The robust bonding capabilities of boron favor the formation of various boron nanostructures, such as 0D clusters, 1D nanotubes, and 2D monolayer sheets.^[^ ^258^ ^]^ Theoretically, various crystalline structures of planar 2D boron can exist, such as α sheets, β sheets, and γ sheets.^[^ ^259^, ^260^, ^261^ ^]^ Unlike graphene, which has a honeycomb lattice, 2D boron layers are generally composed of a triangular grid with a pattern of hollow hexagons. Borophene is interesting because of its metallic characteristics, which complement semimetallic graphene, insulating h‐BN, and semiconducting TMDs. Due to its covalent bonds, which provide measurable strength, and metallic‐like multicenter bonds, which offer fluxional behavior and anisotropy, borophene shows unique mechanical properties. The *v*_1/6_ sheet exhibits ideal strength along and across the hollow hexagonal rows, remarkably higher than phosphorene and silicene, and similar to MoS_2_, though still lower than graphene.^[^ ^258^ ^]^ It is noted that when borophene exceeds the critical strain, it does not exhibit structural failure, unlike other 2D materials, making borophene a potential candidate for flexible electronics; however, a structural phase change may occur. The remarkable in‐plane stiffness and unique out‐of‐plane flexibility of borophene can make it suitable for reinforcement in composites or hybrid materials.

##### Current Synthetic Methods and Challenges in the Large‐Scale Production of Borophene

Borophene possesses inherent polymorphism and is comparatively less stable than its bulk phase. It is a synthetic 2D material with no bulk layered form and, therefore, cannot be extracted by exfoliation from bulk layered materials. Theoretically, 2D boron nanosheets can be grown on metal substrates such as Ag, Cu, and Au due to the stabilization of sp^2^‐hybridization by metal passivation.^[^ ^259^, ^262^ ^]^ Tai et al. were the first to synthesize 2D boron sheets on a Cu substrate using traditional CVD methods.^[^ ^263^ ^]^ However, the thickness was 0.8 nm, not a monatomic layer. Manix et al. successfully prepared an atomically thin 2D boron sheet on an Ag substrate using molecular beam epitaxy (MBE) under ultrahigh vacuum conditions.^[^ ^264^ ^]^ Feng et al. also prepared 2D boron sheets similarly on an Ag(111) surface. Their sheets possess both β_12_ and χ^3^ structures, exhibiting a triangular lattice but with different arrangements of periodic holes.^[^ ^259^ ^]^ The transfer process of grown borophene from an Ag substrate is similar to the mechanical exfoliation of graphene.^[^ ^258^ ^]^ Kiraly et al. reported borophene grown on Au(111) substrates with metallic electronic properties.^[^ ^265^ ^]^ Recently, Wu et al. grew multilayer borophene nanosheets (thickness ≈9.6 nm), possessing semiconducting α’‐4H‐borophene and metallic β_12_‐borophene crystal structures, on aluminum foil using the CVD method. They also claimed that their resulting borophene nanosheet exhibits anisotropic memristive behavior.^[^ ^266^ ^]^ Currently, obtaining 2D boron sheets relies on the optimization of CVD and MBE methods. High‐quality 2D nanosheets can obviously be obtained from CVD, which is one of the preferred choices for electronic applications. However, there is still a lack of large‐scale production of borophene due to the precise optimization and improvement required for the CVD and transfer processes. Therefore, systematically studying factors such as how boron arranges on the substrate, identifying stable polymorphs (such as α, β, and χ sheets), and optimizing the experimental conditions for successfully growing high‐quality borophene with large‐scale synthesis need further exploration. It is noteworthy that, despite the absence of a bulk layered form of boron, several attempts have been made to prepare 2D boron sheets via liquid‐phase exfoliation from commercial boron powder.^[^ ^267^, ^268^ ^]^ Recently, Lin et al. synthesized β_12_‐borophene single‐crystalline sheets (flake size ≈3 µm, with an average thickness of ≤10 atomic layers) using liquid‐phase exfoliation in NMP solvent with the aid of sonication.^[^ ^268^ ^]^ However, this method is not suitable for producing truly atomically thin mono‐ or few‐layered borophene 2D materials.

**Figure 17:**
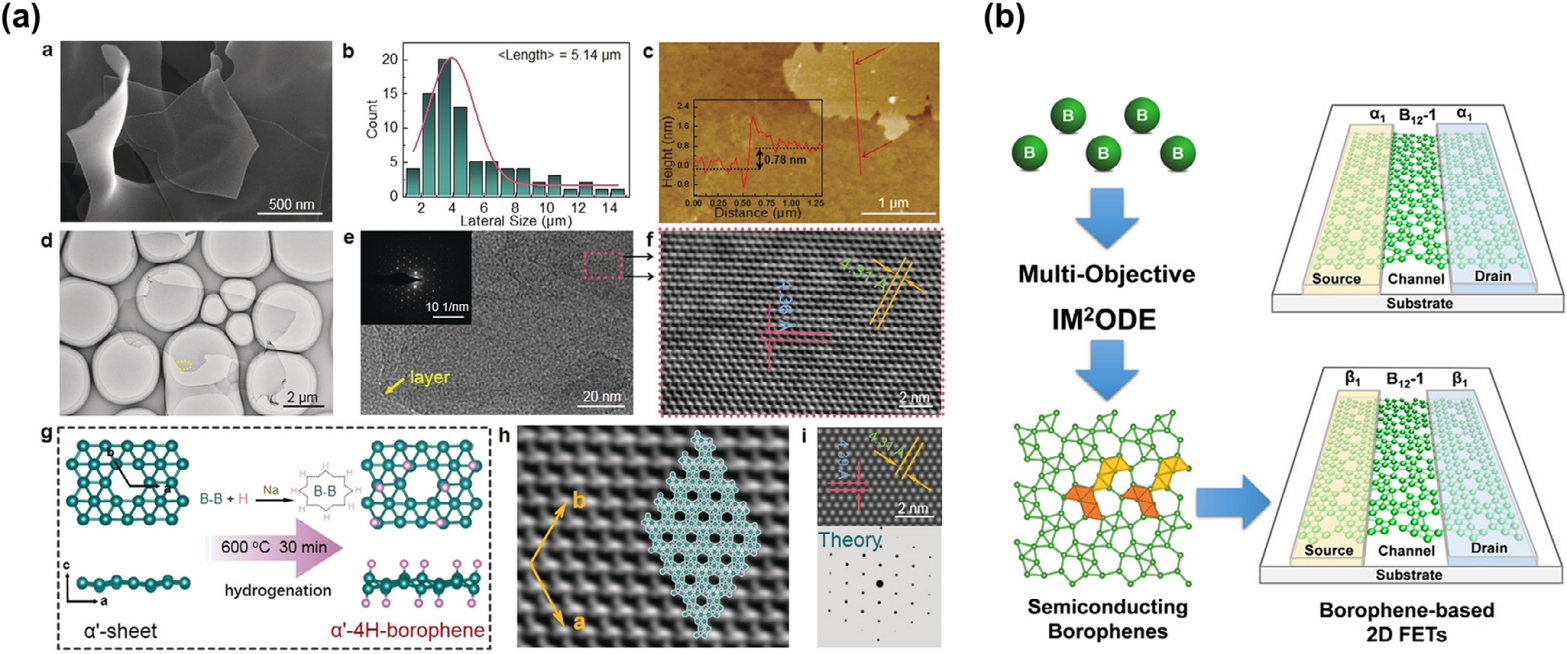
a) Characterization of hydrogenated borophene (ά‐4H‐borophene) by SEM, AFM, TEM, and HRTEM prepared by in situ thermal decomposition at 600 °C. Reproduced with permission.^[^ ^269^ ^]^ Copyright 2020, Wiley. b) Theoretical consideration of constructing FET by semiconducting and stable B_12_‐1 borophene phase. Reproduced with permission.^[^ ^270^ ^]^ Copyright 2021, ACS.

Hydrogenation can be effective to enhance the stability and open the band gap of 2D materials. Recently, semiconducting hydrogenated borophene was synthesized via in situ thermal decomposition of sodium borohydride. A three‐step heating process at 490 °C, 550 °C, and 600 °C produced high‐quality hydrogenated borophene, as confirmed by SEM, AFM, TEM, and HRTEM analyses. (**Figure** **17**a)^[^ ^269^ ^]^

##### Applications of Borophene in Electrical and Electrochemical Biosensors

Theoretically, it has been found that fully boron‐sheet‐based 2D FETs are possible by combining the semiconducting and stable B_12_‐1 borophene phase with a metallic borophene phase (Figure 17b).^[^ ^270^ ^]^ This opens up opportunities to explore stable semiconducting borophenes, such as hydrogenated borophene,^[^ ^269^ ^]^ for future application in electrical FET‐based biosensing. Theoretical DFT investigations have shown that the adsorption of nucleobases can alter the conductivity of the χ_3_‐borophene sheet, with guanine and cytosine being the most responsive. These theoretical studies demonstrate the potential of χ_3_‐borophene sheets for detecting DNA molecules.^[^ ^271^ ^]^ Hydrogenated borophene, prepared via in situ thermal decomposition of NaBH_4_, has already demonstrated effectiveness as a 2D material for NO_2_ gas sensing^[^ ^272^ ^]^ and wearable pressure sensing^[^ ^273^ ^]^ applications. However, its potential for biosensing has not yet been explored and could be promising for both electrical and electrochemical biosensing applications. There are several reports demonstrating the electrochemical biosensing capabilities of boron and boron‐based nanocomposites, such as for non‐enzymatic glucose sensing^[^ ^274^ ^]^ and the detection of Omicron RNA.^[^ ^275^ ^]^ However, in all these cases, the boron nanosheets were prepared by liquid‐phase exfoliation from commercial crystalline boron powder, which may not be suitable for producing single‐ or few‐layered 2D borophene. Therefore, high‐quality, atomically thin borophene materials grown via techniques such as CVD or MBE deserve further investigation for biosensing applications. Among the various borophene phases, semiconducting stable forms and hydrogenated borophene have considerable potential for the development of future biosensors targeting a wide range of biomarkers.

#### 3.8.3 Group‐IV 2D Xenes and Xanes

##### Group‐IV 2D Xenes

Silicene, Germanene, stanene, and plumbene are atom‐thick monoelemental 2D nanosheets possessing buckled honeycomb structures. They show quantum spin Hall (QSH) effect due to the spin‐orbital coupling (SOC), at the Dirac points, fundamental band gaps open up, which can be tuned by applying external fields, and can induce a topological phase transition. Specifically, germanene, stanene, and plumbene can be interesting because of possess larger SOC, making them possible as robust topological insulators and showing QSH effect near or room temperature or even above.^[^ ^276^ ^]^ In this review, the discussion is limited to silicene and germanene.

- *Silicene*

In 1994, the possibilities of the corrugated atomic stage of 2D Si and Ge were theoretically predicted.^[^ ^277^ ^]^ Silicene is a one‐atom‐thick crystal structure of silicon. Unlike graphene, which has a flat honeycomb lattice with sp^2^ hybridization, silicene possesses a honeycomb lattice with a low‐buckled structure (out‐of‐plane displacement of ∼0.44 Å).^[^ ^278^, ^279^ ^]^ This buckling occurs due to the longer Si–Si interatomic distance, which weakens π‐bonding and favors partial sp^3^ hybridization alongside sp^2^ hybridization. The competitive interplay between sp^3^ and sp^2^ hybridization allows silicene to exhibit various structures on different substrates depending on the synthetic conditions. The common and stable overlayer structure of silicene on Ag(111) can be (4 × 4).^[^ ^280^, ^281^ ^]^ Other observed and reproducible phases include (√13 × √13)R13.9°, √3 × √3, √7 × √7, and (2√3 × 2√3)R30°.^[^ ^282^, ^283^, ^284^ ^]^ Silicene exhibits a strong SOC of around 1.55 meV, which is significantly higher than that of graphene. The buckled honeycomb structure, combined with the strong SOC effect, endows silicene with unique properties such as a tunable bandgap, spin‐polarized edge states, and topologically nontrivial electronic states. Due to its nontrivial electronic structure and strong SOC, silicene is predicted to induce the QSH effect. Silicene is generally considered a semimetal with graphene‐like Dirac fermions.^[^ ^242^ ^]^ However, its bandgap can be opened or tuned through external electric fields, growth on different substrates,^[^ ^285^ ^]^ and chemical functionalization. The flexibility and stretchability of silicene, attributed to its buckled structure, make it a promising material for flexible electronics. However, compared to graphene, silicene has lower mechanical strength and thermal conductivity. Moreover, silicene is more prone to oxidation and is unstable under ambient conditions. Due to its high reactivity, functionalization, such as hydrogenation and oxidation, can be easily performed on silicene.

- *Germanene*

A single‐atom‐thick germanium nanosheet, known as germanene, is predicted to be a Dirac fermion material similar to graphene. Germanene is a group IV 2D Xene with a buckled honeycomb structure, like silicene, exhibiting mixed sp^2^–sp^3^ hybridization and semimetallic behavior. The buckling height of germanene is greater than that of silicene, which makes germanene more flexible. Germanene also exhibits stronger SOC than *Xene*, resulting in a larger bandgap opening under external electric fields. This property makes it a promising material for QSH effects and topological insulator applications. Therefore, the combination of its buckled honeycomb structure, strong SOC, and tunable bandgap makes germanene unique for flexible electronic applications.

However, high reactivity, susceptibility to oxidation, and instability under ambient conditions remain major challenges for the synthesis and utilization of this 2D material. On the other hand, functionalization, hybridization, and doping can be effective strategies to tune the bandgap and enhance the properties of germanene for various promising applications.

##### Current Synthetic Methods and Challenges in the Large‐Scale Production of Group IV Xenes

- *Silicene*

**Figure 18:**
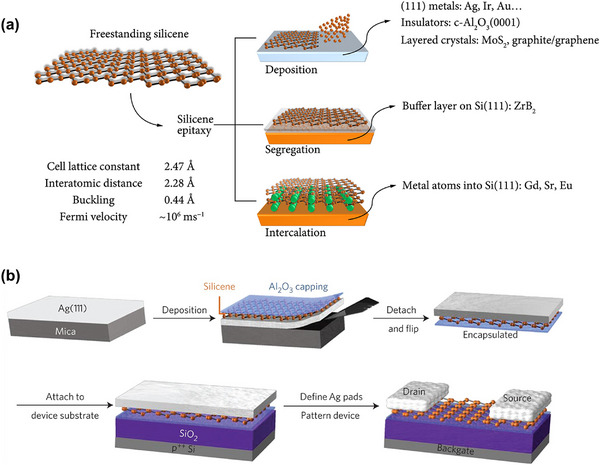
a) Overview of 2D silicene nanosheet growth procedures on different substrates and by various methods. Reproduced under terms of the CC‐BY license.^[^ ^287^ ^]^ Copyright 2019, Grazianetti et al. b) Fabrication procedure of a FET device by encapsulating a silicene layer with Al_2_O_3_, grown on an Ag(111) film supported on Mica. Reproduced with permission.^[^ ^291^ ^]^ Copyright 2015, Springer Nature Limited.

Generally, silicene cannot be produced by exfoliation from bulk materials because there are no analogous layered bulk materials like graphite. Therefore, the synthesis of silicene mainly depends on epitaxial growth on different substrates, molecular beam epitaxy (MBE), and chemical vapor deposition (CVD). Ag(111) is the most commonly used substrate, as reported in several studies since 2012.^[^ ^282^, ^284^, ^286^ ^]^ However, the high reactivity and instability of silicene under ambient conditions can introduce challenges in achieving stable growth. Additionally, degradation during the transfer process remains a major obstacle to the production of freestanding silicene. When silicene is grown on 2D layered crystals such as MoS_2_, transfer is not required. Therefore, the production of silicene via CVD, the primary challenge arises from its high reactivity, which makes it difficult to control the growth process and prevent oxidation. **Figure** **18**a ^[^ ^287^ ^]^ summarizes the production of freestanding silicene through various methods, including deposition on metals, insulators, and layered crystals^[^ ^288^, ^289^ ^]^; segregation via buffer layers^[^ ^285^ ^]^; and intercalation of metal atoms (such as Gd, Sr, and Eu) into a Si(111) substrate.^[^ ^285^ ^]^ Silver‐free silicene is unstable under ambient conditions; therefore, encapsulation is required after delamination. Degradation can occur either through the direct introduction of oxygen atoms between silicon bonds or via oxygen‐mediated amorphization due to the high reactivity of the sp^3^ bonds in the buckled silicene structure. An ultrathin Al_2_O^3^ encapsulation layer can be effective in preventing the degradation of silicene.^[^ ^290^ ^]^

- *Germanene*

Like silicene, germanene can also be grown on various metallic substrates.^[^ ^292^, ^293^, ^294^, ^295^, ^296^, ^297^, ^298^ ^]^ However, a metallic substrate can be detrimental to the 2D Dirac nature due to the hybridization of the electronic states near the Fermi level of germanium with those of the metal substrate. Zhang et al. report the growth of germanene on a semiconducting material, MoS_2,_ where the germanene layer can be coupled poorly to the MoS_2_ substrate. The grown germanene layer on MoS_2_ shows a clear 𝑉‐shaped density of states.^[^ ^299^ ^]^

Zhuang et al. reported monolayer germanene fabrication on a nonmetallic semiconducting germanium film with the support of an Ag(111) substrate. Germanene Dirac materials supported on nonmetalllic substrates are important for the QSH effect and application in FET. Besides Dirac fermion characteristics, they also theoretically predicted that the energy gap can be opened at the Brillouin zone center of the √3 × √3 restructured germanene, which indicates that the potential of the germanene nanosheet with √3 × √3 germanene can be a suitable platform for high‐speed and low‐energy‐consumption FETs.^[^ ^300^ ^]^

- *Group‐IV Xanes*

The layered, four‐coordinate puckered (buckled honeycomb) lattice of Si, Ge, or Sn is analogous to sp^3^‐hybridized graphene, where each Si, Ge, or Sn atom is terminated with H or OH groups.^[^ ^301^, ^302^ ^]^ These hydrogenated or functionalized Xenes are referred to as *Xanes*. Hydrogenation eliminates the Dirac cones at the K and K′ points in SiH, GeH, and SnH, resulting in direct band gaps at the Γ point. Hydrogenation also stabilizes the structure and helps prevent oxidation to some extent. It has been reported that multilayered Ge‐H is thermally stable up to 70 °C and undergoes slow oxidation under ambient conditions over 5 months.^[^ ^303^ ^]^ Methyl‐substituted germanane (Ge_6_(CH_3_)_6_) is highly thermally stable (up to 250 °C), and its band gap can be increased to up to 1.7 eV.^[^ ^304^ ^]^ Silicane (Si–H) or siloxane (OH and H‐terminated) and Germanane (Ge–H) exhibit semiconducting behavior with a direct band gap at the Γ point (typically ≈1.5 eV). Their high carrier mobility, direct band gap, semiconducting properties, and ease of functionalization make 2D group IV Xanes, especially germanane, due to its high stability, promising candidates for electrical and electrochemical biosensing applications.

##### Current Methods for Large‐Scale Synthesis of Group IV Xanes

Unlike Xenes, which lack bulk counterparts, layered Zintl phase materials such as CaSi_2_ and CaGe_2_ can serve as precursors for 2D Xanes.^[^ ^303^, ^305^ ^]^ These bulk materials are synthesized by annealing stoichiometric amounts of Ca with Si or Ge at high temperatures. They can then be topochemically deintercalated in aqueous HCl at low temperatures to produce multilayered, hydrogen‐terminated Si–H (silicane) and Ge–H (germanane).^[^ ^303^, ^306^ ^]^ The resulting multilayered silicane or germanane can be mechanically or liquid‐phase exfoliated into single‐ or few‐layer 2D Xanes. In the case of silicane preparation, a low temperature of around ≈30 °C is required. Silicane is a strong reducing agent; even a small amount of oxygen or moisture can oxidize it, leading to the formation of polysiloxane. At 0 °C, deintercalation of CaSi_2_ using an aqueous HCl solution results in multilayer polysiloxane sheets (Si_6_H_3_(OH)_3_).^[^ ^307^ ^]^ The synthesis of methyl germanane (Ge–CH_3_) can be achieved through topotactic deintercalation of CaGe_2_ using a methyl halide.^[^ ^308^ ^]^ In 2021, Kovalska et al. reported the synthesis of edge‐hydrogenated germanene via electrochemical exfoliation of CaGe_2_ using an electrolyte containing 0.01 m tetrabutylammonium chloride in acetonitrile. The process involved the simultaneous intercalation of tetrabutylammonium ions and decalcification of the layered CaGe_2_.^[^ ^309^ ^]^ Recently, 2D silicone has been synthesized from bulk layered CaSi_2_ by the physical vacuum distillation technique, where Ca, with a lower boiling point, is evaporized.^[^ ^310^ ^]^

##### Applications of Group IV Xenes and Xanes in Electrical and Electrochemical Biosensors

- *Group IV Xenes*

Since the band gap of silicene can be tuned using an external electric field, it holds great potential for constructing metal–oxide–semiconductor field‐effect transistors (MOSFETs) with excellent switching characteristics. Tao et al. were the first to experimentally fabricate a FET using silicene‐based 2D materials, despite its stability issues under ambient conditions, which they addressed using encapsulation and transfer techniques (Figure 18b). However, the top surface of the silicene remained unencapsulated, making it susceptible to oxidation.^[^ ^291^ ^]^ Later, Emami‐Nejad et al. fabricated a MOSFET using silicene prepared on an Ag(111)‐supported mica substrate. To protect the silicene layer, it was encapsulated with hexagonal boron nitride (h‐BN) insulating layers both above and below the silicene layer and then transferred onto a SiO_2_ substrate.^[^ ^311^ ^]^ Rahman et al. proposed a design for ion‐sensitive field‐effect transistors (ISFETs) based on silicene.^[^ ^312^ ^]^ Their theoretical simulation involved an electrolytic solution and a silicene surface layer acting as a chemically sensitive membrane for label‐free detection of DNA hybridization. The concept assumes that DNA hybridization alters the ion concentration on the silicene surface and changes the pH of the electrolyte solution. However, the practical implementation of such devices may face significant challenges, including the stability of silicene in electrolytic environments, sensitivity limitations, and overall device reliability. Currently, there is a lack of experimentally viable electrical or electrochemical biosensors based on silicene and germanene 2D Xenes, primarily due to their instability, limited‐scale synthesis, and challenges in transferring them onto suitable substrates. To advance their practical application, it is essential to develop robust synthetic methods and enhance their stability through functionalization.

- *Group IV Xanes*

**Figure 19:**
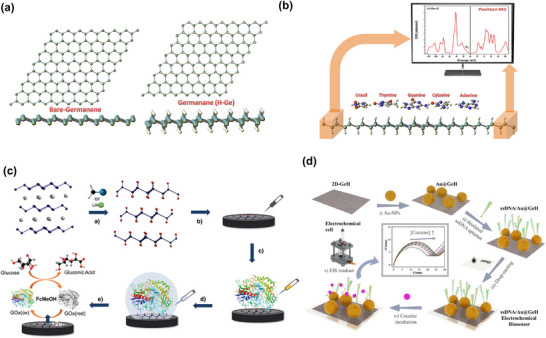
a) Monolayer germanane and germanane sheet. b) Change in the electronic properties of germanane upon adsorption of nucleobases. Reproduced with permission.^[^ ^314^ ^]^ Copyright 2018, Elsevier. c) Synthesis of germanane, methyl germanane, and siloxene by deintercalation of CaGe_2_ or CaSi_2_ using HCl or CH^3^I, along with a schematic of the fabrication process for an enzymatic electrochemical glucose sensor. Reproduced with permission.^[^ ^308^ ^]^ Copyright 2021, Wiley. d) Functionalization of Ge–H nanosheets with Au nanoparticles for the immobilization of ssDNA in the electrochemical detection of cocaine. Reproduced under the terms of the CC‐BY licence.^[^ ^316^ ^]^ Copyright 2025, Lei et al.

First‐principles DFT calculations have shown that 2D silicene and germanene sheets can bind DNA/RNA nucleobases, aromatic amino acids, and other heterocyclic molecules through physisorption and chemisorption processes. These interactions can modify the electronic properties of silicene and germanene, indicating that both silicene and germanene can be promising candidates for biomolecule sensing applications.^[^ ^313^ ^]^ Another theoretical and DFT analysis has shown that the adsorption of nucleobases on Ge–H nanosheets occurs via physisorption, primarily through van der Waals interactions. This adsorption leads to changes in the energy band structure and variations in the density of states. The adsorption affinity of nucleobases on germanane nanosheets follows the order: C > G > A > T > U (**Figure** **19**a,b). These findings suggest that Ge–H can be used as a chemiresistor and holds potential for sequencing DNA/RNA bases.^[^ ^314^ ^]^ Inspired by the various non‐covalent interactions of silicane and germanane with biomolecules, Song et al. fabricated disposable electrochemical sensors using germanane (Ge–H) and functionalized germananes (Ge–CH_3_ and Ge–CH_3_–CN) modified electrodes for the detection of Alzheimer‐related single‐nucleotide polymorphisms (SNPs).^[^ ^315^ ^]^ Among these, the Ge–H‐modified electrode showed the best performance, with a linear detection range between 1 × 10^−12^ and 1 × 10^−8^ m and a limit of detection (LOD) of 34 pM. This superior performance was attributed to the fast electron transfer rate of Ge–H for the detection of SNP. Recently, Ge–H was found to be more effective than other functionalized Germananes, such as Ge‐CH_3_ and Ge‐CH_3_‐CN, in a competitive electrochemical immunosensor for detecting gut‐derived metabolites kynurenic acid (KA) and quinolinic acid (QA).^[^ ^306^ ^]^ This superior performance was attributed to faster heterogeneous charge transfer, excellent electrocatalytic ability, lower steric hindrance, and the smaller size of Ge–H compared to Ge–CH_3_ and Ge–CH_3_–CN. The Ge–H‐based immunosensor can detect KA and QA with limits of detection (LOD) of 5.07 and 11.38 ng/mL (26.79 and 68.11 nm), respectively, demonstrating excellent reproducibility in impedimetric responses (RSD = 2.43 7.51%) and stability for up to one month at 4 °C. Chia et al. experimentally investigated the feasibility of using various group IV Xene 2D materials, such as siloxane, germanane, and methyl germanane‐ in enzymatic electrochemical biosensors for glucose detection (Figure 19c). They found that germanane exhibited superior electrocatalytic activity with faster heterogeneous electron transfer kinetics compared to other 2D Xenes.^[^ ^308^ ^]^ The germanane‐based enzymatic electrochemical biosensor demonstrated excellent performance, showing linearity over a wide range of glucose concentrations and a limit of detection (LOD) of 6.3 × 10^−6^ m. The device was also effective for detecting glucose in human serum. Very recently, Lei et al. functionalized 2D germanane (Ge–H) with Au nanoparticles (Au‐NPs) via an organometallic process, followed by covalent immobilization of single‐stranded DNA (ssDNA) through Au–S bonding (Figure 19d). Their ssDNA/Au@GeH electrochemical biosensor exhibited excellent sensitivity, selectivity, and reproducibility for the detection of the cocaine drug, with a detection limit as low as 4.9 ± 0.1 aM.^[^ ^316^ ^]^ Edge‐hydrogenated germanene has demonstrated the ability to sense volatile organic compounds that are toxic to human health, such as methanol, and exhibits selectivity for methanol over ethanol.^[^ ^309^ ^]^ A FET device was fabricated using a multilayer germanane flake, which exhibited an excellent on/off current ratio of about 10^4^ and carrier mobilities of up to 70 cm^2^ (V·s)^−1^.^[^ ^317^ ^]^ These results indicate the potential of this material for future FET‐based biosensing applications.

## 4 Outlook and Future Direction

Graphene is the most widely used 2D material for biosensing. In most FET‐based biosensors, CVD‐grown graphene is utilized due to its production of high‐quality 2D graphene nanosheets (Table 1). However, the complex instrumentation procedures, the need to control multiple parameters such as pressure, temperature, substrate, and the amount of starting reagent or gas, as well as additional transfer processes, limit its applicability in research laboratories. Since monolayer graphene lacks a band gap, bilayer graphene can be promising for FET‐based biosensors. Recently, ion intercalation synthesis techniques have been developed for bilayer graphene,^[^ ^58^ ^]^ offering further potential for FET sensing applications. The functionalization via π–π stacking creates a distance between the bioreceptor and the sensing layer, potentially reducing sensitivity. To enable more scalable and practical applications, rGO can meet the need for graphene‐like 2D materials by providing direct functionalization for bioreceptor immobilization, particularly when carboxyl‐ or hydroxyl‐functionalized GO is used. Chen et al. reported a water‐enhanced oxidation method for synthesizing GO using a modified Hummers’ method,^[^ ^59^ ^]^ which excludes sodium nitrate and reduces the amount of inorganic acids compared to the method of Marconi et al.^[^ ^75^ ^]^ This modified procedure from Chen et al.^[^ ^59^ ^]^ could be a promising solution for synthesizing GO for rGO‐based electrical and electrochemical biosensing applications

The second most widely used and promising 2D materials are the TMDs from group 6 elements (such as MoS_2_ and WS_2_). Since monolayer or few‐layered 2H MoS_2_ and WS_2_ have direct band gap semiconducting properties, they are suitable for FET‐based sensors. However, functionalization for the immobilization of bioreceptors can be challenging due to the inert nature of MoS_2_ or WS_2_. Additional dielectric materials are required for APTES functionalization on MoS_2_ nanosheets, which may reduce biosensing performance due to indirect contact with the MoS_2_ sensing layer. Recently, thiol linkers containing carboxyl (COOH) groups have been utilized to effectively adsorb onto MoS_2_ surfaces. Subsequent activation of the COOH groups through EDC/NHS chemistry allows covalent linking of bioreceptors, which can enhance the selectivity and sensitivity of electrical or electrochemical biosensors. Ion‐intercalation and exfoliation are the most established methods for obtaining 2D MoS_2_, while surfactant‐assisted liquid exfoliation may also be a viable option for biosensing, as certain surfactant functionalities on MoS_2_ nanosheets can facilitate immobilization and enable solution‐based sensor fabrication. Metallic forms of TMDs from group 6 elements, such as 1T MoS_2_ or WS_2_, could be promising for non‐enzymatic biosensors due to their effective electrocatalytic activity, although stabilizing this metastable phase is challenging.

The third important 2D material is MXene, which offers metallic conductivity, tunable surface termination groups during synthesis, and adjustable electronic properties. However, the utilization of this type of 2D material in biosensing is still in the early stages, and most developments have focused on electrochemical biosensors. Currently, MXenes are obtained by etching the MAX phase, primarily using HF. Due to some drawbacks associated with HF, various alternative processes for obtaining MXenes are being developed. MXenes with bromine (Br) surface termination groups, achieved by etching the MAX phase using molten CdBr_2_,^[^ ^140^ ^]^ may hold potential for biosensing applications, especially considering the further modification of Br to oxygen, sulfur, selenium, tellurium, and NH groups, which can be effective for bioreceptor immobilization. Additionally, ion‐intercalated (TBA^+^) or APTES‐functionalized MXene nanosheets may be promising for both FET and electrochemical biosensors due to their band gap, increased interlayer spacing, and ease of immobilization through covalent and hydrogen bonding.

2D MOFs and COFs have potential for electrochemical and non‐enzymatic biosensing due to their porous structure, moderate electrical conductivity, chemical stability, and the advantages of their 2D nature, which promote significant electrocatalytic activity. However, synthesis in 2D form and improvement of conductivity remain challenging issues for the utilization of these types of 2D materials in biosensing applications. Currently, the use of MOFs and COFs in biosensing is still in its early stages. Various wet chemical synthesis methods for MOFs and COFs^[^ ^33^, ^156^, ^191^ ^]^ with diverse chemical functionalities are well‐established but need further exploration and optimization for biosensing applications.

2D TMOs are also promising for biosensing, particularly as electrocatalysts for non‐enzymatic biosensing. There is a variety of 2D TMOs, each with unique physical, chemical, and electronic properties. The semiconducting α‐MoO_3_ also shows promise as a channel material for FET‐based sensors. Overall, this class of 2D materials holds potential for both FET and electrochemical biosensors. Further exploration of various layered TMOs as sensing layers in electrical and electrochemical biosensors, along with optimization of functionalization and immobilization techniques, is needed. 2D LDHs hold potential as electrocatalysts for biomolecule sensing in non‐enzymatic electrochemical sensors. The positively charged layers of LDHs can interact with negatively charged bioreceptors, facilitating effective bioreceptor immobilization. However, the poor conductivity of LDHs may limit their use as a standalone sensing layer in electrochemical biosensors.

2D g‐C_3_N_4_ may be more suitable for photoelectrochemical biosensors due to its excellent photocatalytic ability. However, it can also be used as an electrocatalyst in non‐enzymatic reactions. As with other 2D materials, bioreceptor immobilization on this sensing layer is not yet well‐established.

h‐BN can be used as a gate dielectric due to its insulating properties and inertness. However, defects introduced during synthesis and exfoliation can lower the band gap and enhance its electrocatalytic ability, making this 2D nanomaterial suitable for use in non‐enzymatic electrochemical biosensing.

The direct bandgap semiconducting properties and excellent carrier mobility of black phosphorus/phosphorene make it a promising 2D material for FET devices. Common exfoliation techniques used for 2D layered materials from their bulk form are also suitable for black phosphorus. However, commercial bulk phosphorus is quite expensive, and functionalization for immobilization and stability may be challenging for the use of this type of 2D material in biosensing.

Borophene has potential as a candidate for electrochemical biosensing due to its metallic characteristics. However, its stability remains a significant concern for practical applications. Theoretically, semiconducting and stable phases of borophene, including hydrogenated borophene, appear more promising for applications in FETs and electrochemical biosensors. Nevertheless, the immobilization of bioreceptors on borophene surfaces is not yet well established. Further studies are needed to investigate functionalization strategies and potential applications. Another key challenge is the synthesis of atomically thin borophene, which currently relies on techniques such as CVD and MBE, both of which are unsuitable for scalable production. Hydrogenated borophene is likely a more suitable candidate for biosensing materials in terms of stability, semiconducting properties, and ease of functionalization.

Buckled honeycomb‐structured 2D materials such as silicene and germanene, with their semimetallic behavior, strong SOC, and ease of bandgap tuning, show great promise for flexible electronic devices. However, their stability under ambient conditions raises concerns for both synthesis and the practical application of electrical and electrochemical devices. Although insulating layers like h‐BN or dielectric Al_2_O_3_ layer can protect germanene nanosheets and facilitate functionalization using APTES, as demonstrated with MoS_2_ for bioreceptor attachment, device fabrication with these 2D Xenes remains challenging.

Hydrogenated Xenes, such as germanane, offer a more promising alternative for electrical and electrochemical biosensing due to their excellent stability, semiconducting behavior with a tunable bandgap, and ease of functionalization for bioreceptor immobilization. Nevertheless, there is still a lack of sufficient experimental data on these potential sensing elements for practical biosensing applications. Further exploration is needed to address aspects such as functionalization strategies, covalent bonding with biomolecules, and scalable synthesis methods.

**Figure 20:**
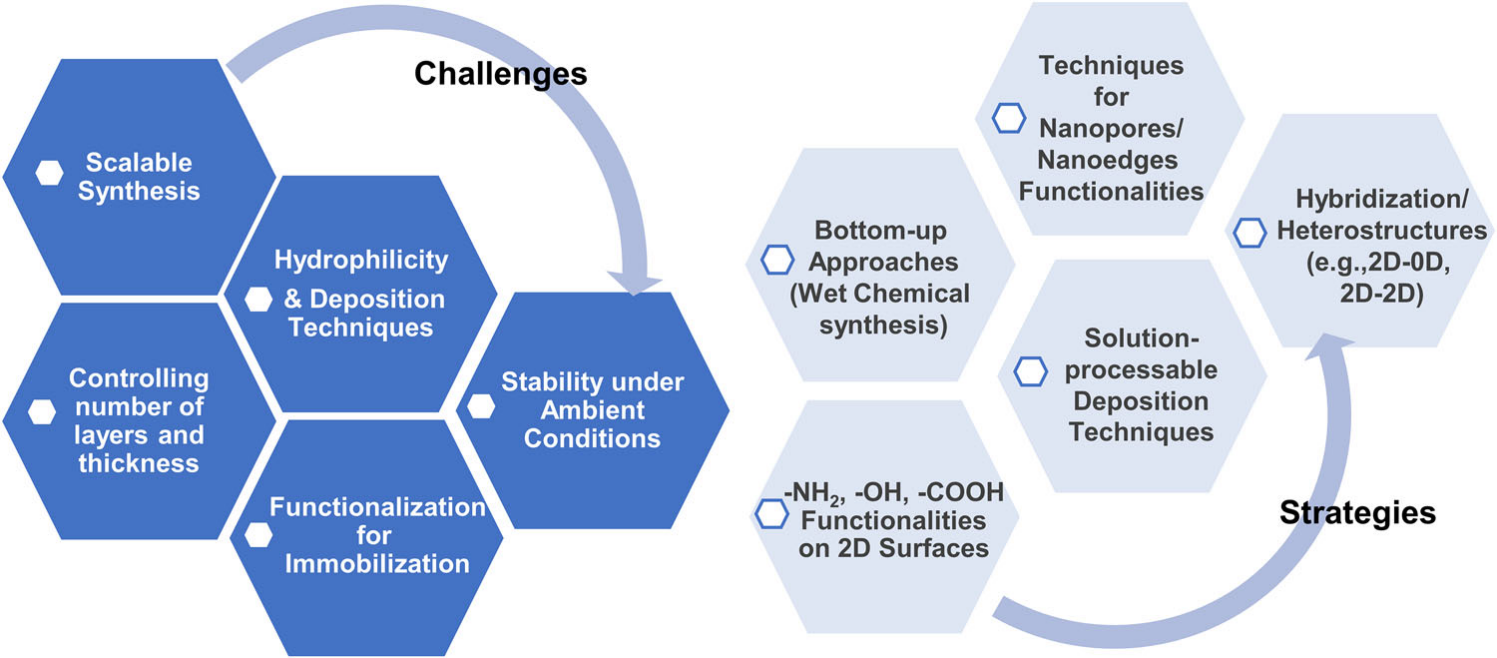
Key challenges associated with 2D materials and possible strategies to overcome these challenges.

Significant progress has been made over the last three decades in the development of electrical and electrochemical biosensors by exploring various types of 2D materials as sensing elements. However, several challenges remain for the practical implementation of 2D materials in biosensing (**Figure** **20**). These include scalable synthesis, thin film fabrication on suitable substrates, functionalization for bioreceptor immobilization to enable selective detection of target biomolecules, and maintaining stability under ambient conditions.

These challenges can be addressed by developing efficient wet chemical bottom‐up synthetic methods, introducing various functional groups, particularly carboxyl (─COOH), hydroxyl (─OH), and amine (─NH_2_) groups that can covalently link bioreceptors, and tuning interlayer distances through the intercalation of functional molecules to enhance bioreceptor immobilization. Additionally, techniques such as creating nanopores with nanoedge functionalities may further improve the performance and selectivity of 2D material‐based biosensors by enhancing bioreceptor immobilization. Another strategy could be the utilization and development of hybrid nanomaterials, such as 2D‐2D and 2D‐0D nanoparticle hybrids, as well as 2D–2D heterostructures, which may enhance electrochemical properties, improve electrocatalytic activity, and facilitate bioreceptor immobilization through various interactions.

## 5 Conclusion

Electrical or electrochemical biosensors possess significant advantages, such as high sensitivity and versatility, making them widely applicable. By incorporating 2D nanomaterials into the biosensors, their performance can be enhanced while reducing costs, thus enabling the widespread adoption of point‐of‐care diagnostic devices. However, achieving large‐scale synthesis and effective functionalization of these materials remains one of the most challenging and critical factors for realizing these advancements. This review focuses on addressing these challenges by discussing various types of 2D nanomaterials. While considerable progress has been made in biosensing applications using some common 2D materials, many emerging 2D materials remain unexplored for efficient biosensing applications. Additionally, a significant gap exists between material preparation and device implementation. This review covers the preparation methods of 2D materials developed over the past two decades, emphasizing the link between material synthesis and practical applications. All 2D materials demonstrate potential for electrical or electrochemical biosensing or both following proper optimization, functionalization, and hybridization. The diverse 2D materials discussed here offer a rich toolkit for advancing the development of cost‐effective and scalable electrical and electrochemical biosensors for disease diagnosis, through optimization via hybridization, doping, and functionalization.

## Conflict of Interest

The authors declare no conflict of interest.
